# Supplementary material for: Robotic and laparoscopic gynaecological surgery: a prospective multicentre observational cohort study and economic evaluation in England
Source: BMJ Open. 2023 Sep 28;13(9):e073990. doi: 10.1136/bmjopen-2023-073990 (PMC10546163; doi:10.1136/bmjopen-2023-073990)
Supplement: Supplementary data [file bmjopen-2023-073990supp001.pdf]

Supplemental material 1: Resource Use

Table S1.1: Resource use for surgical procedure

Resource or unit intervention	Unit	Mean usage in standard laparoscopic	Mean usage in standard robotic	Resource source
**Fixed theatre usage overheads**				
Operating theatre	Average theatre duration	168 minutes	183 minutes	RoBoQoL study procedure CRF form
**Staff**				
**Resource or unit intervention**	**Unit**	**Mean usage in standard laparoscopic**	**Mean usage in standard robotic**	**Resource source**
Consultant surgeon	Per hour	1	1	South Tees Hospitals NHS Foundation based on personal communication with Jeremy Twigg and Alison Waines between Jan-Nov 2020
Assistant surgeon (registrar)	Per hour	1	1	South Tees Hospitals NHS Foundation based on personal communication with Jeremy Twigg and Alison Waines between Jan-Nov 2020
Consultant anaesthetist	Per hour	1	1	South Tees Hospitals NHS Foundation based on personal communication with Jeremy Twigg and Alison Waines between Jan-Nov 2020
Anaesthetist registrar	Per hour	1	1	South Tees Hospitals NHS Foundation based on personal

				communication with Jeremy Twigg and Alison Waines between Jan-Nov 2020
Anaesthetist nurse	Per hour	1	1	South Tees Hospitals NHS Foundation based on personal communication with Jeremy Twigg and Alison Waines between Jan-Nov 2020
Nurse	Per hour	1	1	South Tees Hospitals NHS Foundation based on personal communication with Jeremy Twigg and Alison Waines between Jan-Nov 2020
Health care assistant	Per hour	1	1	South Tees Hospitals NHS Foundation based on personal communication with Jeremy Twigg and Alison Waines between Jan-Nov 2020
Recovery practitioner	Per hour	1	1	South Tees Hospitals NHS Foundation based on personal communication with Jeremy Twigg and Alison Waines between Jan-Nov 2020
**Capital equipment**				
**Resource or unit intervention**	**Unit**	**Mean usage in standard laparoscopic**	**Mean usage in standard robotic**	**Resource source**

Laparoscopic device (slave screens included)	Per procedure	1	1	South Tees Hospitals NHS Foundation based on personal communication with Jeremy Twigg and Alison Waines between Jan-Nov 2020
Robotic device	Per procedure	1	1	South Tees Hospitals NHS Foundation based on personal communication with Jeremy Twigg and Alison Waines between Jan-Nov 2020
Diathermy console	Per procedure	1	1	South Tees Hospitals NHS Foundation based on personal communication with Jeremy Twigg and Alison Waines between Jan-Nov 2020
Anaesthetic machine	Per procedure	1	1	South Tees Hospitals NHS Foundation based on personal communication with Jeremy Twigg and Alison Waines between Jan-Nov 2020
PCA pump	Per procedure	1	1	South Tees Hospitals NHS Foundation based on personal communication with Jeremy Twigg and Alison Waines between Jan-Nov 2020

Suction machine	Per procedure	1	1	South Tees Hospitals NHS Foundation based on personal communication with Jeremy Twigg and Alison Waines between Jan-Nov 2020
**Consumables**<br>**Surgical equipment specific to laparoscopic and robotic surgery**				
**Resource or unit intervention**	**Unit**	**Mean usage in standard laparoscopic**	**Mean usage in standard robotic**	**Resource source**
Gel port/trocar	Per procedure	1	n/a	South Tees Hospitals NHS Foundation based on personal communication with Jeremy Twigg and Alison Waines between Jan-Nov 2020
55mm dual port/trocar	Per procedure	4	n/a	South Tees Hospitals NHS Foundation based on personal communication with Jeremy Twigg and Alison Waines between Jan-Nov 2020
11mm port/trocar	Per procedure	1	n/a	South Tees Hospitals NHS Foundation based on personal communication with Jeremy Twigg and Alison Waines between Jan-Nov 2020
Verris lap pack	Per procedure	1	n/a	South Tees Hospitals NHS Foundation based on personal communication

				with Jeremy Twigg and Alison Waines between Jan-Nov 2020
Vcare manipulator	Per procedure	1	1	South Tees Hospitals NHS Foundation based on personal communication with Jeremy Twigg and Alison Waines between Jan-Nov 2020
Applied scissors	Per procedure	1	n/a	South Tees Hospitals NHS Foundation based on personal communication with Jeremy Twigg and Alison Waines between Jan-Nov 2020
Harmonic (ethicon)	Per procedure	1	n/a	South Tees Hospitals NHS Foundation based on personal communication with Jeremy Twigg and Alison Waines between Jan-Nov 2020
Misc. forceps	Per procedure	1	n/a	South Tees Hospitals NHS Foundation based on personal communication with Jeremy Twigg and Alison Waines between Jan-Nov 2020
Vlock suture	Per procedure	1	1	South Tees Hospitals NHS Foundation based on personal communication with Jeremy

				Twigg and Alison Waines between Jan-Nov 2020
Endoclose	Per procedure	1	1	South Tees Hospitals NHS Foundation based on personal communication with Jeremy Twigg and Alison Waines between Jan-Nov 2020
Syringes	Per procedure	1	1	South Tees Hospitals NHS Foundation based on personal communication with Jeremy Twigg and Alison Waines between Jan-Nov 2020
Scissors (robot specific)	Per procedure	1	1	South Tees Hospitals NHS Foundation based on personal communication with Jeremy Twigg and Alison Waines between Jan-Nov 2020
Pro-grasp	Per procedure	1	1	South Tees Hospitals NHS Foundation based on personal communication with Jeremy Twigg and Alison Waines between Jan-Nov 2020
Maryland bi-polar	Per procedure	1	1	South Tees Hospitals NHS Foundation based on personal communication with Jeremy Twigg and Alison

				Waines between Jan-Nov 2020
Needle holder	Per procedure	1	1	South Tees Hospitals NHS Foundation based on personal communication with Jeremy Twigg and Alison Waines between Jan-Nov 2020
Scope sterilisation for 30 degrees & 0 degrees	Per procedure	1	1	South Tees Hospitals NHS Foundation based on personal communication with Jeremy Twigg and Alison Waines between Jan-Nov 2020
Accessory set sterilisation	Per procedure	n/a	2	South Tees Hospitals NHS Foundation based on personal communication with Jeremy Twigg and Alison Waines between Jan-Nov 2020
**General consumable surgical equipment**				
**Resource or unit intervention**	**Unit**	**Mean usage in standard laparoscopic**	**Mean usage in standard robotic**	**Resource source**
Arterial line	Per procedure	1	1	South Tees Hospitals NHS Foundation based on personal communication with Jeremy Twigg, Alison Waines and Richard Bickle between Jan-Nov 2020
Central line	Per procedure	1	1	South Tees Hospitals NHS

				Foundation based on personal communication with Jeremy Twigg, Alison Waines and Richard Bickle between Jan-Nov 2020
12F catheter	Per procedure	1	1	South Tees Hospitals NHS Foundation based on personal communication with Jeremy Twigg, Alison Waines and Richard Bickle between Jan-Nov 2020
Urine drainage bag	Per procedure	1	1	South Tees Hospitals NHS Foundation based on personal communication with Jeremy Twigg, Alison Waines and Richard Bickle between Jan-Nov 2020
Monocryl	Per procedure	4	4	South Tees Hospitals NHS Foundation based on personal communication with Jeremy Twigg, Alison Waines and Richard Bickle between Jan-Nov 2020
Bair Hugger warmer	Per procedure	1	1	South Tees Hospitals NHS Foundation based on personal

				communication with Jeremy Twigg, Alison Waines and Richard Bickle between Jan-Nov 2020
Fluid warmer	Per procedure	1	1	South Tees Hospitals NHS Foundation based on personal communication with Jeremy Twigg, Alison Waines and Richard Bickle between Jan-Nov 2020
Drape	Per procedure	1	n/a	South Tees Hospitals NHS Foundation based on personal communication with Jeremy Twigg, Alison Waines and Richard Bickle between Jan-Nov 2020
4 arm drape	Per procedure	n/a	1	South Tees Hospitals NHS Foundation based on personal communication with Jeremy Twigg, Alison Waines and Richard Bickle between Jan-Nov 2020
**Scissor tip cover**	Per procedure	n/a	1	South Tees Hospitals NHS Foundation based on personal communication with Jeremy Twigg, Alison

				Waines and Richard Bickle between Jan-Nov 2020
**Blood products**				
**Resource or unit intervention**	**Unit**	**Mean usage in standard laparoscopic**	**Mean usage in standard robotic**	**Resource source**
Standard red blood cells	Per procedure	0.06 units	0.07 units	RoBoQoL study procedure CRF form
**Other resources**				
Anaesthetic equipment	Per procedure	1	1	South Tees Hospitals NHS Foundation based on personal communication with Richard Bickle between Jan-Nov 2020
Anaesthetic drugs	Per procedure	1	1	South Tees Hospitals NHS Foundation based on personal communication with Richard Bickle between Jan-Nov 2020
IV fluids	Per procedure	5	5	South Tees Hospitals NHS Foundation based on personal communication with Richard Bickle between Jan-Nov 2020
**Type of stay**				
ICU	Per day	0.07	0.08	RoBoQoL study procedure CRF
Ward	Per day	2.22	1.28	RoBoQoL study procedure CRF

Table S1.2: Resource use during follow-up

Resource or unit intervention		measure	RALS (n=159)	CLS (n=73)	Source
Week 1					
**Primary Care**					
General Practitioner (GP)	Home Visit	Mean (SD)	0.01 (0.09)	0 (0)	RoboQoL CRF
		Missing (N%)	39 (24.5)	6 (9)	
	Surgery Visit	Mean (SD)	0.13 (0.4)	0.05 (0.21)	RoboQoL CRF
		Missing (N%)	41 (25.8)	7 (10.6)	
	Phone Appointment	Mean (SD)	0.08 (0.3)	0.09 (0.29)	RoboQoL CRF
		Missing (N%)	40 (25.2)	6 (9)	
Nurse	Home Visit	Mean (SD)	0.16 (0.96)	0 (0)	RoboQoL CRF
		Missing (N%)	40 (25.2)	6 (9)	
	Surgery Visit	Mean (SD)	0.05 (0.22)	0.04 (0.27)	RoboQoL CRF
		Missing (N%)	41 (25.8)	6 (9)	
	Phone Appointment	Mean (SD)	0.03 (0.16)	0.03 (0.17)	RoboQoL CRF
		Missing (N%)	39 (24.5)	6 (9)	
Occupational Therapist (OT)	Home Visit	Mean (SD)	0 (0)	0 (0)	RoboQoL CRF
		Missing (N%)	39 (24.5)	7 (10.6)	
	Surgery Visit	Mean (SD)	0 (0)	0.05 (0.37)	RoboQoL CRF
		Missing (N%)	39 (24.5)	7 (10.6)	
	Hospital Visit	Mean (SD)	0 (0)	0 (0)	RoboQoL CRF
		Missing (N%)	39 (24.5)	7 (10.6)	
Physiotherapist	Home Visit	Mean (SD)	0 (0)	0 (0)	RoboQoL CRF
		Missing (N%)	39 (24.5)	6 (9)	
	Surgery Visit	Mean (SD)	39 (0)	6 (0)	RoboQoL CRF
		Missing (N%)	39 (24.5)	6 (9)	
	Phone Appointment	Mean (SD)	0.1 (0.4)	0.22 (0.42)	RoboQoL CRF
		Missing (N%)	39 (24.5)	6 (9)	
	Hospital Visit	Mean (SD)	0 (0)	0 (0)	RoboQoL CRF
		Missing (N%)	39 (24.5)	6 (9)	
	Day Visit	Mean (SD)	0 (0)	0 (0)	RoboQoL CRF
		Missing (N%)	39 (24.5)	6 (9)	
**Secondary Care**					
Outpatient Appointment		Mean (SD)	0.1 (0.1)	0.07 (0.07)	RoboQoL CRF
		Missing (N%)	39 (24.5)	6 (9)	
Ambulance See and treat		Mean (SD)	0.01 (0.09)	0 (0)	RoboQoL CRF
		Missing (N%)	39 (24.5)	6 (9)	
Ambulance see and convey		Mean (SD)	0 (0)	0 (0)	RoboQoL CRF
		Missing (N%)	39 (24.5)	6 (9)	
A&E		Mean (SD)	0.08 (0.66)	0.03 (0.17)	RoboQoL CRF
		Missing (N%)	41 (25.8)	6 (9)	
Hospital Ward Day		Mean (SD)	0.05 (0.26)	0.04 (0.21)	RoboQoL CRF
		Missing (N%)	41 (25.8)	6 (9)	
Hospital Overnight Visit		Mean (SD)	0.15 (0.65)	0.58 (1.55)	RoboQoL CRF
		Missing (N%)	40 (25.2)	6 (9)	

Week 2					
**Primary Care**					
General Practitioner (GP)	Home Visit	Mean (SD)	0.02 (0.18)	0 (0)	RoboQoL CRF
		Missing (N%)	42 (26.4)	3 (4.3)	
	Surgery Visit	Mean (SD)	0.07 (0.25)	0.04 (0.2)	RoboQoL CRF
		Missing (N%)	42 (26.4)	3 (4.3)	
	Phone Appointment	Mean (SD)	0.09 (0.35)	0.07 (0.26)	RoboQoL CRF
		Missing (N%)	42 (26.4)	3 (4.3)	
Nurse	Home Visit	Mean (SD)	0.14 (0.93)	0.01 (0.12)	RoboQoL CRF
		Missing (N%)	43 (27)	4 (5.8)	
	Surgery Visit	Mean (SD)	0.1 (0.31)	0.01 (0.12)	RoboQoL CRF
		Missing (N%)	43 (27)	6 (9)	
	Phone Appointment	Mean (SD)	0.03 (0.21)	0 (0)	RoboQoL CRF
		Missing (N%)	43 (27)	4 (5.8)	
Occupational Therapist (OT)	Home Visit	Mean (SD)	0 (0)	0 (0)	RoboQoL CRF
		Missing (N%)	43 (27)	4 (5.8)	
	Surgery Visit	Mean (SD)	0 (0)	0 (0)	RoboQoL CRF
		Missing (N%)	44 (27.7)	4 (5.8)	
	Hospital Visit	Mean (SD)	0 (0)	0 (0)	RoboQoL CRF
		Missing (N%)	43 (27)	4 (5.8)	
Physiotherapist	Home Visit	Mean (SD)	0 (0)	0 (0)	RoboQoL CRF
		Missing (N%)	42 (26.4)	4 (5.8)	
	Surgery Visit	Mean (SD)	42 (0)	4 (0)	RoboQoL CRF
		Missing (N%)	42 (26.4)	4 (5.8)	
	Phone Appointment	Mean (SD)	0 (0)	0.03 (0.17)	RoboQoL CRF
		Missing (N%)	42 (26.4)	4 (5.8)	
	Hospital Visit	Mean (SD)	0 (0)	0 (0)	RoboQoL CRF
		Missing (N%)	42 (26.4)	4 (5.8)	
	Day Visit	Mean (SD)	0 (0)	0 (0)	RoboQoL CRF
		Missing (N%)	42 (26.4)	4 (5.8)	
**Secondary Care**					
Outpatient Appointment		Mean (SD)	0.21 (0.21)	0.12 (0.12)	RoboQoL CRF
		Missing (N%)	39 (24.5)	4 (5.8)	
Ambulance See and treat		Mean (SD)	0.01 (0.09)	0.03 (0.17)	RoboQoL CRF
		Missing (N%)	42 (26.4)	3 (4.3)	
Ambulance see and convey		Mean (SD)	0.01 (0.09)	0.03 (0.17)	RoboQoL CRF
		Missing (N%)	42 (26.4)	3 (4.3)	
A&E		Mean (SD)	0.03 (0.18)	0.03 (0.17)	RoboQoL CRF
		Missing (N%)	42 (26.4)	3 (4.3)	
Hospital Ward Day		Mean (SD)	0.03 (0.16)	0.01 (0.12)	RoboQoL CRF
		Missing (N%)	42 (26.4)	4 (5.8)	
Hospital Overnight Visit		Mean (SD)	0.12 (0.71)	0.34 (1.38)	RoboQoL CRF
		Missing (N%)	42 (26.4)	3 (4.3)	
Week 3					
**Primary Care**					

General Practitioner (GP)	Home Visit	Mean (SD)	0 (0)	0 (0)	RoboQoL CRF
		Missing (N%)	44 (27.7)	11 (17.7)	
	Surgery Visit	Mean (SD)	0.11 (0.35)	0.06 (0.25)	RoboQoL CRF
		Missing (N%)	45 (28.3)	11 (17.7)	
	Phone Appointment	Mean (SD)	0.07 (0.29)	0.03 (0.18)	RoboQoL CRF
		Missing (N%)	44 (27.7)	11 (17.7)	
Nurse	Home Visit	Mean (SD)	0.14 (0.94)	0 (0)	RoboQoL CRF
		Missing (N%)	44 (27.7)	11 (17.7)	
	Surgery Visit	Mean (SD)	0.09 (0.34)	0.05 (0.22)	RoboQoL CRF
		Missing (N%)	44 (27.7)	11 (17.7)	
	Phone Appointment	Mean (SD)	0.03 (0.16)	0.03 (0.18)	RoboQoL CRF
		Missing (N%)	44 (27.7)	11 (17.7)	
Occupational Therapist (OT)	Home Visit	Mean (SD)	0 (0)	0 (0)	RoboQoL CRF
		Missing (N%)	44 (27.7)	11 (17.7)	
	Surgery Visit	Mean (SD)	0 (0)	0.03 (0.25)	RoboQoL CRF
		Missing (N%)	44 (27.7)	11 (17.7)	
	Hospital Visit	Mean (SD)	0 (0)	0 (0)	RoboQoL CRF
		Missing (N%)	44 (27.7)	11 (17.7)	
Physiotherapist	Home Visit	Mean (SD)	0 (0)	0 (0)	RoboQoL CRF
		Missing (N%)	45 (28.3)	11 (17.7)	
	Surgery Visit	Mean (SD)	45 (0)	11 (0)	RoboQoL CRF
		Missing (N%)	45 (28.3)	11 (17.7)	
	Phone Appointment	Mean (SD)	0.02 (0.13)	0 (0)	RoboQoL CRF
		Missing (N%)	45 (28.3)	11 (17.7)	
	Hospital Visit	Mean (SD)	0.01 (0.09)	0 (0)	RoboQoL CRF
		Missing (N%)	45 (28.3)	11 (17.7)	
	Day Visit	Mean (SD)	0 (0)	0 (0)	RoboQoL CRF
		Missing (N%)	45 (28.3)	11 (17.7)	
**Secondary Care**					
Outpatient Appointment		Mean (SD)	0.3 (0.3)	0.19 (0.19)	RoboQoL CRF
		Missing (N%)	43 (27)	10 (15.9)	
Ambulance See and treat		Mean (SD)	0 (0)	0 (0)	RoboQoL CRF
		Missing (N%)	45 (28.3)	11 (17.7)	
Ambulance see and convey		Mean (SD)	0 (0)	0 (0)	RoboQoL CRF
		Missing (N%)	45 (28.3)	11 (17.7)	
A&E		Mean (SD)	0.01 (0.09)	0 (0)	RoboQoL CRF
		Missing (N%)	45 (28.3)	11 (17.7)	
Hospital Ward Day		Mean (SD)	0 (0)	0.02 (0.13)	RoboQoL CRF
		Missing (N%)	45 (28.3)	11 (17.7)	
Hospital Overnight Visit		Mean (SD)	0.05 (0.26)	0.16 (1.15)	RoboQoL CRF
		Missing (N%)	45 (28.3)	11 (17.7)	
Week 4					
**Primary Care**					
General Practitioner (GP)	Home Visit	Mean (SD)	0.01 (0.09)	0.02 (0.13)	RoboQoL CRF
		Missing (N%)	44 (27.7)	10 (15.9)	

	Surgery Visit	Mean (SD)	0.08 (0.27)	0.1 (0.35)	RoboQoL CRF
		Missing (N%)	46 (28.9)	10 (15.9)	
	Phone Appointment	Mean (SD)	0.04 (0.2)	0.03 (0.25)	RoboQoL CRF
		Missing (N%)	44 (27.7)	10 (15.9)	
Nurse	Home Visit	Mean (SD)	0.09 (0.66)	0 (0)	RoboQoL CRF
		Missing (N%)	46 (28.9)	10 (15.9)	
	Surgery Visit	Mean (SD)	0.04 (0.25)	0.06 (0.25)	RoboQoL CRF
		Missing (N%)	46 (28.9)	10 (15.9)	
	Phone Appointment	Mean (SD)	0 (0)	0 (0)	RoboQoL CRF
		Missing (N%)	46 (28.9)	10 (15.9)	
Occupational Therapist (OT)	Home Visit	Mean (SD)	0 (0)	0 (0)	RoboQoL CRF
		Missing (N%)	45 (28.3)	10 (15.9)	
	Surgery Visit	Mean (SD)	0.01 (0.09)	0 (0)	RoboQoL CRF
		Missing (N%)	45 (28.3)	10 (15.9)	
	Hospital Visit	Mean (SD)	0 (0)	0 (0)	RoboQoL CRF
		Missing (N%)	45 (28.3)	10 (15.9)	
Physiotherapist	Home Visit	Mean (SD)	0 (0)	0 (0)	RoboQoL CRF
		Missing (N%)	47 (29.6)	10 (15.9)	
	Surgery Visit	Mean (SD)	47 (0)	10 (0)	RoboQoL CRF
		Missing (N%)	47 (29.6)	10 (15.9)	
	Phone Appointment	Mean (SD)	0 (0)	0 (0)	RoboQoL CRF
		Missing (N%)	48 (30.2)	10 (15.9)	
	Hospital Visit	Mean (SD)	0.01 (0.09)	0 (0)	RoboQoL CRF
		Missing (N%)	47 (29.6)	10 (15.9)	
	Day Visit	Mean (SD)	0 (0)	0 (0)	RoboQoL CRF
		Missing (N%)	47 (29.6)	10 (15.9)	
**Secondary Care**					
Outpatient Appointment		Mean (SD)	0.34 (0.34)	0.11 (0.11)	RoboQoL CRF
		Missing (N%)	46 (28.9)	8 (12.3)	
Ambulance See and treat		Mean (SD)	0.01 (0.09)	0 (0)	RoboQoL CRF
		Missing (N%)	45 (28.3)	9 (14.1)	
Ambulance see and convey		Mean (SD)	0 (0)	0 (0)	RoboQoL CRF
		Missing (N%)	45 (28.3)	9 (14.1)	
A&E		Mean (SD)	0.01 (0.09)	0 (0)	RoboQoL CRF
		Missing (N%)	45 (28.3)	9 (14.1)	
Hospital Ward Day		Mean (SD)	0 (0)	0.02 (0.13)	RoboQoL CRF
		Missing (N%)	45 (28.3)	9 (14.1)	
Hospital Overnight Visit		Mean (SD)	0.01 (0.09)	0.02 (0.13)	RoboQoL CRF
		Missing (N%)	45 (28.3)	9 (14.1)	
Week 5					
**Primary Care**					
General Practitioner (GP)	Home Visit	Mean (SD)	0 (0)	0 (0)	RoboQoL CRF
		Missing (N%)	56 (35.2)	14 (23.7)	
	Surgery Visit	Mean (SD)	0.1 (0.33)	0.07 (0.25)	RoboQoL CRF
		Missing (N%)	57 (35.8)	14 (23.7)	

	Phone Appointment	Mean (SD)	0.04 (0.2)	0.05 (0.22)	RoboQoL CRF
		Missing (N%)	57 (35.8)	14 (23.7)	
Nurse	Home Visit	Mean (SD)	0 (0)	0 (0)	RoboQoL CRF
		Missing (N%)	57 (35.8)	14 (23.7)	
	Surgery Visit	Mean (SD)	0.09 (0.32)	0.08 (0.28)	RoboQoL CRF
		Missing (N%)	57 (35.8)	14 (23.7)	
	Phone Appointment	Mean (SD)	0.01 (0.1)	0 (0)	RoboQoL CRF
		Missing (N%)	57 (35.8)	14 (23.7)	
Occupational Therapist (OT)	Home Visit	Mean (SD)	0 (0)	0 (0)	RoboQoL CRF
		Missing (N%)	57 (35.8)	15 (25.9)	
	Surgery Visit	Mean (SD)	0 (0)	0 (0)	RoboQoL CRF
		Missing (N%)	57 (35.8)	15 (25.9)	
	Hospital Visit	Mean (SD)	0 (0)	0 (0)	RoboQoL CRF
		Missing (N%)	57 (35.8)	15 (25.9)	
Physiotherapist	Home Visit	Mean (SD)	0 (0)	0 (0)	RoboQoL CRF
		Missing (N%)	56 (35.2)	15 (25.9)	
	Surgery Visit	Mean (SD)	56 (0)	15 (0)	RoboQoL CRF
		Missing (N%)	56 (35.2)	15 (25.9)	
	Phone Appointment	Mean (SD)	0 (0)	0 (0)	RoboQoL CRF
		Missing (N%)	56 (35.2)	15 (25.9)	
	Hospital Visit	Mean (SD)	0 (0)	0 (0)	RoboQoL CRF
		Missing (N%)	56 (35.2)	15 (25.9)	
	Day Visit	Mean (SD)	0 (0)	0 (0)	RoboQoL CRF
		Missing (N%)	56 (35.2)	15 (25.9)	
**Secondary Care**					
Outpatient Appointment		Mean (SD)	0.26 (0.26)	0.1 (0.1)	RoboQoL CRF
		Missing (N%)	57 (35.8)	14 (23.7)	
Ambulance See and treat		Mean (SD)	0 (0)	0 (0)	RoboQoL CRF
		Missing (N%)	59 (37.1)	14 (23.7)	
Ambulance see and convey		Mean (SD)	0 (0)	0 (0)	RoboQoL CRF
		Missing (N%)	59 (37.1)	14 (23.7)	
A&E		Mean (SD)	0.02 (0.14)	0 (0)	RoboQoL CRF
		Missing (N%)	56 (35.2)	14 (23.7)	
Hospital Ward Day		Mean (SD)	0 (0)	0.02 (0.13)	RoboQoL CRF
		Missing (N%)	56 (35.2)	14 (23.7)	
Hospital Overnight Visit		Mean (SD)	0 (0)	0.02 (0.13)	RoboQoL CRF
		Missing (N%)	56 (35.2)	14 (23.7)	
Week 6					
**Primary Care**					
General Practitioner (GP)	Home Visit	Mean (SD)	0 (0)	0.02 (0.13)	RoboQoL CRF
		Missing (N%)	55 (34.6)	14 (23.7)	
	Surgery Visit	Mean (SD)	0.16 (0.37)	0.09 (0.29)	RoboQoL CRF
		Missing (N%)	55 (34.6)	16 (28.1)	
	Phone Appointment	Mean (SD)	0.04 (0.19)	0.03 (0.18)	RoboQoL CRF
		Missing (N%)	55 (34.6)	13 (21.7)	

Nurse	Home Visit	Mean (SD)	0 (0)	0.03 (0.18)	RoboQoL CRF
		Missing (N%)	55 (34.6)	13 (21.7)	
	Surgery Visit	Mean (SD)	0.01 (0.1)	0.03 (0.18)	RoboQoL CRF
		Missing (N%)	55 (34.6)	14 (23.7)	
	Phone Appointment	Mean (SD)	0 (0)	0.05 (0.22)	RoboQoL CRF
		Missing (N%)	55 (34.6)	13 (21.7)	
Occupational Therapist (OT)	Home Visit	Mean (SD)	0 (0)	0 (0)	RoboQoL CRF
		Missing (N%)	55 (34.6)	13 (21.7)	
	Surgery Visit	Mean (SD)	0 (0)	0 (0)	RoboQoL CRF
		Missing (N%)	55 (34.6)	13 (21.7)	
	Hospital Visit	Mean (SD)	0 (0)	0 (0)	RoboQoL CRF
		Missing (N%)	55 (34.6)	13 (21.7)	
Physiotherapist	Home Visit	Mean (SD)	0 (0)	0 (0)	RoboQoL CRF
		Missing (N%)	55 (34.6)	13 (21.7)	
	Surgery Visit	Mean (SD)	55 (0)	13 (0)	RoboQoL CRF
		Missing (N%)	55 (34.6)	13 (21.7)	
	Phone Appointment	Mean (SD)	0 (0)	0 (0)	RoboQoL CRF
		Missing (N%)	55 (34.6)	13 (21.7)	
	Hospital Visit	Mean (SD)	0.01 (0.1)	0 (0)	RoboQoL CRF
		Missing (N%)	55 (34.6)	13 (21.7)	
	Day Visit	Mean (SD)	0 (0)	0 (0)	RoboQoL CRF
		Missing (N%)	55 (34.6)	13 (21.7)	
**Secondary Care**					
Outpatient Appointment		Mean (SD)	0.31 (0.31)	0.12 (0.12)	RoboQoL CRF
		Missing (N%)	55 (34.6)	13 (21.7)	
Ambulance See and treat		Mean (SD)	0 (0)	0 (0)	RoboQoL CRF
		Missing (N%)	55 (34.6)	13 (21.7)	
Ambulance see and convey		Mean (SD)	0 (0)	0 (0)	RoboQoL CRF
		Missing (N%)	55 (34.6)	13 (21.7)	
A&E		Mean (SD)	0 (0)	0.02 (0.13)	RoboQoL CRF
		Missing (N%)	56 (35.2)	13 (21.7)	
Hospital Ward Day		Mean (SD)	0 (0)	0.02 (0.13)	RoboQoL CRF
		Missing (N%)	56 (35.2)	13 (21.7)	
Hospital Overnight Visit		Mean (SD)	0 (0)	0.05 (0.39)	RoboQoL CRF
		Missing (N%)	56 (35.2)	13 (21.7)	
Week 7					
**Primary Care**					
General Practitioner (GP)	Home Visit	Mean (SD)	0 (0)	0.03 (0.18)	RoboQoL CRF
		Missing (N%)	54 (34)	13 (21.7)	
	Surgery Visit	Mean (SD)	0.08 (0.3)	0.09 (0.28)	RoboQoL CRF
		Missing (N%)	55 (34.6)	15 (25.9)	
	Phone Appointment	Mean (SD)	0.05 (0.26)	0.02 (0.13)	RoboQoL CRF
		Missing (N%)	56 (35.2)	13 (21.7)	
Nurse	Home Visit	Mean (SD)	0 (0)	0.03 (0.18)	RoboQoL CRF
		Missing (N%)	56 (35.2)	13 (21.7)	

	Surgery Visit	Mean (SD)	0.01 (0.1)	0.05 (0.22)	RoboQoL CRF
		Missing (N%)	57 (35.8)	15 (25.9)	
	Phone Appointment	Mean (SD)	0.01 (0.1)	0.02 (0.13)	RoboQoL CRF
		Missing (N%)	57 (35.8)	13 (21.7)	
Occupational Therapist (OT)	Home Visit	Mean (SD)	0 (0)	0 (0)	RoboQoL CRF
		Missing (N%)	57 (35.8)	13 (21.7)	
	Surgery Visit	Mean (SD)	0 (0)	0 (0)	RoboQoL CRF
		Missing (N%)	58 (36.5)	13 (21.7)	
	Hospital Visit	Mean (SD)	0 (0)	0 (0)	RoboQoL CRF
		Missing (N%)	58 (36.5)	13 (21.7)	
Physiotherapist	Home Visit	Mean (SD)	0 (0)	0 (0)	RoboQoL CRF
		Missing (N%)	55 (34.6)	13 (21.7)	
	Surgery Visit	Mean (SD)	55 (0)	13 (0)	RoboQoL CRF
		Missing (N%)	55 (34.6)	13 (21.7)	
	Phone Appointment	Mean (SD)	0 (0)	0 (0)	RoboQoL CRF
		Missing (N%)	55 (34.6)	13 (21.7)	
	Hospital Visit	Mean (SD)	0 (0)	0 (0)	RoboQoL CRF
		Missing (N%)	55 (34.6)	13 (21.7)	
	Day Visit	Mean (SD)	0 (0)	0 (0)	RoboQoL CRF
		Missing (N%)	55 (34.6)	13 (21.7)	
**Secondary Care**					
Outpatient Appointment		Mean (SD)	0.5 (0.5)	0.28 (0.28)	RoboQoL CRF
		Missing (N%)	56 (35.2)	13 (21.7)	
Ambulance See and treat		Mean (SD)	0 (0)	0 (0)	RoboQoL CRF
		Missing (N%)	55 (34.6)	13 (21.7)	
Ambulance see and convey		Mean (SD)	0 (0)	0 (0)	RoboQoL CRF
		Missing (N%)	55 (34.6)	13 (21.7)	
A&E		Mean (SD)	0 (0)	0 (0)	RoboQoL CRF
		Missing (N%)	56 (35.2)	13 (21.7)	
Hospital Ward Day		Mean (SD)	0.03 (0.17)	0 (0)	RoboQoL CRF
		Missing (N%)	56 (35.2)	13 (21.7)	
Hospital Overnight Visit		Mean (SD)	0 (0)	0 (0)	RoboQoL CRF
		Missing (N%)	56 (35.2)	13 (21.7)	
Week 8					
**Primary Care**					
General Practitioner (GP)	Home Visit	Mean (SD)	0 (0)	0.03 (0.18)	RoboQoL CRF
		Missing (N%)	57 (35.8)	13 (21.7)	
	Surgery Visit	Mean (SD)	0.08 (0.3)	0.16 (0.37)	RoboQoL CRF
		Missing (N%)	57 (35.8)	15 (25.9)	
	Phone Appointment	Mean (SD)	0.01 (0.1)	0.02 (0.13)	RoboQoL CRF
		Missing (N%)	58 (36.5)	13 (21.7)	
Nurse	Home Visit	Mean (SD)	0 (0)	0.02 (0.13)	RoboQoL CRF
		Missing (N%)	55 (34.6)	14 (23.7)	
	Surgery Visit	Mean (SD)	0.03 (0.17)	0.1 (0.31)	RoboQoL CRF
		Missing (N%)	57 (35.8)	15 (25.9)	

	Phone Appointment	Mean (SD)	0.02 (0.14)	0 (0)	RoboQoL CRF
		Missing (N%)	55 (34.6)	14 (23.7)	
Occupational Therapist (OT)	Home Visit	Mean (SD)	0 (0)	0 (0)	RoboQoL CRF
		Missing (N%)	56 (35.2)	14 (23.7)	
	Surgery Visit	Mean (SD)	0 (0)	0 (0)	RoboQoL CRF
		Missing (N%)	56 (35.2)	14 (23.7)	
	Hospital Visit	Mean (SD)	0 (0)	0 (0)	RoboQoL CRF
		Missing (N%)	56 (35.2)	14 (23.7)	
Physiotherapist	Home Visit	Mean (SD)	0 (0)	0 (0)	RoboQoL CRF
		Missing (N%)	57 (35.8)	14 (23.7)	
	Surgery Visit	Mean (SD)	57 (0)	14 (0)	RoboQoL CRF
		Missing (N%)	57 (35.8)	14 (23.7)	
	Phone Appointment	Mean (SD)	0 (0)	0 (0)	RoboQoL CRF
		Missing (N%)	57 (35.8)	14 (23.7)	
	Hospital Visit	Mean (SD)	0 (0)	0 (0)	RoboQoL CRF
		Missing (N%)	57 (35.8)	14 (23.7)	
	Day Visit	Mean (SD)	0 (0)	0 (0)	RoboQoL CRF
		Missing (N%)	57 (35.8)	14 (23.7)	
**Secondary Care**					
Outpatient Appointment		Mean (SD)	0.55 (0.55)	0.2 (0.2)	RoboQoL CRF
		Missing (N%)	56 (35.2)	13 (21.7)	
Ambulance See and treat		Mean (SD)	0 (0)	0 (0)	RoboQoL CRF
		Missing (N%)	57 (35.8)	14 (23.7)	
Ambulance see and convey		Mean (SD)	0 (0)	0 (0)	RoboQoL CRF
		Missing (N%)	57 (35.8)	14 (23.7)	
A&E		Mean (SD)	0 (0)	0 (0)	RoboQoL CRF
		Missing (N%)	57 (35.8)	14 (23.7)	
Hospital Ward Day		Mean (SD)	0.01 (0.1)	0 (0)	RoboQoL CRF
		Missing (N%)	57 (35.8)	14 (23.7)	
Hospital Overnight Visit		Mean (SD)	0.02 (0.2)	0 (0)	RoboQoL CRF
		Missing (N%)	57 (35.8)	14 (23.7)	
Week 9					
**Primary Care**					
General Practitioner (GP)	Home Visit	Mean (SD)	0 (0)	0 (0)	RoboQoL CRF
		Missing (N%)	55 (34.6)	13 (21.7)	
	Surgery Visit	Mean (SD)	0.08 (0.27)	0.1 (0.3)	RoboQoL CRF
		Missing (N%)	56 (35.2)	13 (21.7)	
	Phone Appointment	Mean (SD)	0.02 (0.14)	0 (0)	RoboQoL CRF
		Missing (N%)	55 (34.6)	13 (21.7)	
Nurse	Home Visit	Mean (SD)	0.01 (0.1)	0 (0)	RoboQoL CRF
		Missing (N%)	55 (34.6)	13 (21.7)	
	Surgery Visit	Mean (SD)	0.02 (0.14)	0.07 (0.25)	RoboQoL CRF
		Missing (N%)	55 (34.6)	13 (21.7)	
	Phone Appointment	Mean (SD)	0.01 (0.1)	0 (0)	RoboQoL CRF
		Missing (N%)	55 (34.6)	13 (21.7)	

Occupational Therapist (OT)	Home Visit	Mean (SD)	0 (0)	0 (0)	RoboQoL CRF
		Missing (N%)	56 (35.2)	13 (21.7)	
	Surgery Visit	Mean (SD)	0 (0)	0 (0)	RoboQoL CRF
		Missing (N%)	56 (35.2)	13 (21.7)	
	Hospital Visit	Mean (SD)	0 (0)	0 (0)	RoboQoL CRF
		Missing (N%)	56 (35.2)	13 (21.7)	
Physiotherapist	Home Visit	Mean (SD)	0 (0)	0 (0)	RoboQoL CRF
		Missing (N%)	56 (35.2)	13 (21.7)	
	Surgery Visit	Mean (SD)	56 (0)	13 (0)	RoboQoL CRF
		Missing (N%)	56 (35.2)	13 (21.7)	
	Phone Appointment	Mean (SD)	0 (0)	0 (0)	RoboQoL CRF
		Missing (N%)	56 (35.2)	13 (21.7)	
	Hospital Visit	Mean (SD)	0 (0)	0 (0)	RoboQoL CRF
		Missing (N%)	56 (35.2)	13 (21.7)	
	Day Visit	Mean (SD)	0 (0)	0 (0)	RoboQoL CRF
		Missing (N%)	56 (35.2)	13 (21.7)	
**Secondary Care**					
Outpatient Appointment		Mean (SD)	0.51 (0.51)	0.33 (0.33)	RoboQoL CRF
		Missing (N%)	57 (35.8)	13 (21.7)	
Ambulance See and treat		Mean (SD)	0 (0)	0 (0)	RoboQoL CRF
		Missing (N%)	56 (35.2)	14 (23.7)	
Ambulance see and convey		Mean (SD)	0 (0)	0 (0)	RoboQoL CRF
		Missing (N%)	56 (35.2)	14 (23.7)	
A&E		Mean (SD)	0 (0)	0 (0)	RoboQoL CRF
		Missing (N%)	56 (35.2)	13 (21.7)	
Hospital Ward Day		Mean (SD)	0 (0)	0.08 (0.65)	RoboQoL CRF
		Missing (N%)	56 (35.2)	13 (21.7)	
Hospital Overnight Visit		Mean (SD)	0.01 (0.1)	0.02 (0.13)	RoboQoL CRF
		Missing (N%)	56 (35.2)	13 (21.7)	
Week 10					
**Primary Care**					
General Practitioner (GP)	Home Visit	Mean (SD)	0 (0)	0 (0)	RoboQoL CRF
		Missing (N%)	56 (35.2)	13 (21.7)	
	Surgery Visit	Mean (SD)	0.06 (0.24)	0.07 (0.25)	RoboQoL CRF
		Missing (N%)	56 (35.2)	13 (21.7)	
	Phone Appointment	Mean (SD)	0.03 (0.17)	0 (0)	RoboQoL CRF
		Missing (N%)	56 (35.2)	13 (21.7)	
Nurse	Home Visit	Mean (SD)	0 (0)	0 (0)	RoboQoL CRF
		Missing (N%)	57 (35.8)	13 (21.7)	
	Surgery Visit	Mean (SD)	0.02 (0.14)	0.08 (0.28)	RoboQoL CRF
		Missing (N%)	57 (35.8)	13 (21.7)	
	Phone Appointment	Mean (SD)	0.03 (0.17)	0.02 (0.13)	RoboQoL CRF
		Missing (N%)	57 (35.8)	13 (21.7)	
Occupational Therapist (OT)	Home Visit	Mean (SD)	0 (0)	0 (0)	RoboQoL CRF
		Missing (N%)	57 (35.8)	13 (21.7)	

	Surgery Visit	Mean (SD)	0 (0)	0 (0)	RoboQoL CRF
		Missing (N%)	57 (35.8)	13 (21.7)	
	Hospital Visit	Mean (SD)	0 (0)	0 (0)	RoboQoL CRF
		Missing (N%)	57 (35.8)	13 (21.7)	
Physiotherapist	Home Visit	Mean (SD)	0 (0)	0.03 (0.26)	RoboQoL CRF
		Missing (N%)	57 (35.8)	13 (21.7)	
	Surgery Visit	Mean (SD)	57 (0)	13 (0)	RoboQoL CRF
		Missing (N%)	57 (35.8)	13 (21.7)	
	Phone Appointment	Mean (SD)	0.04 (0.31)	0 (0)	RoboQoL CRF
		Missing (N%)	57 (35.8)	13 (21.7)	
	Hospital Visit	Mean (SD)	0 (0)	0 (0)	RoboQoL CRF
		Missing (N%)	57 (35.8)	13 (21.7)	
	Day Visit	Mean (SD)	0 (0)	0 (0)	RoboQoL CRF
		Missing (N%)	57 (35.8)	13 (21.7)	
**Secondary Care**					
Outpatient Appointment		Mean (SD)	0.58 (0.58)	0.12 (0.12)	RoboQoL CRF
		Missing (N%)	58 (36.5)	13 (21.7)	
Ambulance See and treat		Mean (SD)	0 (0)	0.02 (0.13)	RoboQoL CRF
		Missing (N%)	57 (35.8)	13 (21.7)	
Ambulance see and convey		Mean (SD)	0 (0)	0.02 (0.13)	RoboQoL CRF
		Missing (N%)	57 (35.8)	13 (21.7)	
A&E		Mean (SD)	0.01 (0.1)	0.02 (0.13)	RoboQoL CRF
		Missing (N%)	57 (35.8)	14 (23.7)	
Hospital Ward Day		Mean (SD)	0.03 (0.17)	0.1 (0.66)	RoboQoL CRF
		Missing (N%)	57 (35.8)	13 (21.7)	
Hospital Overnight Visit		Mean (SD)	0 (0)	0.05 (0.39)	RoboQoL CRF
		Missing (N%)	57 (35.8)	13 (21.7)	
Week 11					
**Primary Care**					
General Practitioner (GP)	Home Visit	Mean (SD)	0 (0)	0 (0)	RoboQoL CRF
		Missing (N%)	56 (35.2)	13 (21.7)	
	Surgery Visit	Mean (SD)	0.06 (0.27)	0.1 (0.3)	RoboQoL CRF
		Missing (N%)	56 (35.2)	13 (21.7)	
	Phone Appointment	Mean (SD)	0.04 (0.2)	0.03 (0.18)	RoboQoL CRF
		Missing (N%)	57 (35.8)	13 (21.7)	
Nurse	Home Visit	Mean (SD)	0 (0)	0 (0)	RoboQoL CRF
		Missing (N%)	57 (35.8)	14 (23.7)	
	Surgery Visit	Mean (SD)	0.04 (0.24)	0.05 (0.22)	RoboQoL CRF
		Missing (N%)	57 (35.8)	14 (23.7)	
	Phone Appointment	Mean (SD)	0.03 (0.22)	0 (0)	RoboQoL CRF
		Missing (N%)	57 (35.8)	15 (25.9)	
Occupational Therapist (OT)	Home Visit	Mean (SD)	0 (0)	0 (0)	RoboQoL CRF
		Missing (N%)	56 (35.2)	14 (23.7)	
	Surgery Visit	Mean (SD)	0 (0)	0 (0)	RoboQoL CRF
		Missing (N%)	56 (35.2)	14 (23.7)	

	Hospital Visit	Mean (SD)	0 (0)	0 (0)	RoboQoL CRF
		Missing (N%)	56 (35.2)	14 (23.7)	
Physiotherapist	Home Visit	Mean (SD)	0 (0)	0 (0)	RoboQoL CRF
		Missing (N%)	56 (35.2)	14 (23.7)	
	Surgery Visit	Mean (SD)	56 (0)	14 (0)	RoboQoL CRF
		Missing (N%)	56 (35.2)	14 (23.7)	
	Phone Appointment	Mean (SD)	0 (0)	0 (0)	RoboQoL CRF
		Missing (N%)	56 (35.2)	14 (23.7)	
	Hospital Visit	Mean (SD)	0 (0)	0 (0)	RoboQoL CRF
		Missing (N%)	56 (35.2)	14 (23.7)	
	Day Visit	Mean (SD)	0 (0)	0 (0)	RoboQoL CRF
		Missing (N%)	56 (35.2)	15 (25.9)	
**Secondary Care**					
Outpatient Appointment		Mean (SD)	0.58 (0.58)	0.22 (0.22)	RoboQoL CRF
		Missing (N%)	58 (36.5)	14 (23.7)	
Ambulance See and treat		Mean (SD)	0.01 (0.1)	0.02 (0.13)	RoboQoL CRF
		Missing (N%)	56 (35.2)	14 (23.7)	
Ambulance see and convey		Mean (SD)	0.01 (0.1)	0.02 (0.13)	RoboQoL CRF
		Missing (N%)	56 (35.2)	14 (23.7)	
A&E		Mean (SD)	0.03 (0.22)	0.05 (0.39)	RoboQoL CRF
		Missing (N%)	57 (35.8)	14 (23.7)	
Hospital Ward Day		Mean (SD)	0 (0)	0.02 (0.13)	RoboQoL CRF
		Missing (N%)	57 (35.8)	14 (23.7)	
Hospital Overnight Visit		Mean (SD)	0 (0)	0.02 (0.13)	RoboQoL CRF
		Missing (N%)	57 (35.8)	14 (23.7)	
Week 12					
**Primary Care**					
General Practitioner (GP)	Home Visit	Mean (SD)	0.01 (0.1)	0 (0)	RoboQoL CRF
		Missing (N%)	51 (32.1)	15 (25.9)	
	Surgery Visit	Mean (SD)	0.06 (0.28)	0.05 (0.22)	RoboQoL CRF
		Missing (N%)	51 (32.1)	15 (25.9)	
	Phone Appointment	Mean (SD)	0.06 (0.31)	0.04 (0.19)	RoboQoL CRF
		Missing (N%)	51 (32.1)	16 (28.1)	
Nurse	Home Visit	Mean (SD)	0 (0)	0 (0)	RoboQoL CRF
		Missing (N%)	52 (32.7)	15 (25.9)	
	Surgery Visit	Mean (SD)	0.04 (0.23)	0.07 (0.26)	RoboQoL CRF
		Missing (N%)	52 (32.7)	15 (25.9)	
	Phone Appointment	Mean (SD)	0 (0)	0 (0)	RoboQoL CRF
		Missing (N%)	52 (32.7)	15 (25.9)	
Occupational Therapist (OT)	Home Visit	Mean (SD)	0 (0)	0 (0)	RoboQoL CRF
		Missing (N%)	53 (33.3)	15 (25.9)	
	Surgery Visit	Mean (SD)	0 (0)	0 (0)	RoboQoL CRF
		Missing (N%)	54 (34)	15 (25.9)	
	Hospital Visit	Mean (SD)	0 (0)	0 (0)	RoboQoL CRF
		Missing (N%)	54 (34)	15 (25.9)	

Physiotherapist	Home Visit	Mean (SD)	0 (0)	0.02 (0.13)	RoboQoL CRF
		Missing (N%)	53 (33.3)	15 (25.9)	
	Surgery Visit	Mean (SD)	53 (0)	15 (0)	RoboQoL CRF
		Missing (N%)	53 (33.3)	15 (25.9)	
	Phone Appointment	Mean (SD)	0 (0)	0 (0)	RoboQoL CRF
		Missing (N%)	53 (33.3)	15 (25.9)	
	Hospital Visit	Mean (SD)	0 (0)	0 (0)	RoboQoL CRF
		Missing (N%)	53 (33.3)	15 (25.9)	
	Day Visit	Mean (SD)	0 (0)	0 (0)	RoboQoL CRF
		Missing (N%)	53 (33.3)	15 (25.9)	
**Secondary Care**					
Outpatient Appointment		Mean (SD)	0.7 (0.7)	0.29 (0.29)	RoboQoL CRF
		Missing (N%)	54 (34)	15 (25.9)	
Ambulance See and treat		Mean (SD)	0 (0)	0 (0)	RoboQoL CRF
		Missing (N%)	53 (33.3)	15 (25.9)	
Ambulance see and convey		Mean (SD)	0 (0)	0 (0)	RoboQoL CRF
		Missing (N%)	53 (33.3)	15 (25.9)	
A&E		Mean (SD)	0 (0)	0 (0)	RoboQoL CRF
		Missing (N%)	53 (33.3)	15 (25.9)	
Hospital Ward Day		Mean (SD)	0.08 (0.62)	0 (0)	RoboQoL CRF
		Missing (N%)	53 (33.3)	15 (25.9)	
Hospital Overnight Visit		Mean (SD)	0 (0)	0 (0)	RoboQoL CRF
		Missing (N%)	53 (33.3)	15 (25.9)	
