# Supplementary material for: Robotic and laparoscopic gynaecological surgery: a prospective multicentre observational cohort study and economic evaluation in England
Source: BMJ Open. 2023 Sep 28;13(9):e073990. doi: 10.1136/bmjopen-2023-073990 (PMC10546163; doi:10.1136/bmjopen-2023-073990)
Supplement: Supplementary data [file bmjopen-2023-073990supp002.pdf]

Supplemental Material S2: unit costs

Table S2.1 Unit costs of resources used during the surgical procedure including length of stay

Resource or unit intervention	Laparoscopic	Robotic	Cost per unit (£)	Cost Source
**Fixed costs of overheads**				
Operating theatre	Yes	Yes	250.00	South Tees Hospitals NHS Foundation based on personal communication with Alison Waines between Jan-Nov 2020
**Staff**				
**Resource or unit intervention**	**Laparoscopic**	**Robotic**	**Cost per unit (£)**	**Cost Source**
Consultant surgeon	Yes	Yes	109.00	PSSRU 2018/2019 based on "Consultant Surgical"
Assistant surgeon (registrar)	Yes	Yes	47.00	PSSRU 2018/2019 based on "Registrar"
Consultant anaesthetist	Yes	Yes	109.00	PSSRU 2018/2019 based on "Consultant medical"
Anaesthetist registrar	Yes	Yes	47.00	PSSRU 2018/2019 based on "Registrar"
Anaesthetist nurse	Yes	Yes	38.00	PSSRU 2018/2019 based on "hospital based nurses" (Band 5)
Nurse	Yes	Yes	38.00	PSSRU 2018/2019 based on "hospital based nurses" (Band 5)
Healthcare assistant	Yes	Yes	8.93	NHS Employers website 2018/2019 hourly rate (Band 2 with 3-4 years of experience)
Recovery practitioner	Yes	Yes	38.00	PSSRU 2018/2019 based on "hospital based nurses" (Band 5)
**Capital equipment**				
			**Cost (£)**	**Cost Source**

**Resource or unit intervention**	**Laparoscopic**	**Robotic**	**Capital (life expectancy)**	**Annualised cost**	**Per case**	South Tees Hospitals NHS Foundation based on personal communication with Alison Waines between Jan-Nov 2020
Laparoscopic device (screens included)	Yes	n/a	90 000.00 (8 years)	1 ,092.90	£17. 25	KARL STORZ Endoscopy (UK) Ltd, based on personal communication with Matthew Taylor (Endoscopic sales specialist) in August 2020
Robotic device	n/a	Yes	1 000 300.00 (8 years)	145 520.29	191. 73	South Tees Hospitals NHS Foundation based on personal communication with Jennifer Donnelly between Jan-Nov 2020
Robotic device maintenance cost	n/a	Yes	100 000.00 (per annum)	395.26	131. 75	South Tees Hospitals NHS Foundation based on personal communication with Jeremy Twigg between Jan-Nov 2020
Diathermy console	Yes	Yes	10 800.75 (10 years)	1 298.70	1.71	South Tees Hospitals NHS Foundation based on personal communication with Jeremy Twigg between Jan-Nov 2020
Anaesthetic machine	Yes	Yes	20 000.00 (8 years)	2 909.53	3.83	South Tees Hospitals NHS Foundation based on personal communication with Jennifer Donnelly between Jan-Nov 2020
PCA pump	Yes	Yes	5 000.00 (5 years)	1 107.41	1.46	South Tees Hospitals NHS Foundation based on personal communication with Jennifer Donnelly between Jan-Nov 2020
Suction machine	Yes	Yes	2 000 (5 years)	442.96	0.58	South Tees Hospitals NHS Foundation based on personal communication with Jennifer Donnelly between Jan-Nov 2020
All annualised costs above have been annualised based on calculated annualisation factors, days worked per year is assumed as 253 days and equipment used on average three times per day.						
**Consumables Surgical equipment specific to laparoscopic and robotic surgery**						
**Resource or unit intervention**	**Laparoscopic**	**Robotic**	**Cost per unit (£)**			**Cost Source**
Gel port/trocar	Yes	n/a	26.00			South Tees Hospitals NHS Foundation based on personal communication with Alison Waines between Jan-Nov 2020
55mm dual port/trocar	Yes	n/a	96.00			South Tees Hospitals NHS Foundation based on personal communication with Alison Waines between Jan-Nov 2020

11mm port/trocar	Yes		24.00	South Tees Hospitals NHS Foundation based on personal communication with Alison Waines between Jan-Nov 2020
Verris lap pack	Yes	n/a	5.00	South Tees Hospitals NHS Foundation based on personal communication with Alison Waines between Jan-Nov 2020
Vcare manipulator	Yes	1	50.00	South Tees Hospitals NHS Foundation based on personal communication with Alison Waines between Jan-Nov 2020
Applied scissors	Yes	n/a	20.00	South Tees Hospitals NHS Foundation based on personal communication with Alison Waines between Jan-Nov 2020
Harmonic (ethicon)	Yes	n/a	550.00	South Tees Hospitals NHS Foundation based on personal communication with Alison Waines between Jan-Nov 2020
Misc. forceps	Yes	n/a	100.00	South Tees Hospitals NHS Foundation based on personal communication with Alison Waines between Jan-Nov 2020
Vlock suture	Yes	Yes	25.00	South Tees Hospitals NHS Foundation based on personal communication with Alison Waines between Jan-Nov 2020
Endoclose	Yes	Yes	10.00	South Tees Hospitals NHS Foundation based on personal communication with Alison Waines between Jan-Nov 2020
Syringes	Yes	Yes	0.20	South Tees Hospitals NHS Foundation based on personal communication with Alison Waines between Jan-Nov 2020
Scissors (robot specific)	n/a	Yes	342.00	South Tees Hospitals NHS Foundation based on personal communication with Alison Waines between Jan-Nov 2020
Pro-grasp	n/a	Yes	234.96	South Tees Hospitals NHS Foundation based on personal communication with Alison Waines between Jan-Nov 2020
Maryland bi-polar	n/a	Yes	289.20	South Tees Hospitals NHS Foundation based on personal communication with Alison Waines between Jan-Nov 2020
Needle holder	n/a	Yes	234.96	South Tees Hospitals NHS Foundation based on personal

				communication with Alison Waines between Jan-Nov 2020
Scope sterilisation for 30 degrees & 0 degrees	n/a	Yes	220.00	South Tees Hospitals NHS Foundation based on personal communication with Alison Waines between Jan-Nov 2020
Accessory set sterilisation	n/a	Yes	100.00	South Tees Hospitals NHS Foundation based on personal communication with Alison Waines between Jan-Nov 2020
**General consumable surgical equipment**				
**Resource or unit intervention**	**Laparoscopic**	**Robotic**	**Cost per unit (£)**	**Cost Source**
Arterial line	Yes	Yes	45.00	South Tees Hospitals NHS Foundation based on personal communication with Alison Waines and Richard Bickle between Jan-Nov 2020
Central line	Yes	Yes	140.00	South Tees Hospitals NHS Foundation based on personal communication with Alison Waines and Richard Bickle between Jan-Nov 2020
12F catheter	Yes	Yes	3.00	South Tees Hospitals NHS Foundation based on personal communication with Alison Waines and Richard Bickle between Jan-Nov 2020
Urine drainage bag	Yes	Yes	1.00	South Tees Hospitals NHS Foundation based on personal communication with Alison Waines and Richard Bickle between Jan-Nov 2020
Monocryl	Yes	Yes	48.00	South Tees Hospitals NHS Foundation based on personal communication with Alison Waines and Richard Bickle between Jan-Nov 2020
Bair Hugger warmer	Yes	Yes	6.00	South Tees Hospitals NHS Foundation based on personal communication with Alison Waines and Richard Bickle between Jan-Nov 2020
Fluid warmer	Yes	Yes	4.00	South Tees Hospitals NHS Foundation based on personal communication with Alison

				Waines and Richard Bickle between Jan-Nov 2020
Drape	1	n/a	22.30	South Tees Hospitals NHS Foundation based on personal communication with Alison Waines between Jan-Nov 2020
4 arm drape	n/a	Yes	276.00	South Tees Hospitals NHS Foundation based on personal communication with Alison Waines between Jan-Nov 2020
Scissor tip cover	n/a	1	18.15	South Tees Hospitals NHS Foundation based on personal communication with Alison Waines between Jan-Nov 2020
		**Blood products**		
**Resource or unit intervention**	**Laparoscopic**	**Robotic**	**Cost per unit (£)**	**Cost Source**
Standard red blood cells	Yes	n/a	128.99	NHS Blood and Transplant price 2018/19
		**Other resources**		
**Resource or unit intervention**	**Laparoscopic**	**Robotic**	**Cost per unit (£)**	**Cost Source**
Anaesthetic equipment	Yes	Yes	18.00	South Tees Hospitals NHS Foundation based on personal communication with Richard Bickle between Jan-Nov 2020
Anaesthetic drugs	Yes	Yes	47.00	South Tees Hospitals NHS Foundation based on personal communication with Richard Bickle between Jan-Nov 2020
IV fluids	Yes	Yes	5.50	South Tees Hospitals NHS Foundation based on personal communication with Richard Bickle between Jan-Nov 2020
		**Type of stay**		
ICU	Yes	Yes	1 417.63	Fromhttps://www.nice.org.uk/guidance/GID-NG10072/documents/evidence-review-12inflated using PSSRU
Ward day	Yes	Yes	£416.90	From https://www.nice.org.uk/guidance/GID-NG10072/documents/evidence-

				review-12, inflated using PSSRU index

Table S2.2 Unit costs for NHS resources used during follow-up

Resource	Cost (£)	Source
GP Home	100.62	From Unit Costs of Health and Social Care 2019, a GP costs £4.30 per minute.<br>Unit Costs of Health and Social Care 2015, the average home visit is 11.4 minutes with 12 minutes of travel time.
GP Surgery	39	Costs from Unit Costs of Health and Social Care 2019
GP Phone	15.32	Costs from Unit Costs of Health and Social Care 2019
Nurse Home	16.38	Costs from Unit Costs of Health and Social Care 2019<br>We assumed the travel and contact time was the same as GP home visit.
Nurse Surgery	10.85	Costs from Unit Costs of Health and Social Care 2019<br>From Unit Costs of Health and Social Care 2015, the average surgery contact time was 15.5 minutes.
Nurse Phone	7.8	Cost from Unit Costs of Health and Social Care 2019
OT Home	46.4	Costs from Unit Costs of Health and Social Care 2019<br>From Unit Costs of Health and Social Care 2010 , an average OT session is 40 minutes.<br>We assumed the travel time was the same as a GP.
OT Surgery	32	Costs from Unit Costs of Health and Social Care 2019<br>From Unit Costs of Health and Social Care 2010 , an average OT session is 40 minutes.
OT Hospital	70	Costs from Unit Costs of Health and Social Care 2019
Physio Home	34.23	Costs from Unit Costs of Health and Social Care 2019, we assumed the average between bands 5 and 6.<br>We assumed same length appointment as OT (40 minutes).<br>We assumed the same travel time as GP.
Physio Surgery	26.33	Costs from Unit Costs of Health and Social Care 2019, we assumed the average between bands 5 and 6.<br>We assumed same length appointment as OT (40 minutes).
Physio Phone	4.56	Costs from Unit Costs of Health and Social Care 2019, we assumed the average between bands 5 and 6.<br>From Unit Costs of Health and Social Care 2019, we assumed same length phone call as a nurse.
Physio Hospital	58	Costs from Unit Costs of Health and Social Care 2019.
Physio Day Ward	58	Cost from Unit Costs of Health and Social Care 2019.
Outpatient	135	Cost from Unit Costs of Health and Social Care 2019.
Emergency Ambulance	196	Cost from Unit Costs of Health and Social Care 2019. We assumed the costs for "See and treat and refer"
Emergency Ambulance Hospital	258	Cost from Unit Costs of Health and Social Care 2019. We assumed the costs for "See and treat and convey"

Hospital A&E	135.8	Costs from NHS Reference costs 2018/2019, we assumed Accident and Emergency
Hospital Day Ward	416.9	Cost from https://www.nice.org.uk/guidance/GID-NG10072/documents/evidence-review-12.<br>This was inflated to 2019 prices using Unit Costs of Health and Social Care 2019.
Hospital Overnight ward	416.9	Cost from https://www.nice.org.uk/guidance/GID-NG10072/documents/evidence-review-12.<br>This was inflated to 2019 prices using Unit Costs of Health and Social Care 2019.
**Serious Adverse Events**		
Resource	Unit Cost (£)	Source
Sepsis	2 205	Weighted average of NHS tariff HRG codes: WJ06A, WJ06B, WJ06C, WJ06D, WJ06E, WJ06F, WJ06G, WJ06H, WJ06J
Bowel obstruction	3 340	Weighted average of NHS tariff HRG codes: FF61A, FF61B, FF61C
Embolism/CVA	3 334	Weighted average of NHS tariff HRG codes: AA35A, AA35B, AA35C, AA35D, AA35E, AA35F
