# Supplementary figures and images for: Robotic and laparoscopic gynaecological surgery: a prospective multicentre observational cohort study and economic evaluation in England
Source: BMJ Open. 2023 Sep 28;13(9):e073990. doi: 10.1136/bmjopen-2023-073990 (PMC10546163; doi:10.1136/bmjopen-2023-073990)

Supplemental material S3: Patient flow diagram

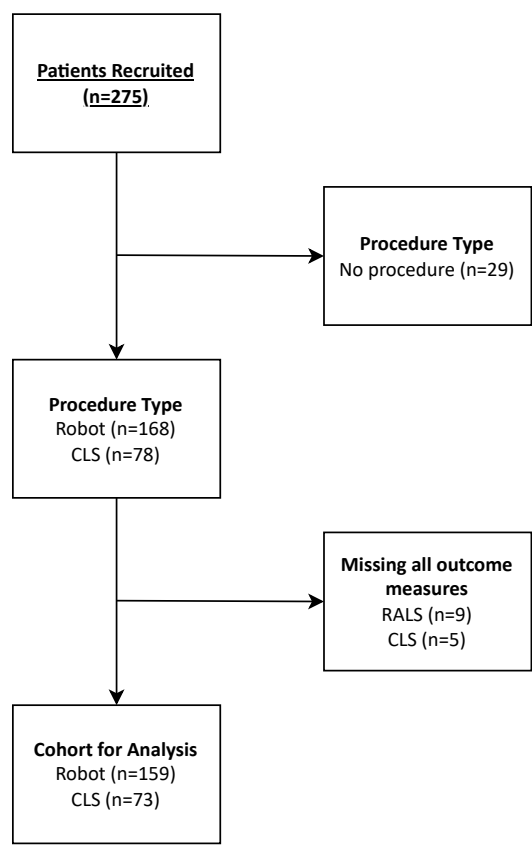

Supplement: Supplementary data [file bmjopen-2023-073990supp003.pdf]
