# Supplementary material for: Robotic and laparoscopic gynaecological surgery: a prospective multicentre observational cohort study and economic evaluation in England
Source: BMJ Open. 2023 Sep 28;13(9):e073990. doi: 10.1136/bmjopen-2023-073990 (PMC10546163; doi:10.1136/bmjopen-2023-073990)
Supplement: Supplementary data [file bmjopen-2023-073990supp004.pdf]

Supplemental Material S4

Table S4.1 list of serious adverse events

**Serious Adverse event**	**Event time**	**RALS (n=159)**	**CLS (n=73)**
Abdominal infection	Post-procedure	0	1
Accidental dislodgement of a needle	Post-procedure	0	1
CVA and DVT	Post-procedure	1	0
Haematoma with active bleeding	Post-procedure	1	0
lower suprapubic abdominal pain	Post-procedure	1	0
Nausea and vomiting	Post-procedure	2	0
Nervous system disorders	Post-procedure	1	0
Vaginal prolapse	Post-procedure	0	1
Sepsis	Week-1	0	2
Bowel obstruction	Week-2	0	1
CVA	Week-2	1	0
