# Supplementary material for: Robotic and laparoscopic gynaecological surgery: a prospective multicentre observational cohort study and economic evaluation in England
Source: BMJ Open. 2023 Sep 28;13(9):e073990. doi: 10.1136/bmjopen-2023-073990 (PMC10546163; doi:10.1136/bmjopen-2023-073990)
Supplement: Supplementary data [file bmjopen-2023-073990supp005.pdf]

Supplemental material S5

S5.1: Procedure and post-procedure to discharge cost summaries for CLS and RALS patients.

Cost component	RALS (n=159)	CLS (n=73)	P
**Procedure Costs**			
**Staff cost**			**0.231**
Mean ± SD	1 851 (688)	1 703 (940)	
Median (IQR)	1 733 (1 068 to 2 278)	1 522 (987 to 2 117)	
**Theatre cost**			**0.231**
Mean ± SD	765 (284)	704 (388)	
Median (IQR)	716 (537 to 941)	630 (408 to 875)	
**Blood Products Cost**			**0.848**
Mean ± SD	7 (42)	9 (62)	
Median (IQR)	0 (0 to 0)	0 (0 to 0)	
**Equipment cost***	2 134	1 325	
**Total Procedure Cost**			**<0.001**
Mean ± SD	4 758 (972)	3 741 (1 350)	
Median (IQR)	4 585 (3 972 to 5 356)	3 477 (2 722 to 4 317)	
**Length of stay cost**			**0.352**
Mean ± SD	704 (1 002)	851 (1 159)	
Median (IQR)	416 (416 to 416)	416 (416 to 833)	**0.002**
**Total cost**			
Mean ± SD	5 462 (1 472)	4 592 (2 072)	
Median (IQR)	5 126 (4 404 to 6 142)	3 905 (3 195 to 5 162)	

All data are presented as mean ± (SD) or median (interquartile range (IQR)) unless otherwise stated. All numbers of costs in £ Sterling.
*Equipment costs are from what are used in a ‘typical’ procedure and are assumed a fixed cost across all patients in the same cohort.
SD standard deviation, IQR interquartile range, CLS – conventional laparoscopic surgery, RALS robotically assisted laparoscopic surgery.

S5.2 Summary information for follow-up costs of RALS and CLS patients

Cost component	RALS (n=159)	CLS (n=73)	P
**Week 1**			**0.092**
Mean ± SD	125 ± 333	336 ± 996	
Median (IQR)	0 (0 to 115)	0 (0 to 143)	
Missing	45	9	
**Week 2**			**0.188**
Mean ± SD	94 ± 300	246 ± 907	
Median (IQR)	0 (0 to 135)	0 (0 to 15)	
Missing	45	7	

**Week 3**			**0.578**
Mean ± SD	69 ± 135	103 ± 496	
Median (IQR)	0 (0 to 135)	0 (0 to 39)	
Missing	47	11	
**Week 4**			**0.084**
Mean ± SD	55 ± 87	30 ± 81	
Median (IQR)	0 (0 to 135)	0 (0 to 11)	
Missing	52	10	
**Week 5**			**0.748**
Mean ± SD	40 ± 71	33 ± 99	
Median (IQR)	0 (0 to 39)	0 (0 to 11)	
Missing	61	15	
**Week 6**			**0.268**
Mean ± SD	49 ± 95	29 ± 99	
Median (IQR)	0 (0 to 54)	0 (0 to 135)	
Missing	56	16	
**Week 7**			**0.187**
Mean ± SD	80 ± 145	44 ± 153	
Median (IQR)	0 (0 to 135)	0 (0 to 11)	
Missing	61	15	
**Week 8**			**0.023**
Mean ± SD	88 ± 186	31 ± 101	
Median (IQR)	0 (0 to 50)	0 (0 to 11)	
Missing	62	16	
**Week 9**			**0.784**
Mean ± SD	77 ± 183	91 ± 380	
Median (IQR)	0 (0 to 15)	0 (0 to 39)	
Missing	58	14	
**Week 10**			**0.891**
Mean ± SD	96 ± 209	87 ± 329	
Median (IQR)	0 (0 to 39)	0 (0 to 11)	
Missing	58	14	
**Week 11**			**0.608**
Mean ± SD	84 ± 214	64 ± 164	
Median (IQR)	0 (0 to 15)	0 (0 to 15)	
Missing	61	15	
**Week 12**			**0.049**
Mean ± SD	116 ± 331	44 ± 110	
Median (IQR)	0 (0 to 39)	0 (0 to 26)	
Missing	55	16	
