# Supplementary material for: Robotic and laparoscopic gynaecological surgery: a prospective multicentre observational cohort study and economic evaluation in England
Source: BMJ Open. 2023 Sep 28;13(9):e073990. doi: 10.1136/bmjopen-2023-073990 (PMC10546163; doi:10.1136/bmjopen-2023-073990)

Supplemental Material S6

Fig. S6.1: Graph A: Cost-effectiveness plane for adjusted sensitivity analysis assuming 1 procedure per day. Graph B: Willingness to pay for adjusted sensitivity analysis assuming 1 procedure per day

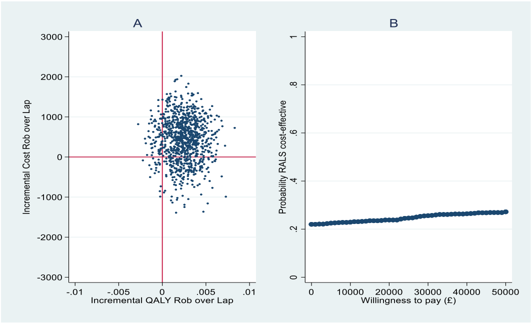

Fig. S6.2: Graph A: Cost-effectiveness plane for adjusted sensitivity analysis assuming 2 procedures per day. Graph B: Willingness to pay for adjusted sensitivity analysis assuming 2 procedures per day

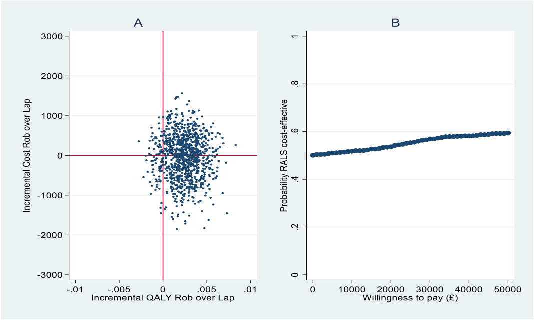

Fig. S6.3: Graph A: Cost-effectiveness plane for adjusted sensitivity analysis assuming 4 procedures per day. Graph B: Willingness to pay for adjusted sensitivity analysis assuming 4 procedures per day

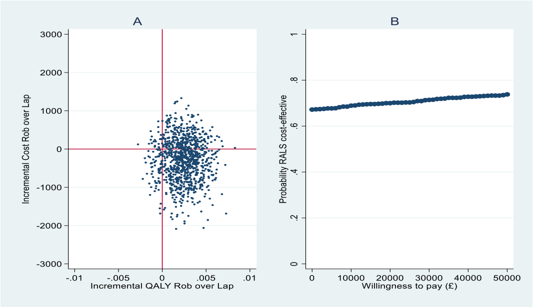

Supplement: Supplementary data [file bmjopen-2023-073990supp006.pdf]
