# Supplementary material for: Robotic and laparoscopic gynaecological surgery: a prospective multicentre observational cohort study and economic evaluation in England
Source: BMJ Open. 2023 Sep 28;13(9):e073990. doi: 10.1136/bmjopen-2023-073990 (PMC10546163; doi:10.1136/bmjopen-2023-073990)
Supplement: Author's manuscript [file bmjopen-2023-073990.draft_revisions.pdf]

BMJ Open is committed to open peer review. As part of this commitment we make the peer review history of every article we publish publicly available.

When an article is published we post the peer reviewers' comments and the authors' responses online. We also post the versions of the paper that were used during peer review. These are the versions that the peer review comments apply to.

The versions of the paper that follow are the versions that were submitted during the peer review process. They are not the versions of record or the final published versions. They should not be cited or distributed as the published version of this manuscript.

BMJ Open is an open access journal and the full, final, typeset and author-corrected version of record of the manuscript is available on our site with no access controls, subscription charges or pay-per-view fees (http://bmjopen.bmj.com).

If you have any questions on BMJ Open's open peer review process please email info.bmjopen@bmj.com

BMJ Open

Robotic and Laparoscopic Gynaecological Surgery: A Prospective Multi-Centre Observational Cohort Study and Economic Evaluation

Journal:	*BMJ Open*
Manuscript ID	bmjopen-2023-073990
Article Type:	Original research
Date Submitted by the Author:	30-Mar-2023
Complete List of Authors:	McCarthy, Andrew; Northumbria University, Department of Nursing, Midwifery and Health<br>Samarakoon, Dilupa; Northumbria University, Department of Nursing, Midwifery and Health<br>Gray, Joanne; Northumbria University,<br>Mcmeekin, Peter; Northumbria University, Faculty of Health and Life Sciences<br>McCarthy, Stephen; Northumbria University, Department of Nursing, Midwifery and Health<br>Newton, Claire; University Hospitals Bristol and Weston NHS Foundation Trust, Department of Gynaecology Oncology; University of Bristol<br>Nobbenhuis, Marielle; Royal Marsden NHS Foundation Trust, Department of Gynaecological Oncology<br>Lippiatt, Jonathan; University Hospitals Dorset NHS Foundation Trust, Department of Gynaecological Oncology<br>Twigg, Jeremy; Coventry and Warwickshire Hospital, Department of Gynaecological Oncology
Keywords:	Gynaecological oncology < GYNAECOLOGY, Minimally invasive surgery < GYNAECOLOGY, HEALTH ECONOMICS

SCHOLARONE™
Manuscripts

I, the Submitting Author has the right to grant and does grant on behalf of all authors of the Work (as defined in the below author licence), an exclusive licence and/or a non-exclusive licence for contributions from authors who are: i) UK Crown employees; ii) where BMJ has agreed a CC-BY licence shall apply, and/or iii) in accordance with the terms applicable for US Federal Government officers or employees acting as part of their official duties; on a worldwide, perpetual, irrevocable, royalty-free basis to BMJ Publishing Group Ltd ("BMJ") its licensees and where the relevant Journal is co-owned by BMJ to the co-owners of the Journal, to publish the Work in this journal and any other BMJ products and to exploit all rights, as set out in our licence.

The Submitting Author accepts and understands that any supply made under these terms is made by BMJ to the Submitting Author unless you are acting as an employee on behalf of your employer or a postgraduate student of an affiliated institution which is paying any applicable article publishing charge ("APC") for Open Access articles. Where the Submitting Author wishes to make the Work available on an Open Access basis (and intends to pay the relevant APC), the terms of reuse of such Open Access shall be governed by a Creative Commons licence – details of these licences and which Creative Commons licence will apply to this Work are set out in our licence referred to above.

Other than as permitted in any relevant BMJ Author's Self Archiving Policies, I confirm this Work has not been accepted for publication elsewhere, is not being considered for publication elsewhere and does not duplicate material already published. I confirm all authors consent to publication of this Work and authorise the granting of this licence.

For peer review only

Robotic and Laparoscopic Gynaecological Surgery: A Prospective Multi-Centre Observational Cohort Study and Economic Evaluation

Authors:

Andrew McCarthy[1*], Dilupa Samarakoon[1], Joanne Gray[1], Peter McMeekin[1], Stephen McCarthy[1], Claire Newton[2,3], Marielle Nobbenhuis[4], Jonathan Lippiatt[5], Jeremy Twigg[6]

Institutions:

1 Department of Nursing, Midwifery and Health, Northumbria University, Newcastle Upon Tyne, UK

2 Department of Gynaecology Oncology, St Michaels hospital, University Hospitals Bristol and Weston NHS foundation trust, UK.

3. University of Bristol, Beaconhouse, Queens Road, Bristol, BS8 1QU, UK

4 Department of Gynaecological Oncology, The Royal Marsden NHS Foundation Trust, Chelsea, London SW3 6JJ, UK

5 Department of Gynaecological Oncology, University Hospitals Dorset NHS Foundation Trust, Poole, UK

6 Department of Gynaecological Oncology, University Hospital of Coventry and Warwickshire, Coventry, UK

**Correspondence to:* Andrew McCarthy, Coach Lane Campus, Northumbria University, Newcastle upon Tyne, NE7 7XA, United Kingdom. Telephone: 0191 215 6718. Email: andrew2.mccarthy@northumbria.ac.uk

For peer review only

ABSTRACT

Objective

To compare the effectiveness and cost effectiveness of robot-assisted laparoscopic surgery (RALS) versus conventional "*straight stick*" laparoscopic surgery (CLS) in women undergoing hysterectomy as part of their treatment for either suspected or proven gynaecological malignancy.

Design

Multicentre prospective observational cohort study.

Setting

Patients aged 16+ undergoing hysterectomy as part of their treatment for gynaecological malignancy at 12 NHS cancer units and centres in England between August 2017 and February 2020.

Participants

298 patients recruited with 159 RALS, 73 CLS eligible for analysis.

Outcome measures

Primary outcome was the European Organisation for Research and Treatment of Cancer Quality of Life measure (EORTC). Secondary outcomes included EQ-5D-5L utility, six-minute walk test (6MWT), NHS costs using pounds Sterling (£) 2018-19 prices and cost-effectiveness. The cost-effectiveness evaluation compared EQ-5D-5L quality adjusted life years and costs between RALS and CLS.

Results

No difference was identified between RALS and CLS for EORTC, EQ-5D-5L utility, and 6MWT. RALS had an unadjusted mean cost difference of £556 (95%CI -£314 to £1 315) versus CLS and a mean quality adjusted life year (QALY) difference of 0.0024 (95%CI -0.00051 to 0.0057), non-parametric ICER of £152 843 per QALY. For the adjusted cost-effectiveness analysis, RALS dominated CLS with a

For peer review only

mean cost difference of £-188 (95%CI -£1 321 to £827) and QALY difference of 0.0024 (95%CI -0.0008 to 0.0057).

Conclusions

Findings suggest that RALS versus CLS in women undergoing hysterectomy (after adjusting for differences in morbidity) is cost effective with lower costs and QALYs. Results are highly sensitive to the usage of robotic hardware with higher usage increasing the probability of cost-effectiveness. A non-inferiority RCT would be of benefit to decision makers to provide further evidence on the cost-effectiveness of RALS versus CLS but may not be practical due to surgical preferences of surgeons and the extensive roll out of RALS.

Strengths and limitations of this study

- This study contains the first NHS England multi-center cost-effectiveness analysis comparing robotically assisted surgery (RALS) versus conventional laparoscopic surgery (CLS) for the treatment of gynaecological cancer.
- Cost estimates were derived from detailed micro-costing of RALS and CLS procedures with up to 12-weeks follow-up data post procedure. This will help inform future resource allocation and procurement decisions.
- This was a pragmatic multicentre comparative cohort study with regression methods used to account for differences in baseline characteristics between groups.
- A definitive RCT with embedded full economic evaluation would be beneficial. However, due to the extensive rollout of robotic surgery and surgeon preferences, an RCT would be difficult to conduct in practice.

INTRODUCTION

Background

Surgery used to treat early-stage gynaecological cancer has changed over the last 20 years through the introduction of minimal access surgical approaches. Surgeons have developed “keyhole”

For peer review only - http://bmjopen.bmj.com/site/about/guidelines.xhtml

For peer review only

(laparoscopic) techniques using “straight stick” surgical instruments. This technique is termed conventional laparoscopic surgery (CLS). A number of studies have randomized patients with gynaecological cancer to CLS or open surgery. The GOG LAP2 study showed that patients receiving CLS had an improved safety profile, less antibiotic use and shorter hospital stay as well as higher quality of life scores (better physical functioning, better body image, less pain), earlier resumption of normal activities and earlier return to work over the 6 weeks following surgery[1], compared to laparotomy in patients with endometrial cancer. A second trial (LACE study) showed quality of life (QoL) improvements and an adverse event profile that favoured CLS compared to OH (Open Hysterectomy)[2]. A Dutch study also confirmed that a laparoscopic approach provided benefit with respect to shorter hospital stay, less pain, and quicker resumption of daily activities compared to laparotomy[1]. CLS in gynaecological oncology has not been a panacea. The LAP2 and Dutch studies showed that despite the use of CLS, conversion rates varied from 10% - 26%, and this rate increased to 57% in women with a body mass index (BMI) greater than 40 kg/m2. Further, uptake of CLS in the UK has been slow and fragmented: for example, the ASTEC study (A Study in the Treatment of Endometrial Cancer, which recruited from 1998 to 2005) demonstrated that only 7% of patients being treated for endometrial cancer had a laparoscopic hysterectomy, whilst the remaining 93% had open surgery[3]. Whilst the adverse events were lower in the CLS group compared to the OH group in ASTEC, a third of CLS patients in this study had their procedure converted to open surgery.

The introduction of robot assisted laparoscopic surgery (RALS) has facilitated a greater number of surgeons being able to offer minimal access surgery to their patients[4]. The combination of a wristed instrument design and 3-D high definition optics of the robot allows the surgeon to dissect remove and reconstruct tissues in a way that has not been possible [5]. Outcomes for RALS appear favourable to open surgery and there are a considerable number of publications suggesting that standard hospital performance indicators are as good for RALS as they are for other surgical techniques[6-9]. However, RALS brings with it a greater cost than CLS: operative times are longer, and equipment

For peer review only - http://bmjopen.bmj.com/site/about/guidelines.xhtml

costs are greater for benign as well as oncological gynaecological procedures with no difference in key performance indicators[4 10]. A recent Cochrane review concluded "…we found limited evidence on the effectiveness and safety of RALS compared with CLS or open surgery for surgical procedures performed for gynaecological cancer; therefore, its use should be limited to clinical trials. Ongoing trials are likely to have an important impact on evidence related to the use of RALS in gynaecology…"[11].

As a consequence, health care commissioners and policy makers in the UK have made RALS an area of investigation so that informed decisions can be made about the routine commissioning of robotics. For now, NHS England have routinely commissioned RALS for the treatment of prostate cancer but for all other RALS services, robotic consumables are excluded from tariff[12 13]. Simultaneous to the debate around healthcare costs, health and social care policymakers have focused on patient-reported outcome measures (PROMs). These are increasingly recognised as important in healthcare resource allocation decisions both globally and in the NHS[14]. NHS England analysis of PROMs collected following surgical procedures enables "…providers, commissioners and other stakeholders to make informed changes to the delivery of their services…"[15]. This data feeds into key NHS Outcomes Framework Indicators and the evidence of performance in these domains impacts on funding decisions across the healthcare system[16] .

There is little research which compares health-related quality of life (HRQOL) between RALS and CLS. Whilst Herling et al provided evidence of a speedy recovery in many HRQOL domains following RALS, comparison of HRQOL recovery across different modes of surgery has not been explored[17]. Kumar et al attempted a comparison between open surgery and RALS, looking retrospectively at comparative HRQOL in endometrial cancer[18]. They found that RALS was associated with improved HRQOL compared with an open approach. The study however, did not compare RALS with CLS, and nor did it use validated HRQOL questionnaires or use recommended metrics for endometrial cancer[19].

For peer review only

For peer review only - http://bmjopen.bmj.com/site/about/guidelines.xhtml

Any future decisions on provision of RALS in gynaecological cancer should be determined by informed decision making of both the effectiveness and cost-effectiveness of this intervention compared to CLS. However, there is a paucity of data regarding comparative cost- effectiveness in this area.

Objectives

The aims of this study were to compare the effectiveness in terms of HRQoL, functional capacity, and cost effectiveness of robot-assisted laparoscopic surgery (RALS) versus conventional "*straight stick*" laparoscopic surgery (CLS) in women undergoing hysterectomy as part of their treatment for either suspected or proven gynaecological malignancy.

METHODS

Study design

This prospective comparative observational cohort study with embedded cost-effectiveness analysis recruited patients between August 2017 and February 2020 across 12 NHS Cancer Units and Centres in England. Patients referred for suspected gynaecological cancer or with a proven diagnosis of endometrial, cervical, and ovarian cancer were given a patient information sheet and asked for consent to be part of this study. Demographic and anthropometric data was collected at baseline alongside relevant outcome measures. and participants were followed up at 2-, 4- and 12-weeks post-procedure.

Participant inclusion criteria

Inclusion criteria consisted of patients aged over 16 years who were referred with diagnosis (or suspected diagnosis) of gynaecological malignancy and about to undergo surgery RALS or CLS. Patients were also required to be fit for surgery and have the ability to complete the six-minute walk

For peer review only - http://bmjopen.bmj.com/site/about/guidelines.xhtml

For peer review only

test (6MWT). They were also required to be having a hysterectomy as part of their surgical treatment to be eligible and be able to provide informed consent. Patients were excluded if surgery was only possible via the abdominal approach or if the midline laparotomy incision was anticipated to go beyond 4cm above the umbilicus. Other exclusion criteria were surgery for a large ovarian tumour and patients undergoing radical cytoreductive surgery. Patients were also excluded if they were unable to complete outcome measures due to cognitive or language impairments disabling them from completing the quality-of-life questionnaires or lacked capacity to give consent.

Surgical Procedures

Surgical procedures followed established practice for performing hysterectomy and bilateral salpingo-oophorectomy with/without pelvic lymph node dissection according to local cancer network guidelines for FIGO Stage of disease (International Federation of Gynaecology and Obstetrics) as determined by availability of either surgically skilled robotic or laparoscopic surgeons.

Primary Outcome

The primary outcome for this study was the European Organisation for Research and Treatment of Cancer Quality of Life (EORTC QLQ C-30) health related quality of life measure. The EORTC QLQ-C30 is a 30-item questionnaire assessing quality of life in cancer patients. It contains five functional scales (physical, role, cognitive, emotional, and social), three symptom scales (fatigue, pain, and nausea and vomiting), and a global health and quality-of-life scale. The remaining single items assess additional symptoms commonly reported by cancer patients (dyspnoea, appetite loss, sleep disturbance, constipation, and diarrhoea), as well as the perceived financial impact of the disease and treatment. Higher scores indicate better function and heightened symptoms. The QLQ C30 summary score is presented as recommended by the EORTC Quality of Life Group[20]. The summary score is based on 27 items, the quality-of-life scale and the financial impact item are excluded[20].

Secondary Outcomes

For peer review only - http://bmjopen.bmj.com/site/about/guidelines.xhtml

Secondary outcome measures included functional capacity at follow-up measured using the six-minute walk test (6MWT), HRQoL (EQ-5D-5L QALYs), NHS costs and cost-effectiveness. The 6MWT is a sub-maximal test of aerobic capacity which requires the individual to walk as far as possible during six minutes around a 30-meter course and the distance covered is recorded. It has been applied in a clinical setting in a number of conditions as well as pre- and post-operatively[21 22]. The EQ-5D-5L comprises five domains each assessing a specific dimension of HRQOL (mobility, self-care, usual activities, pain, and anxiety and depression) with three response levels (''no problems'', ''some or moderate problems'', and ''extreme problems'')[23 24]. Responses are converted into a weighted single utility score on a scale from 1 (perfect health) to 0 (death) in order to estimate Quality Adjusted Life Years (QALYs)[23 24].

Statistical methods

Reporting of study findings was informed by the Strengthening the Reporting of Observational studies (STROBE) guidelines[25]. Patient characteristics and differences in surgical methods were compared using the appropriate statistical tests (t-test for continuous variables and χ^2 test for categorical variables). The EORTC QLQ C-30 summary score, 6MWT, and EQ-5D-5L utility were evaluated using a constrained baseline longitudinal regression model[26]. The equality at baseline constraint was imposed to counteract regression to the mean, whereby, a group of patients with a worse average baseline score generally improve more than the group with better baseline scores, independent of any intervention effect[27]. This type of regression model also accounts for the hierarchical data structure and provides a principled method for dealing with missing outcome data, including missing baseline data with baseline as part of the outcome vector in this linear mixed model[26]. A Satterwhite correction of degrees of freedom was applied. The model adjusts for potential confounders identified as differences in baseline characteristics and surgical methods as fixed effects, alongside each individual patient modelled as a random effect. Parameters for interaction terms between time and treatment represent the estimated treatment effects at each

For peer review only - http://bmjopen.bmj.com/site/about/guidelines.xhtml

follow-up. Treatment effects are reported with 95% confidence intervals (95% C.I.) and p-values. P-values of less than 0.05 were considered statistically significant. All analyses were undertaken using Stata version 15.1.

Economic Evaluation

Estimating the cost of surgery

The costing perspective was that of the U.K. National Health Service (NHS) with all prices being in pounds sterling (£) using 2018-19 prices. In order to increase precision and transferability into international contexts, the cost of each surgical procedure was micro-costed with resource use including equipment, staffing, and consumables being collected from the study specific case report forms (CRF) and expert clinical guidance. General ward and Intensive Care Unit (ICU) length of stay (LOS) following surgery until discharge were also collected from the study CRF. Following discharge, resource use was collected weekly for 12 weeks post-surgery using a Health Service Utilisation Questionnaire CRF. Follow-up resource use included primary care services e.g., general practice visits, district nurse visits as well as secondary care services e.g., inpatient stays, and outpatient visits. Serious adverse events from initial admission through to final follow-up were also recorded using a study specific CRF. Resource use is reported in supplemental material S1.

Unit costs were derived from routine data sources[28 29] alongside study specific estimates. All re-usable equipment were assigned a 'per use' cost. Where equipment had a short re-usable lifespan (e.g., 10 uses), the unit cost was divided by the number of uses to get a cost per use. Capital equipment such as laparoscopic stack systems and robotic components (as opposed to single or limited use surgical instrumentation) with prolonged lifespans were annuitized using a discount rate of 3.5%[30] to estimate yearly costs. These were then converted to a cost per use by assuming 3 surgical procedures per day across 254 days per year (this is total number of working days Monday to Friday excluding public holidays in England). Serious adverse events that occurred before

For peer review only - http://bmjopen.bmj.com/site/about/guidelines.xhtml

discharge were included in the bed day costs. Serious adverse events that required a hospital admission during follow-up were costed using the appropriate NHS tariff cost[29]. Unit prices are reported in supplemental material S2.

Quality of life

Quality of life was measured using EQ-5D-5L QALYs. To calculate a QALY, results from the EQ-5D-5L were converted into health state utilities at baseline, 2, 4, 12 -weeks using a representative sample of the UK population[31]. These utility values were then used to estimate QALYS at 12 weeks using the area under the curve approach[32].

Estimating Cost-effectiveness

A cost-effectiveness analysis at 12 weeks post-primary procedure was conducted to compare the incremental cost per quality adjusted life-year (QALY). The incremental cost-effectiveness ratio (ICER) was calculated as the difference in costs divided by the difference in QALYs between the two surgical groups using a bootstrap analysis with 1000 simulations. From this, a cost-effectiveness plane was produced, each quadrant indicating whether the surgical procedures were more or less expensive and more or less effective while also illustrating decision uncertainty[33]. The CEAC shows the probability that the surgical procedures are cost-effective, for a range of threshold values for willingness to pay (λ) per additional QALY. In the UK, threshold values of £20 000 to £30 000 per QALY are commonly used[34]. These analyses were conducted using Stata 15.1. The cost effectiveness analysis was reported in accordance to the Consolidated Health Economic Evaluation reporting Standards (CHEERS) statement[35].

One way sensitivity analysis was also conducted for the adjusted cost-effectiveness analysis to investigate how sensitive the results were to the assumptions for the annuitization and per-use cost of the capital equipment. This was conducted assuming different rates of daily usage (once, twice, and four times) across 254 days per year.

For peer review only - http://bmjopen.bmj.com/site/about/guidelines.xhtml

RESULTS

Participants

A total of 275 patients recruited to this study, 29 patients were excluded as they did not have procedures recorded. Of the remaining 246 patients, 168 underwent RALS, 78 underwent CLS. 9 RALS patients and 5 CLS patients were excluded due to missing all outcome data. Overall, 159 RALS and 73 CLS were eligible for analysis. A flow diagram is presented in supplemental material S3.

Baseline characteristics and surgical information of study participants are recorded in Table 1, with further detail available in supplementary S4. The average age, BMI, smoking status, and number of prior surgeries were similar across both the RALS and CLS cohorts. However, there was a higher proportion of Caucasian patients in the CLS compared to the RALS cohort (n=68 (95.77%) versus n=129 (83.77%), p=0.011). The RALS cohort consisted of more patients with higher cancer grades (p=0.004) and higher cancer stages (p=0.012). There was no difference in EORTC or EQ-5D-5L health related quality of life scores at baseline between both cohorts. The CLS cohort travelled more distance in the 6MWT at baseline compared to the RALS patients (386.54 ± 109.71 versus 374.80 ± 365.30, p=0.454).

Table 1: Patient characteristics and surgical information

Descriptor	RALS (n=159)	CLS (n=73)	P
Demographics (baseline)			
**Age (years)**	61.5 ± 11.8	60.8 ± 12.2	0.690
**BMI** Mean ± SD	31.5 ± 8.0	30.6 ± 7.6	0.763
**Smoking Status**			0.849
Non-Smoker	116 (72.96%)	55 (75.34%)	
Ex-Smoker	6 (3.77%)	2 (2.74%)	
Smoker	26 (16.35%)	14 (19.18%)	
**Ethnicity**			0.011
White	129 (83.77%)	68 (93.15%)	
Non-White	25 (16.23%)	3 (4.11%)	
Unknown	11 (6.92%)	2 (2.74%)	
Medical history (baseline)			
**Number of prior surgeries**			0.476

For peer review only - http://bmjopen.bmj.com/site/about/guidelines.xhtml

0	129 (81.12%)	55 (75.34%)	
1	21 (15.72%)	15 (20.55%)	
2	8 (6.92%)	3 (4.11%)	
3	1 (0.63%)	0 (0%)	
Unknown	0 (0%)	0 (0%)	
**Histology**			0.145
Adenocarcinoma	15 (9.43%)	13 (17.81%)	
Aden squamous	2 (1.26%)	0 (0%)	
Benign	12 (7.55%)	10 (13.70%)	
Carcinoma sarcoma	5 (3.14%)	1 (1.37%)	
Clear cell	6 (3.77%)	1 (1.37%)	
Endometrioid	81 (50.94%)	36 (49.32%)	
Sarcoma	1 (0.63%)	1 (1.37%)	
Serous	24 (15.09%)	3 (4.11%)	
Squamous	6 (3.77%)	1 (1.37%)	
Unknown	7 (4.40%)	7 (9.59%)	
**Cancer Grade**			0.004
Benign	12 (7.55%)	10 (13.70%)	
1	41 (25.79%)	29 (39.73%)	
2	27 (16.98%)	11 (15.07%)	
3	46 (28.93%)	7 (9.59%)	
Unknown	33 (20.75%)	16 (21.92%)	
**Cancer Stage**			0.012
No Stage	12 (7.55)	10 (13.70%)	
1	110 (69.18%)	44 (60.27%)	
2	6 (3.77%)	8 (10.96%)	
3	15 (9.43%)	1 (1.37%)	
4	3 (1.89%)	0 (0%)	
Unknown	13 (8.18%)	10 (13.70%)	
Outcomes (baseline)			
**EORTC summary score**	86.29 ± 11. 38	86.79 ± 10.59	0.754
**EQ-5D-3L utility**	0.830 ± 0.150	0.800 ± 0.162	0.195
**6MWD (m)**	374.80 ± 365.30	386.54 ± 109.71	0.454
Surgical methods and length of stay			
**Lymph node dissection**			<0.001
Yes<br>*Full*<br>*Sentinel*	102 (64.14%)<br>*49*<br>*53*	6 (8.22%)<br>*3*<br>*3*	
No	57 (35.85%)	67 (91.78%)	
**Type of procedure**			0.166
Radical	16 (10.06%)	12 (16.44%)	
Simple	143 (89.94%)	61 (83.56%)	
**Type of hysterectomy**			0.263
With parametrium	18 (11.32%)	12 (16.44%)	
Without parametrium	141 (88.68%)	60 (82.19%)	
**Theatre times**	183 (68)	168 (93)	0.179
**Ward Days**	1.3 (2.1)	1.8 (2.4)	0.177
**ICU days**	0.08 (0.31)	0.07 (0.34)	0.774
**Total Length of Stay**	1.4 (2.1)	1.9 (2.4)	0.199
Data presented as n(%) or mean ± SD			

For peer review only - http://bmjopen.bmj.com/site/about/guidelines.xhtml

CLS conventional laparoscopic surgery
RALS robotically assisted laparoscopic surgery

Intra operative and length of stay

Very few patients had a lymph node dissection during CLS surgery compared to RALS (n=6 (8.22%) versus n=102 (64.14%), p=0.001). Of those who had a lymph node dissection in both surgeries, around half had a full lymph node dissection with the remainder having a sentinel procedure alone. The majority of both CLS and RALS procedures were simple hysterectomies (n= 61 (83.56%) and n= 143 (89.94%), p=0.166). Radical hysterectomy made up a very small proportion of patients for both procedures. Hysterectomy without parametrium were n= 60 (83.33%) and n= 141 (88.68%) (p=0.263) in CLS and RALS respectively. Theatre time was shorter on average for CLS (168 ± 93 minutes) compared to the RALS (183 ± 68 minutes) although this was not significant (p=0.179). Length of stay (LoS) in terms of general ward bed days was higher for CLS relative to RALS (1.8 ± 2.4 days versus 1.3 ± 2.1 days, p=0.177). Very few patients required an ICU stay for either surgical procedure (mean LOS of 0.07 ± 0.34 for CLS, 0.08 ± 0.31 for RALS, p=0.774). As such, total LoS was slightly higher on average for CLS compared to the RALS (1.9 ± 2.4 days versus 1.4 ± 2.1 days, p=0.199). There were no deaths in either cohort up until first discharge date.

Serious adverse events

There were 13 serious adverse events (SAE) recorded during this study (supplemental material S5), 6 occurred in CLS and 7 in RALS. For CLS, three occurred following the procedure but before discharge with another three were detected over the first two weeks of follow-up. For RALS, there were 6 SAEs prior to discharge, and 1 SAE in week 2 of follow-up.

Primary Outcome

The crude and adjusted treatment effects for all outcomes are presented in Table 2. Mean EORTC HRQOL at the 2-week follow-up compared to baseline fell for both RALS and CLS (-15.22 ± 13.82, -15.97 ± 15.28). The adjusted between-group difference (treatment effect) in EORTC from baseline to

For peer review only - http://bmjopen.bmj.com/site/about/guidelines.xhtml

week 2 post-surgery was higher for RALS (1.6 (95% CI -2.8 to 6.1)). The mean EORTC at week 4 for both surgeries were also lower than baseline (-5.95 ± 13.31 for RALS and -4.49 ± 13.65 for CLS). The adjusted between group difference in the EORTC score from baseline to week 4 was lower for RALS (-0.3 (95% CI -4.8 to 4.1)). By 12 weeks follow-up, the EORTC score compared to baseline, had improved in both RALS (0.85 ± 11.36) and CLS (2.09 ± 11.45) respectively with an adjusted between group difference of 0.4 (95% CI -4.1 to 4.9) in favour of RALS.

Table 2: Crude change in outcomes from baseline at different follow-up points and estimated adjusted treatment effects with 95% C.I.

**Time-point**	**Number of participants**		**Crude change from baseline ***		**Estimated treatment effect (group difference) in mean change† with 95% C.I.**	**P-value**
	**RALS**	**CLS**	**RALS**	**CLS**		
Primary Outcome						
**EORTC score**						
2-week follow-up	114	55	-15.22 ± 13.82	-15.97 ± 15.28	1.6 (-2.8 to 6.1)	0.469
4-week follow-up	112	55	-5.95 ± 13.31	-4.49 ± 13.65	-0.3 (-4.8 to 4.1)	0.884
12-week follow-up	103	51	0.85 ± 11.36	2.09 ± 11.45	0.4 (-4.1 to 4.9)	0.861
Secondary Outcomes						
**6MWD (m)**						
2-week follow-up	86	52	-12.31 ± 84.05	-13.71 ± 104.04	18.1 (-11.0 to 47.2)	0.223
4-week follow-up	102	49	15.46 ± 71.03	31.08 ± 86.96	-3.4 (-33.2 to 26.4)	0.823
12-week follow-up	82	35	27.33 ± 86.94	32.48 ± 74.11	3.4 (-27.6 to 34.4)	0.830
**EQ-5D-3L utility**						
2-week follow-up	115	57	-0.11 ± 0.20	-0.09 ± 0.21	0.036 (-0.023 to 0.095)	0.227
4-week follow-up	114	55	-0.06 ± 0.16	-0.01 ± 0.20	0.010 (-0.050 to 0.069)	0.745
12-week follow-up	107	50	0.01 ± 0.16	0.02 ± 0.20	-0.002 (-0.061 to 0.058)	0.959

*Crude change from baseline given as mean change ± SD of this change
† Estimated treatment effect for all outcomes are the between group differences in the change from baseline derived from the mixed effects model adjusting for lymph node dissection and cancer stage as fixed effects
C.I. Confidence Interval, SD Standard Deviation, 6MWD six-minute walk distance, EORTC European Organisation for Research and Treatment of Cancer Quality of Life, CLS conventional laparoscopic surgery, RALS robotically assisted laparoscopic surgery

For peer review only - http://bmjopen.bmj.com/site/about/guidelines.xhtml

Secondary Outcomes

For both RALS and CLS, EQ-5D utility scores were lower in weeks 2 and 4 compared to baseline but were higher by week 12. The distance travelled in the 6MWT was lower for RALS and CLS in week 2 compared to baseline but was higher in weeks 4 and 12. However the adjusted analysis for both outcomes showed no statistically significant treatment effect.

Costs

Complete data was obtained for each participant regarding procedure costs and costs up until discharge. Summary costs of procedure and follow-up for both RALS and CLS are presented in supplemental material S6. Procedure costs included costs relating to staff, theatre time, equipment, and length of stay from admission to discharge. The mean procedure cost was higher for RALS compared to CLS (£4 758 ± 972 versus £3 741 ± 1 350, p<0.001). This was due to RALS having a higher mean staff costs (£1 851 ± 688 versus, £1 703 ± 940, p=0.231), theatre costs (£765 ± 284 versus £704 ± 388, p=0.231), and equipment costs (£2 134 versus £1 325). However, the mean cost for total LoS following the procedure was lower for RALS (£704 ± 1 002 versus £851 ± 1 159, p=0.352). Overall, the total mean cost from admission to discharge including the procedure costs were higher for RALS compared to CLS (£5 462 ± 1 472 versus £4 592 ± 2 072, p= 0.002).

Follow-up resource was collected up to 12 weeks post-discharge. In weeks 1-3 follow-up, post-discharge mean costs were higher for CLS relative to RALS, likely due to higher overnight stays in hospital. Thereafter, mean costs incurred in each week were similar between the two groups. Costs in week 12 were higher for RALS due to secondary care costs.

Cost-effectiveness analysis

For peer review only - http://bmjopen.bmj.com/site/about/guidelines.xhtml

Table 3 reports the outcomes of the constrained baseline longitudinal regression model unadjusted (no confounder) and adjusted cost-effectiveness analyses with Figure 1 presenting the cost-effectiveness acceptability curves and the willingness to pay.

Table 3: Results for the cost-effectiveness models

	Unadjusted Analysis	Adjusted Analysis ‡
Cost Difference * (£)	556 (-314 to 1 315)	-188 (-1 321 to 827)
QALYS Difference *	0.0024 (-0.0005 to 0.0057)	0.0024 (-0.0010 to 0.0057)
Non-parametric ICER (£ per QALY)	231 667	Dominated
Willingness to Pay (£20,000 threshold) †	0.117	0.657
Willingness to Pay (£25,000 threshold) †	0.126	0.664
Willingness to Pay (£30,000 threshold) †	0.133	0.671

Differences are shown as RALS – CLS.
* Reported as mean (95% confidence interval)
† Proportion of bootstrapped iterations in which RALS is cost-effective
‡ Models adjust for lymph node dissections and cancer stage
CLS conventional laparoscopic surgery, RALS robotically assisted laparoscopic surgery

For the unadjusted cost-effectiveness analysis, including follow-up over 12 weeks, the RALS cohort had a mean total cost difference of £556 (95%CI -£314 to £1 315) compared to CLS and a mean QALY difference of 0.0024 (95%CI -0.00051 to 0.0057). This resulted in a non-parametric ICER of £152 843 per QALY. At a willingness to pay threshold of £20 000 per QALY, 11.7% of the bootstrapped iterations resulted in RALS surgery being cost-effective, rising to 13.3% of iterations being cost-effective at £30 000 per QALY.

When adjusting for lymph node dissections and cancer stage, over 12 weeks, RALS had a mean cost difference of £-188 (95%CI -£1 321 to £827) compared to CLS and a mean QALY difference of 0.0024 (95%CI -0.0008 to 0.0057). This resulted in a non-parametric ICER being dominant. At a willingness to

For peer review only - http://bmjopen.bmj.com/site/about/guidelines.xhtml

pay threshold of £20 000 per QALY, 65.7% of the bootstrapped iterations resulted in RALS being cost-effective, rising slightly higher to 67.1% of iterations being cost-effective at a £30 000 per QALY threshold.

Sensitivity analysis

The results of the one-way sensitivity analyses are presented in Table 4 with the cost-effectiveness plane, and willingness to pay figures presented supplemental material S7. RALS surgery had a higher average cost compared to CLS when assuming only 1 procedure per day (mean cost difference £430 (95%CI £-703 to £1 445)), with a resulting non-parametric ICER of £179 167 per QALY. When assuming only 1 procedure per day, only 23.8% of the bootstrapped iterations are cost-effective with a willingness to pay of £20 000 per QALY. When assuming 2 or 4 procedures per day, the cost of the robot is lower than CLS (mean cost difference: £-34 (95% CI £-1 167 to £981) and £-266 (£-1 399 to £749)) respectively. The non-parametric ICER for both scenarios were dominant in both cases, suggesting that RALS surgery provides more QALYs and has a lower cost compared to CLS. Assuming two procedures per day, RALS was cost-effective for 53.6% of bootstrapped iterations, and when assuming four procedures per day RALS was cost effective for 71.4% of the bootstrapped iterations at a willingness to pay of £20 000 per QALY.

Table 4: Results of the one-way sensitivity analysis of the adjusted cost-effectiveness model, varying the number of procedures performed per day.

	One procedure per day ‡	Two procedures per day ‡	Four procedures per day ‡
Cost Difference * (£)	430 (-703 to 1 445)	-34 (-1 167 to 981)	-266 (-1 399 to 749)
QALYS Difference *	0.0024 (-0.0010 to 0.0057)	0.0024 (-0.0010 to 0.0057)	0.0024 (-0.0010 to 0.0057)
Non-parametric ICER (£ per QALY)	179 167	Dominated	Dominated
Willingness to Pay (£20,000 threshold) †	0.238	0.536	0.700

For peer review only - http://bmjopen.bmj.com/site/about/guidelines.xhtml

Willingness to Pay (£25,000 threshold) †	0.246	0.553	0.703
Willingness to Pay (£30,000 threshold) †	0.256	0.569	0.714
Differences are shown as RALS – CLS.<br>* Reported as mean (95% confidence interval)<br>† Proportion of bootstrapped iterations in which RALS is cost-effective<br>‡ Models adjust for lymph node dissections and cancer stage<br>CLS conventional laparoscopic surgery, RALS robotically assisted laparoscopic surgery			

For peer review only

DISCUSSION

The delivery of surgical care in gynaecological cancer in the UK National Health Service (NHS) continues to evolve. Minimal access surgery remains the mainstay for treating patients with endometrial cancer. Robotic surgery continues to be rolled out across NHS organisations but there is no national program to coordinate this, and the availability of robotic surgery is dependent on local trust finances and agreements with local commissioning groups[36]. Thus, the type of surgical procedure a patient is offered to treat gynaecological malignancy is dependent upon their postcode and what is available in their local NHS hospital.

This pragmatic prospective comparative observational cohort study compares outcomes and cost effectiveness for patients undergoing conventional laparoscopic surgery or robotically assisted laparoscopic surgery reflecting real world care pathways. The results in terms of impact on functional outcomes and HRQoL showed very little difference between the two surgical methods and are in keeping with previous findings[37 38]. Therefore, the results of the cost-effectiveness analyses were largely driven by costs, including the cost of procurement and maintenance of the Robotic device and subsequent changes in resource use after surgery.

The unadjusted cost-effectiveness analysis in our study suggested that RALS was not cost-effective using the National Institute for Health and Care Excellence (NICE) threshold, of £30 000/€ 34 000[39].

For peer review only - http://bmjopen.bmj.com/site/about/guidelines.xhtml

However, as with any observational study there were differences in characteristics of the patients in each of the surgical groups which led to confounding and bias in the unadjusted analysis. The characteristics in question were related to higher cancer stages and a higher number of lymph node evaluations in the RALS group. Once these adjustments were made to the analysis, the probability of RALS being cost-effective was much higher. The difference in cost-effectiveness between the unadjusted and adjusted base case analysis was driven by cost differences. One possible explanation for this difference may be that the higher cancer stages and increased number of lymph node evaluations in the RALS cohort resulted in longer theatre times and subsequently higher theatre and staff costs.

Despite national guidelines[40] on best practice for lymph node evaluation, current practice varies across the UK dependent upon local resources and surgical paradigm. Further, we noted that some hysterectomies had been classified as 'radical', yet they had not had parametrial or lymph node dissections. Further analysis (data not shown) revealed that these patients had surgery for endometrial cancer rather than cervical cancer. The explanation for this lay in the way in that NHS Trusts coded their surgical procedures on the basis of commissioning because this had implications for payments to Trusts on procedures performed. For example, some operations were coded as a modified radical hysterectomy if there was any element of ureterolysis. This meant that some endometrial cancers were described as being 'modified radical hysterectomy' but lymph node dissections may not have been indicated, as per local guidelines. The authors confirmed in those centres where this discrepancy was identified that all gynae oncologists would perform full bilateral pelvic lymphadenectomies for those patients having a radical hysterectomy for cervical cancer.

To our knowledge this study is the first to conduct a multi-center full cost-effectiveness evaluation comparing RALS versus CLS for hysterectomies following gynaecological cancer. Other studies in this area only focus on costs. An older prospective single center study comparing costs of RALS versus CLS was conducted by Sarlos, et al. [41]. This study collected prospective data from June 2007 to May

For peer review only - http://bmjopen.bmj.com/site/about/guidelines.xhtml

2009 of 40 RALS patients following the introduction of robotic surgery in a single centre and compared this with a matched set of 40 CLS patients from retrospective records. Patients were matched 1:1 according to surgeon, uterus weight, BMI, and age. Unlike the findings of our adjusted analysis, Sarlos, et al. [41] estimated a higher cost of RALS £4066.84 compared to CLS £2150.76. However, we note that although the costs of Sarlos, et al. [41] are micro costed, they do not include the cost of the Robotic device. Furthermore, one potential driver of higher costs of RALS in their study could be due to surgeon inexperience. The RALS centres in our study include surgeons who were experienced in performing RALS and likely represents a truer reflection of practice today. As they conducted a matched case control study, Sarlos, et al. [41] do not present adjusted costs. However, we argue that there are potential limitations in the matching used as no consideration is given to patient comorbidities, which have been identified as drivers of cost [42].

Another study by Ind et al[43] investigated the costs of RALS compared CLS in a prospective cohort study of a single centre in England, where surgeons and surgical teams had prior experience with robotic surgery. It was noted that the robotic device was donated by a charity. Although the robotic device was donated to the centre, the micro costing conducted by Ind, et al. [43] also included a depreciated per use cost of the purchase of the Robotic device. The results of Ind et al are similar to our own study, with the 30-day cost of RALS estimated to be lower at £8 481 compared to CLS at £9 979. However, it should be noted that the cost estimates of both interventions are much higher than in our own study due to Ind, et al. [43] reporting much higher theatre costs, length of stay, and high dependency use. For example, Ind, et al. [43]. report a median length of stay of 3 days for CLS and 2 days for RALS with 80.5% of CLS and 70.8% of RALS patients requiring high-dependency post-operative care. This differs from our own findings in which median length of stay for CLS and Robotic surgery was 1 day each, with very few patients in either arm requiring stay in ICU. Furthermore, length of time for surgery reported by Ind, et al. [43] was higher for both interventions compared to our study. These differences may be due to factors such as changes in practice, advances in both RALS and CLS, or due to small sample size of Ind, et al. [43].

For peer review only - http://bmjopen.bmj.com/site/about/guidelines.xhtml

A larger, more recent retrospective cohort study was conducted by Moss et al [42] comparing costs of different routes of surgery for the management of endometrial cancer with up to 1 year follow up using NHS England HES data between 2011-2018. The unadjusted cost at 90 days in the RALS cohort was on average £246 more expensive compared to CLS. However, the patients undergoing RALS had higher Charlson Comorbidity Index scores (CCI) and an increased number were obese compared to CLS. When controlling for the differences between the cohorts, Moss, et al. [42] estimated a reduced cost difference at 90 days with RALS being only £89 more expensive. These results are similar to our own findings with the cost difference identified by Moss, et al. [42] situated within the 95% confidence interval of the cost difference in our adjusted analysis £-188 95%CI (-1 321 to 827). However, there are still some limitations to their study compared to our own. Firstly, Moss, et al. [42] utilised HRG tariff to estimate costs rather than detailed micro costing. Furthermore, unlike our detailed micro costing the HRG tariff estimates do not include capital costs or maintenance costs of the robotic devices, which as our study shows is a large and important component of surgical costs with robotic surgery. As a prospective multicentre cohort study, our study uses a much more rigorous inclusion criteria compared to Moss, et al. [42] and data collection of resource use may be more accurate in our study as we do not rely on HES data and also capture primary care resource use.

The sensitivity analysis conducted as part of our study identified that results of the cost-effectiveness analysis were highly sensitive to the volume of procedures performed. The finding that increasing the utilisation of the robotic devices increases the probability of RALS being cost effective is in keeping with cost-effectiveness analyses comparing robotic surgery in other pathologies[44]. We therefore argue that the relationship between usage and cost-effectiveness of RALS should stimulate centres using robotic devices to maximise the volume of cases that are carried out using this equipment.

Acquisition of surgical robots has been managed in England to date by individual hospital trusts and a reliance on charitable donations with no centralised strategy. This may have unintended

For peer review only - http://bmjopen.bmj.com/site/about/guidelines.xhtml

consequences for health service provision. Firstly, trusts may feel obligated to prioritise treatment of diseases on which the donating charity is focussed, even where this may not be cost-effective. Furthermore, we question the long-term viability of relying on charitable donations, particularly when the robotic devices become obsolete and reach the end of their life span and need to be replaced. Overreliance on charitable donations may not be sustainable into the future as RALS moves to becoming more mainstream.

Lam et al. examined the adoption of surgical robots through a nationwide Freedom of Information Request to all acute NHS trusts in England[36] and confirmed our argument regarding increasing the utilisation of the robotic equipment. Specifically, their results suggested that despite the number of robotic procedures increasing, national accessibility to robotic services and case volumes were highly variable with many trusts having low volume use representing missed efficiency savings for the NHS. We therefore argue that policies regarding the deployment and use of robotic surgical equipment are needed at national, regional and/or hospital level depending on the configuration of the health care system. One possible solution to this challenge in publicly funded national health systems such as the U.K NHS is the centralisation of robotic centres into 'innovation hubs' than can establish best practice, clinical evidence, and patient volumes[36]. In the absence of centralised initiatives, hospital operational policies should aim to procure robotic equipment over a range of surgical specialities parallel to a drive to undertake procedures in extended time slots to aid cost-effectiveness.

Limitations

We acknowledge the limitations of this study as a pragmatic prospective cohort study. This was necessary because of the current geography and delivery of RALS and CLS and the hub and spoke structure of cancer services in the UK. The fact that the two populations of patients differed in simple factors such as grade and stage of cancer has impacted the generalisability of this data. Of some reassurance in terms of justifying robotic costs in endometrial cancer is the finding that the higher stage and grade patient cohort showed a swing in cost effectiveness favouring robotics

For peer review only - http://bmjopen.bmj.com/site/about/guidelines.xhtml

despite worse prognostic factors. A randomised multi centre prospective trial would circumvent this limitation and provide data with greater generalisability to organisations wanting to establish the cost effectiveness of robotics ahead of implementation of a programme in their institution. However, design of such a trial would need significant and careful consideration to overcome the difficulties of acceptability to patients and surgeons alike so that recruitment and ownership of the trial made it successful. In addition, the expansion of RALS across the NHS is advancing and could make it difficult to recruit enough CLS participants in any future trial. Identifying cost-effectiveness through the gold standard RCT approach may not be feasible and as such this study is of great value in adding to the literature regarding the cost-effectiveness comparison of RALS and CLS and helping to inform decision makers on efficient use of resources.

A further limitation of this study is that confounding adjustment was managed within a regression model by including control variables. This required a well specified model to reduce residual confounding and consequent bias. Propensity scoring is an alternative and increasingly popular method of controlling potential confounding in observational studies that compare the effectiveness of healthcare interventions[45 46]. Propensity matching is typically used in cohort studies and involves fitting regression models to predict treatment groups based on selected characteristics[46]. However, there is evidence to suggest that there is equivalence between confounding adjustment to various propensity score-based approaches[47 48].

Conclusion

This study identified no differences in HRQoL or functional outcomes between patients undergoing RALS or CLS for treatment of confirmed and suspected gynaecological endometrial and cervical cancers. However, this study has shown that there is evidence to suggest that RALS may be cost-effective compared to CLS, but results are highly sensitive to assumptions around the usage, and subsequent per use cost, of the robotic hardware. As robotic surgery becomes more mainstream, it

For peer review only - http://bmjopen.bmj.com/site/about/guidelines.xhtml

is of vital importance that utilisation of robots are optimised to ensure efficient resource use. Further investigation is needed in this area is needed and a non-inferiority RCT would be of benefit in decision makers with regards to cost-effectiveness, however this may not be practical or achievable in the NHS.

Author contributions

Conceptualisation: JG, PM, CN, MN. JL. JT

Data curation: AM DS SM

Formal analysis: AM, DS, JG, PM, SM

Funding acquisition: JG JT

Investigation: all authors

Methodology: AM DS JG SM PM

Writing-original draft: AM JG

Writing- review & editing: all authors

Final approval: all authors

Competing Interests

All authors have completed the ICMHE uniform disclosure form at http://www.icmje.org/disclosure-of-interest/ and declare: all authors had financial support from Intuitive for the submitted work. JL has received renumeration for time given as a proctor at intuitive, MN has received payment from intuitive for consultation and also for time given as a proctor and for development of educational lectures, in addition MN is President of the British & Irish Association of Robotic Gynaecological Surgeons (BIARGS); no other relationships or activities that could appear to have influenced the submitted work.

For peer review only - http://bmjopen.bmj.com/site/about/guidelines.xhtml

Ethics

This NIHR Portfolio research study fully conformed to the ethical norms and standards in the Declaration of Helsinki. The study received the appropriate regulatory approvals in the UK before proceeding having been approved by an NHS Research Ethics Committee and registered with each participating NHS Trust as per their local Research department's process. South Tees Hospitals NHS Foundation Trust as sponsor and trial coordinating organisation carried out several duties (such as provision of Site Initiation Visits and check-in calls, data validation reports, monitoring visits) to promote high quality trial conduct and adherence to Good Clinical Practice.

Acknowledgements

Sarah Kiddell (formerly Clinical Trial Practitioner for South Tees Hospitals NHS Foundation Trust) was the Trial Manager with primary responsibility for the day-to-day running of the study and reporting duties.

Richard Hodgson (formerly Research Data Officer for South Tees Hospitals NHS Foundation Trust), followed by Lesley Harris (formerly Research Data Officer for South Tees Hospitals NHS Foundation Trust) were the Data Support Officers checking data completeness and accuracy.

Julie Rowbotham (formerly Research Manager for South Tees Hospitals NHS Foundation Trust) acted as Sponsor Representative for the research study, followed by Joe Millar (Research Governance Manager for South Tees Hospitals NHS Foundation Trust). Both were responsible for overseeing the delivery of sponsorship responsibilities and providing research governance advice throughout.

The following contacts are all acknowledged for their role as Principal Investigator for each of the listed research sites:

Mr. Simon Butler-Manuel, Royal Surrey NHS Foundation Trust

Mr. Allan Gillespie, The Sheffield Teaching Hospitals NHS Foundation Trust

For peer review only - http://bmjopen.bmj.com/site/about/guidelines.xhtml

Mr. Pierre Martin-Hirsch, Lancashire Teaching Hospitals NHS Foundation Trust

Mr. Richard Edmondson, Manchester University NHS Foundation Trust

Mr. Mike Smith, The Christie NHS Foundation Trust

Mr. David Milliken, Somerset NHS Foundation Trust

Mr. Partha Sengupta, Country Durham & Darlington NHS Foundation Trust

Mr. Tony Challoub, Newcastle upon Tyne Hospitals NHS Foundation Trust

Data accessibility statement

The complete research data supporting the findings of this paper were all stored in a web research database maintained by the funder Intuitive, which has now been data locked and exported as an excel record held by both Intuitive and the project team. Data is available on request due to privacy/ethical restrictions.

The corresponding author affirms that the manuscript is an honest, accurate, and transparent account of the study being reported. No important aspects of the study have been omitted and any discrepancies from the study as planned have been disclosed.

Funder statement

The study was supported by Intuitive through a £93,850 funding grant and provision of a secure data management web service. However, the study was managed and conducted by a wholly independent project team. The grant funding paid for identified project costs such as project management staff support, statistician and health economist support, equipment, supplies and hospital staff support.

Public and patient involvement

Patient and Public Involvement were not included in this research project.

For peer review only - http://bmjopen.bmj.com/site/about/guidelines.xhtml

References

1. Press JZ, Gotlieb WH. Controversies in the treatment of early stage endometrial carcinoma. *Obstet Gynecol Int* 2012;2012:578490. doi: 10.1155/2012/578490 [published Online First: 2012/06/12]

2. Janda M, Gebski V, Brand A, et al. Quality of life after total laparoscopic hysterectomy versus total abdominal hysterectomy for stage I endometrial cancer (LACE): a randomised trial. *Lancet Oncol* 2010;11(8):772-80. doi: 10.1016/s1470-2045(10)70145-5 [published Online First: 2010/07/20]

3. Kyrgiou M, Swart AM, Qian W, et al. A Comparison of Outcomes Following Laparoscopic and Open Hysterectomy With or Without Lymphadenectomy for Presumed Early-Stage Endometrial Cancer: Results From the Medical Research Council ASTEC Trial. *Int J Gynecol Cancer* 2015;25(8):1424-36. doi: 10.1097/igc.0000000000000521 [published Online First: 2015/08/06]

4. Wright JD, Ananth CV, Lewin SN, et al. Robotically assisted vs laparoscopic hysterectomy among women with benign gynecologic disease. *Jama* 2013;309(7):689-98. doi: 10.1001/jama.2013.186 [published Online First: 2013/02/21]

5. Matt A, Michelle, H. & Barry, O. C. . New developments Robot-assisted surgery in gynaecology *Learning objectives : Ethical issues* 2011:183–88.

6. Boggess JF, Gehrig PA, Cantrell L, et al. A case-control study of robot-assisted type III radical hysterectomy with pelvic lymph node dissection compared with open radical hysterectomy. *Am J Obstet Gynecol* 2008;199(4):357.e1-7. doi: 10.1016/j.ajog.2008.06.058 [published Online First: 2008/10/22]

7. Eklind S, Lindfors A, Sjöli P, et al. A prospective, comparative study on robotic versus open-surgery hysterectomy and pelvic lymphadenectomy for endometrial carcinoma. *Int J Gynecol Cancer* 2015;25(2):250-6. doi: 10.1097/igc.0000000000000357 [published Online First: 2015/01/23]

For peer review only - http://bmjopen.bmj.com/site/about/guidelines.xhtml

8. Patzkowsky KE, As-Sanie S, Smorgick N, et al. Perioperative outcomes of robotic versus laparoscopic hysterectomy for benign disease. *JSLS* 2013;17(1):100-06. doi: 10.4293/108680812X13517013317914

9. Sert BM, Boggess JF, Ahmad S, et al. Robot-assisted versus open radical hysterectomy: A multi-institutional experience for early-stage cervical cancer. *European Journal of Surgical Oncology (EJSO)* 2016;42(4):513-22. doi: https://doi.org/10.1016/j.ejso.2015.12.014

10. Desille-Gbaguidi H, Hebert T, Paternotte-Villemagne J, et al. Overall care cost comparison between robotic and laparoscopic surgery for endometrial and cervical cancer. *European Journal of Obstetrics & Gynecology and Reproductive Biology* 2013;171(2):348-52. doi: https://doi.org/10.1016/j.ejogrb.2013.09.025

11. Liu H, Lawrie TA, Lu D, et al. Robot-assisted surgery in gynaecology. *Cochrane Database of Systematic Reviews* 2014(12) doi: 10.1002/14651858.CD011422

12. England N. Clinical Commissioning Policy : Robotic-Assisted Surgical Procedures for Prostate Cancer, 2015:1-14.

13. England N. 2015/16 Enhanced Tariff Option. 2015

14. Calvert M, Blazeby J, Altman DG, et al. Reporting of patient-reported outcomes in randomized trials: the CONSORT PRO extension. *Jama* 2013;309(8):814-22. doi: 10.1001/jama.2013.879 [published Online First: 2013/02/28]

15. Centre HaSCI. Background information about PROMs, 2015.

16. Centre HaSCI. Outcomes Framework Indicators. 2015

17. Herling SF, Møller AM, Palle C, et al. Health-related quality of life after robotic-assisted laparoscopic hysterectomy for women with endometrial cancer — A prospective cohort study. *Gynecologic Oncology* 2016;140(1):107-13. doi: https://doi.org/10.1016/j.ygyno.2015.10.024

18. Kumar S, Paiz A, Santoso J, et al. A retrospective study to compare quality of life in patients surgically treated by total abdominal hysterectomy versus robotic assisted hysterectomy for

For peer review only - http://bmjopen.bmj.com/site/about/guidelines.xhtml

early stage endometrial cancer. *Gynecologic Oncology* 2012;127(1):S24-S25. doi: https://doi.org/10.1016/j.ygyno.2012.07.068

19. Preston NJ, Wilson N, Wood NJ, et al. Patient-reported outcome measures for use in gynaecological oncology: a systematic review. *Bjog* 2015;122(5):615-22. doi: 10.1111/1471-0528.13251 [published Online First: 2015/01/07]

20. Giesinger JM, Kieffer JM, Fayers PM, et al. Replication and validation of higher order models demonstrated that a summary score for the EORTC QLQ-C30 is robust. *J Clin Epidemiol* 2016;69:79-88. doi: 10.1016/j.jclinepi.2015.08.007 [published Online First: 2015/09/04]

21. Holland AE, Spruit MA, Troosters T, et al. An official European Respiratory Society/American Thoracic Society technical standard: field walking tests in chronic respiratory disease. *European Respiratory Journal* 2014;44(6):1428-46. doi: 10.1183/09031936.00150314

22. Laboratories ACoPSfCPF. ATS statement: guidelines for the six-minute walk test. *Am J Respir Crit Care Med* 2002;166:111-17.

23. Rabin R, Gudex C, Selai C, et al. From translation to version management: a history and review of methods for the cultural adaptation of the EuroQol five-dimensional questionnaire. *Value Health* 2014;17(1):70-6. doi: 10.1016/j.jval.2013.10.006 [published Online First: 2014/01/21]

24. Group TE. EuroQol--a new facility for the measurement of health-related quality of life. *Health Policy* 1990;16(3):199-208. doi: 10.1016/0168-8510(90)90421-9 [published Online First: 1990/11/05]

25. von Elm E, Altman DG, Egger M, et al. The Strengthening the Reporting of Observational Studies in Epidemiology (STROBE) statement: guidelines for reporting observational studies. *The Lancet* 2007;370(9596):1453-57. doi: https://doi.org/10.1016/S0140-6736(07)61602-X

26. Coffman CJ, Edelman D, Woolson RF. To condition or not condition? Analysing 'change' in longitudinal randomised controlled trials. *BMJ Open* 2016;6(12):e013096. doi: 10.1136/bmjopen-2016-013096

For peer review only - http://bmjopen.bmj.com/site/about/guidelines.xhtml

27. Bland JM, Altman DG. Regression towards the mean. *Bmj* 1994;308(6942):1499. doi: 10.1136/bmj.308.6942.1499 [published Online First: 1994/06/04]

28. Curtice L. BA. Unit Costs of Health and Social Care 2019. In: Unit PSSR, ed. Canterbury: University of Kent, 2019.

29. Care DoHaS. NHS Reference Costs 2018 to 2019. In: Care DoHaS, ed. London, 2019.

30. Walker D, Kumaranayake L. Allowing for differential timing in cost analyses: discounting and annualization. *Health Policy Plan* 2002;17(1):112-8. doi: 10.1093/heapol/17.1.112 [published Online First: 2002/02/28]

31. Szende A JB, Cabases J, editors. Self-Reported Population Health: An International Perspective based on EQ-5D. Dordecht (NL)2014.

32. Manca A, Hawkins N, Sculpher MJ. Estimating mean QALYs in trial-based cost-effectiveness analysis: the importance of controlling for baseline utility. *Health Economics* 2005;14(5):487-96. doi: https://doi.org/10.1002/hec.944

33. Fenwick E, Claxton K, Sculpher M. Representing uncertainty: the role of cost-effectiveness acceptability curves. *Health Econ* 2001;10(8):779-87. doi: 10.1002/hec.635 [published Online First: 2001/12/18]

34. Excellence NIfHaC. Guide to the methofs of technology appraisal. London, 2018.

35. Husereau D, Drummond M, Augustovski F, et al. Consolidated Health Economic Evaluation Reporting Standards 2022 (CHEERS 2022) statement: updated reporting guidance for health economic evaluations. *BMC Medicine* 2022;20(1):23. doi: 10.1186/s12916-021-02204-0

36. Lam K, Clarke J, Purkayastha S, et al. Uptake and accessibility of surgical robotics in England. *The International Journal of Medical Robotics and Computer Assisted Surgery* 2021;17(1):e2174. doi: https://doi.org/10.1002/rcs.2174

37. Kim JY, Lee YH, Chong GO, et al. Comparative Study Between Total Laparoscopic and Total Robotic Radical Hysterectomy for Cervical Carcinoma: Clinical Study. *Anticancer Res* 2015;35(9):5015-21. [published Online First: 2015/08/09]

For peer review only - http://bmjopen.bmj.com/site/about/guidelines.xhtml

38. Roh HF, Nam SH, Kim JM. Robot-assisted laparoscopic surgery versus conventional laparoscopic surgery in randomized controlled trials: A systematic review and meta-analysis. *PLoS One* 2018;13(1):e0191628-e28. doi: 10.1371/journal.pone.0191628

39. McCabe C, Claxton K, Culyer AJ. The NICE Cost-Effectiveness Threshold. *PharmacoEconomics* 2008;26(9):733-44. doi: 10.2165/00019053-200826090-00004

40. Morrison J, Balega J, Buckley L, et al. British Gynaecological Cancer Society (BGCS) uterine cancer guidelines: Recommendations for practice. *European Journal of Obstetrics and Gynecology and Reproductive Biology* 2022;270:50-89. doi: 10.1016/j.ejogrb.2021.11.423

41. Sarlos D, Kots L, Stevanovic N, et al. Robotic hysterectomy versus conventional laparoscopic hysterectomy: Outcome and cost analyses of a matched case–control study. *European Journal of Obstetrics and Gynecology and Reproductive Biology* 2010;150(1):92-96. doi: 10.1016/j.ejogrb.2010.02.012

42. Moss EL, Morgan G, Martin A, et al. Economic evaluation of different routes of surgery for the management of endometrial cancer: a retrospective cohort study. *BMJ Open* 2021;11(5):e045888. doi: 10.1136/bmjopen-2020-045888

43. Ind TEJ, Marshall C, Hacking M, et al. Introducing robotic surgery into an endometrial cancer service–a prospective evaluation of clinical and economic outcomes in a UK institution. *The International Journal of Medical Robotics and Computer Assisted Surgery* 2016;12(1):137-44. doi: https://doi.org/10.1002/rcs.1651

44. Labban M, Dasgupta P, Song C, et al. Cost-effectiveness of Robotic-Assisted Radical Prostatectomy for Localized Prostate Cancer in the UK. *JAMA Network Open* 2022;5(4):e225740-e40. doi: 10.1001/jamanetworkopen.2022.5740

45. Kuss O, Blettner M, Börgermann J. Propensity Score: an Alternative Method of Analyzing Treatment Effects. *Dtsch Arztebl Int* 2016;113(35-36):597-603. doi: 10.3238/arztebl.2016.0597 [published Online First: 2016/09/24]

For peer review only - http://bmjopen.bmj.com/site/about/guidelines.xhtml

46. Freemantle N, Marston L, Walters K, et al. Making inferences on treatment effects from real world data: propensity scores, confounding by indication, and other perils for the unwary in observational research. *Bmj* 2013;347:f6409. doi: 10.1136/bmj.f6409 [published Online First: 2013/11/13]

47. Stürmer T, Schneeweiss S, Brookhart MA, et al. Analytic strategies to adjust confounding using exposure propensity scores and disease risk scores: nonsteroidal antiinflammatory drugs and short-term mortality in the elderly. *Am J Epidemiol* 2005;161(9):891-98. doi: 10.1093/aje/kwi106

48. Kurth T, Walker AM, Glynn RJ, et al. Results of multivariable logistic regression, propensity matching, propensity adjustment, and propensity-based weighting under conditions of nonuniform effect. *Am J Epidemiol* 2006;163(3):262-70. doi: 10.1093/aje/kwj047 [published Online First: 2005/12/24]

Figure 1: Graph A: Cost-effectiveness plane for unadjusted analysis. Graph B: Willingness to pay for unadjusted analysis. Graph C: Cost-effectiveness plane for adjusted analysis. Graph D: Willingness to pay for adjusted analysis.

For peer review only

For peer review only - http://bmjopen.bmj.com/site/about/guidelines.xhtml

Figure 1: Graph A: Cost-effectiveness plane for unadjusted analysis. Graph B: Willingness to pay for unadjusted analysis. Graph C: Cost-effectiveness plane for adjusted analysis. Graph D: Willingness to pay for adjusted analysis.

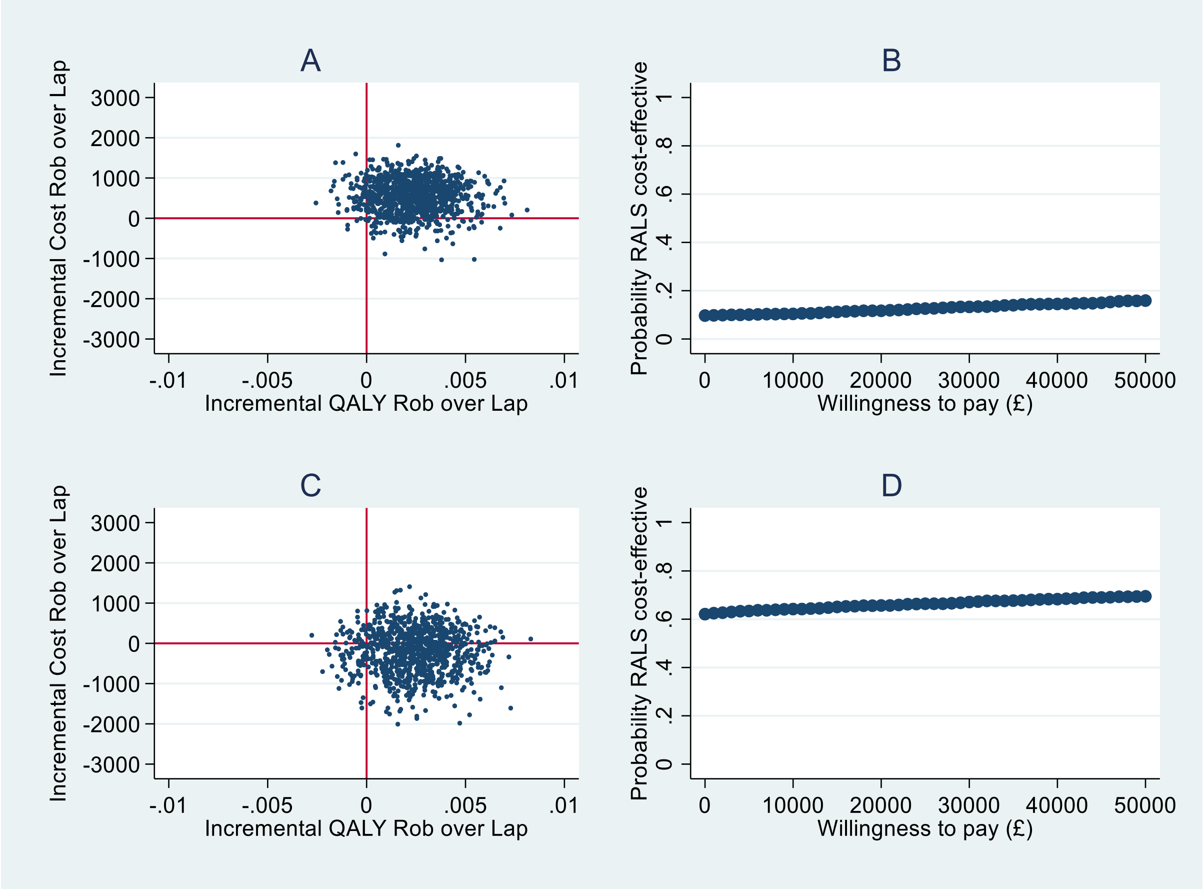

Supplemental material 1: Resource Use

Table S1.1: Resource use for surgical procedure

Resource or unit intervention	Unit	Mean usage in standard laparoscopic	Mean usage in standard robotic	Resource source
**Fixed theatre usage overheads**				
Operating theatre	Average theatre duration	168 minutes	183 minutes	RoBoQoL study procedure CRF form
**Staff**				
**Resource or unit intervention**	**Unit**	**Mean usage in standard laparoscopic**	**Mean usage in standard robotic**	**Resource source**
Consultant surgeon	Per hour	1	1	South Tees Hospitals NHS Foundation based on personal communication with Jeremy Twigg and Alison Waines between Jan-Nov 2020
Assistant surgeon (registrar)	Per hour	1	1	South Tees Hospitals NHS Foundation based on personal communication with Jeremy Twigg and Alison Waines between Jan-Nov 2020
Consultant anaesthetist	Per hour	1	1	South Tees Hospitals NHS Foundation based on personal communication with Jeremy Twigg and Alison Waines between Jan-Nov 2020
Anaesthetist registrar	Per hour	1	1	South Tees Hospitals NHS Foundation based on personal

For peer review only

				communication with Jeremy Twigg and Alison Waines between Jan-Nov 2020
Anaesthetist nurse	Per hour	1	1	South Tees Hospitals NHS Foundation based on personal communication with Jeremy Twigg and Alison Waines between Jan-Nov 2020
Nurse	Per hour	1	1	South Tees Hospitals NHS Foundation based on personal communication with Jeremy Twigg and Alison Waines between Jan-Nov 2020
Health care assistant	Per hour	1	1	South Tees Hospitals NHS Foundation based on personal communication with Jeremy Twigg and Alison Waines between Jan-Nov 2020
Recovery practitioner	Per hour	1	1	South Tees Hospitals NHS Foundation based on personal communication with Jeremy Twigg and Alison Waines between Jan-Nov 2020
**Capital equipment**				
**Resource or unit intervention**	**Unit**	**Mean usage in standard laparoscopic**	**Mean usage in standard robotic**	**Resource source**

For peer review only

Laparoscopic device (slave screens included)	Per procedure	1	1	South Tees Hospitals NHS Foundation based on personal communication with Jeremy Twigg and Alison Waines between Jan-Nov 2020
Robotic device	Per procedure	1	1	South Tees Hospitals NHS Foundation based on personal communication with Jeremy Twigg and Alison Waines between Jan-Nov 2020
Diathermy console	Per procedure	1	1	South Tees Hospitals NHS Foundation based on personal communication with Jeremy Twigg and Alison Waines between Jan-Nov 2020
Anaesthetic machine	Per procedure	1	1	South Tees Hospitals NHS Foundation based on personal communication with Jeremy Twigg and Alison Waines between Jan-Nov 2020
PCA pump	Per procedure	1	1	South Tees Hospitals NHS Foundation based on personal communication with Jeremy Twigg and Alison Waines between Jan-Nov 2020

For peer review only

Suction machine	Per procedure	1	1	South Tees Hospitals NHS Foundation based on personal communication with Jeremy Twigg and Alison Waines between Jan-Nov 2020
**Consumables**<br>**Surgical equipment specific to laparoscopic and robotic surgery**				
**Resource or unit intervention**	**Unit**	**Mean usage in standard laparoscopic**	**Mean usage in standard robotic**	**Resource source**
Gel port/trocar	Per procedure	1	n/a	South Tees Hospitals NHS Foundation based on personal communication with Jeremy Twigg and Alison Waines between Jan-Nov 2020
55mm dual port/trocar	Per procedure	4	n/a	South Tees Hospitals NHS Foundation based on personal communication with Jeremy Twigg and Alison Waines between Jan-Nov 2020
11mm port/trocar	Per procedure	1	n/a	South Tees Hospitals NHS Foundation based on personal communication with Jeremy Twigg and Alison Waines between Jan-Nov 2020
Verris lap pack	Per procedure	1	n/a	South Tees Hospitals NHS Foundation based on personal communication

For peer review only

				with Jeremy Twigg and Alison Waines between Jan-Nov 2020
Vcare manipulator	Per procedure	1	1	South Tees Hospitals NHS Foundation based on personal communication with Jeremy Twigg and Alison Waines between Jan-Nov 2020
Applied scissors	Per procedure	1	n/a	South Tees Hospitals NHS Foundation based on personal communication with Jeremy Twigg and Alison Waines between Jan-Nov 2020
Harmonic (ethicon)	Per procedure	1	n/a	South Tees Hospitals NHS Foundation based on personal communication with Jeremy Twigg and Alison Waines between Jan-Nov 2020
Misc. forceps	Per procedure	1	n/a	South Tees Hospitals NHS Foundation based on personal communication with Jeremy Twigg and Alison Waines between Jan-Nov 2020
Vlock suture	Per procedure	1	1	South Tees Hospitals NHS Foundation based on personal communication with Jeremy

For peer review only

				Twigg and Alison Waines between Jan-Nov 2020
Endoclose	Per procedure	1	1	South Tees Hospitals NHS Foundation based on personal communication with Jeremy Twigg and Alison Waines between Jan-Nov 2020
Syringes	Per procedure	1	1	South Tees Hospitals NHS Foundation based on personal communication with Jeremy Twigg and Alison Waines between Jan-Nov 2020
Scissors (robot specific)	Per procedure	1	1	South Tees Hospitals NHS Foundation based on personal communication with Jeremy Twigg and Alison Waines between Jan-Nov 2020
Pro-grasp	Per procedure	1	1	South Tees Hospitals NHS Foundation based on personal communication with Jeremy Twigg and Alison Waines between Jan-Nov 2020
Maryland bi-polar	Per procedure	1	1	South Tees Hospitals NHS Foundation based on personal communication with Jeremy Twigg and Alison

For peer review only

				Waines between Jan-Nov 2020
Needle holder	Per procedure	1	1	South Tees Hospitals NHS Foundation based on personal communication with Jeremy Twigg and Alison Waines between Jan-Nov 2020
Scope sterilisation for 30 degrees & 0 degrees	Per procedure	1	1	South Tees Hospitals NHS Foundation based on personal communication with Jeremy Twigg and Alison Waines between Jan-Nov 2020
Accessory set sterilisation	Per procedure	n/a	2	South Tees Hospitals NHS Foundation based on personal communication with Jeremy Twigg and Alison Waines between Jan-Nov 2020
**General consumable surgical equipment**				
**Resource or unit intervention**	**Unit**	**Mean usage in standard laparoscopic**	**Mean usage in standard robotic**	**Resource source**
Arterial line	Per procedure	1	1	South Tees Hospitals NHS Foundation based on personal communication with Jeremy Twigg, Alison Waines and Richard Bickle between Jan-Nov 2020
Central line	Per procedure	1	1	South Tees Hospitals NHS

For peer review only

				Foundation based on personal communication with Jeremy Twigg, Alison Waines and Richard Bickle between Jan-Nov 2020
12F catheter	Per procedure	1	1	South Tees Hospitals NHS Foundation based on personal communication with Jeremy Twigg, Alison Waines and Richard Bickle between Jan-Nov 2020
Urine drainage bag	Per procedure	1	1	South Tees Hospitals NHS Foundation based on personal communication with Jeremy Twigg, Alison Waines and Richard Bickle between Jan-Nov 2020
Monocryl	Per procedure	4	4	South Tees Hospitals NHS Foundation based on personal communication with Jeremy Twigg, Alison Waines and Richard Bickle between Jan-Nov 2020
Bair Hugger warmer	Per procedure	1	1	South Tees Hospitals NHS Foundation based on personal

For peer review only

				communication with Jeremy Twigg, Alison Waines and Richard Bickle between Jan-Nov 2020
Fluid warmer	Per procedure	1	1	South Tees Hospitals NHS Foundation based on personal communication with Jeremy Twigg, Alison Waines and Richard Bickle between Jan-Nov 2020
Drape	Per procedure	1	n/a	South Tees Hospitals NHS Foundation based on personal communication with Jeremy Twigg, Alison Waines and Richard Bickle between Jan-Nov 2020
4 arm drape	Per procedure	n/a	1	South Tees Hospitals NHS Foundation based on personal communication with Jeremy Twigg, Alison Waines and Richard Bickle between Jan-Nov 2020
**Scissor tip cover**	Per procedure	n/a	1	South Tees Hospitals NHS Foundation based on personal communication with Jeremy Twigg, Alison

For peer review only

				Waines and Richard Bickle between Jan-Nov 2020
**Blood products**				
**Resource or unit intervention**	**Unit**	**Mean usage in standard laparoscopic**	**Mean usage in standard robotic**	**Resource source**
Standard red blood cells	Per procedure	0.06 units	0.07 units	RoBoQoL study procedure CRF form
**Other resources**				
Anaesthetic equipment	Per procedure	1	1	South Tees Hospitals NHS Foundation based on personal communication with Richard Bickle between Jan-Nov 2020
Anaesthetic drugs	Per procedure	1	1	South Tees Hospitals NHS Foundation based on personal communication with Richard Bickle between Jan-Nov 2020
IV fluids	Per procedure	5	5	South Tees Hospitals NHS Foundation based on personal communication with Richard Bickle between Jan-Nov 2020
**Type of stay**				
ICU	Per day	0.07	0.08	RoBoQoL study procedure CRF
Ward	Per day	2.22	1.28	RoBoQoL study procedure CRF

Table S1.2: Resource use during follow-up

For peer review only

Resource or unit intervention		measure	RALS (n=159)	CLS (n=73)	Source
Week 1					
**Primary Care**					
General Practitioner (GP)	Home Visit	Mean (SD)	0.01 (0.09)	0 (0)	RoboQoL CRF
		Missing (N%)	39 (24.5)	6 (9)	
	Surgery Visit	Mean (SD)	0.13 (0.4)	0.05 (0.21)	RoboQoL CRF
		Missing (N%)	41 (25.8)	7 (10.6)	
	Phone Appointment	Mean (SD)	0.08 (0.3)	0.09 (0.29)	RoboQoL CRF
		Missing (N%)	40 (25.2)	6 (9)	
Nurse	Home Visit	Mean (SD)	0.16 (0.96)	0 (0)	RoboQoL CRF
		Missing (N%)	40 (25.2)	6 (9)	
	Surgery Visit	Mean (SD)	0.05 (0.22)	0.04 (0.27)	RoboQoL CRF
		Missing (N%)	41 (25.8)	6 (9)	
	Phone Appointment	Mean (SD)	0.03 (0.16)	0.03 (0.17)	RoboQoL CRF
		Missing (N%)	39 (24.5)	6 (9)	
Occupational Therapist (OT)	Home Visit	Mean (SD)	0 (0)	0 (0)	RoboQoL CRF
		Missing (N%)	39 (24.5)	7 (10.6)	
	Surgery Visit	Mean (SD)	0 (0)	0.05 (0.37)	RoboQoL CRF
		Missing (N%)	39 (24.5)	7 (10.6)	
	Hospital Visit	Mean (SD)	0 (0)	0 (0)	RoboQoL CRF
		Missing (N%)	39 (24.5)	7 (10.6)	
Physiotherapist	Home Visit	Mean (SD)	0 (0)	0 (0)	RoboQoL CRF
		Missing (N%)	39 (24.5)	6 (9)	
	Surgery Visit	Mean (SD)	39 (0)	6 (0)	RoboQoL CRF
		Missing (N%)	39 (24.5)	6 (9)	
	Phone Appointment	Mean (SD)	0.1 (0.4)	0.22 (0.42)	RoboQoL CRF
		Missing (N%)	39 (24.5)	6 (9)	
	Hospital Visit	Mean (SD)	0 (0)	0 (0)	RoboQoL CRF
		Missing (N%)	39 (24.5)	6 (9)	
	Day Visit	Mean (SD)	0 (0)	0 (0)	RoboQoL CRF
		Missing (N%)	39 (24.5)	6 (9)	
**Secondary Care**					
Outpatient Appointment		Mean (SD)	0.1 (0.1)	0.07 (0.07)	RoboQoL CRF
		Missing (N%)	39 (24.5)	6 (9)	
Ambulance See and treat		Mean (SD)	0.01 (0.09)	0 (0)	RoboQoL CRF
		Missing (N%)	39 (24.5)	6 (9)	
Ambulance see and convey		Mean (SD)	0 (0)	0 (0)	RoboQoL CRF
		Missing (N%)	39 (24.5)	6 (9)	
A&E		Mean (SD)	0.08 (0.66)	0.03 (0.17)	RoboQoL CRF
		Missing (N%)	41 (25.8)	6 (9)	
Hospital Ward Day		Mean (SD)	0.05 (0.26)	0.04 (0.21)	RoboQoL CRF
		Missing (N%)	41 (25.8)	6 (9)	
Hospital Overnight Visit		Mean (SD)	0.15 (0.65)	0.58 (1.55)	RoboQoL CRF
		Missing (N%)	40 (25.2)	6 (9)	

For peer review only

Week 2					
**Primary Care**					
General Practitioner (GP)	Home Visit	Mean (SD)	0.02 (0.18)	0 (0)	RoboQoL CRF
		Missing (N%)	42 (26.4)	3 (4.3)	
	Surgery Visit	Mean (SD)	0.07 (0.25)	0.04 (0.2)	RoboQoL CRF
		Missing (N%)	42 (26.4)	3 (4.3)	
	Phone Appointment	Mean (SD)	0.09 (0.35)	0.07 (0.26)	RoboQoL CRF
		Missing (N%)	42 (26.4)	3 (4.3)	
Nurse	Home Visit	Mean (SD)	0.14 (0.93)	0.01 (0.12)	RoboQoL CRF
		Missing (N%)	43 (27)	4 (5.8)	
	Surgery Visit	Mean (SD)	0.1 (0.31)	0.01 (0.12)	RoboQoL CRF
		Missing (N%)	43 (27)	6 (9)	
	Phone Appointment	Mean (SD)	0.03 (0.21)	0 (0)	RoboQoL CRF
		Missing (N%)	43 (27)	4 (5.8)	
Occupational Therapist (OT)	Home Visit	Mean (SD)	0 (0)	0 (0)	RoboQoL CRF
		Missing (N%)	43 (27)	4 (5.8)	
	Surgery Visit	Mean (SD)	0 (0)	0 (0)	RoboQoL CRF
		Missing (N%)	44 (27.7)	4 (5.8)	
	Hospital Visit	Mean (SD)	0 (0)	0 (0)	RoboQoL CRF
		Missing (N%)	43 (27)	4 (5.8)	
Physiotherapist	Home Visit	Mean (SD)	0 (0)	0 (0)	RoboQoL CRF
		Missing (N%)	42 (26.4)	4 (5.8)	
	Surgery Visit	Mean (SD)	42 (0)	4 (0)	RoboQoL CRF
		Missing (N%)	42 (26.4)	4 (5.8)	
	Phone Appointment	Mean (SD)	0 (0)	0.03 (0.17)	RoboQoL CRF
		Missing (N%)	42 (26.4)	4 (5.8)	
	Hospital Visit	Mean (SD)	0 (0)	0 (0)	RoboQoL CRF
		Missing (N%)	42 (26.4)	4 (5.8)	
	Day Visit	Mean (SD)	0 (0)	0 (0)	RoboQoL CRF
		Missing (N%)	42 (26.4)	4 (5.8)	
**Secondary Care**					
Outpatient Appointment		Mean (SD)	0.21 (0.21)	0.12 (0.12)	RoboQoL CRF
		Missing (N%)	39 (24.5)	4 (5.8)	
Ambulance See and treat		Mean (SD)	0.01 (0.09)	0.03 (0.17)	RoboQoL CRF
		Missing (N%)	42 (26.4)	3 (4.3)	
Ambulance see and convey		Mean (SD)	0.01 (0.09)	0.03 (0.17)	RoboQoL CRF
		Missing (N%)	42 (26.4)	3 (4.3)	
A&E		Mean (SD)	0.03 (0.18)	0.03 (0.17)	RoboQoL CRF
		Missing (N%)	42 (26.4)	3 (4.3)	
Hospital Ward Day		Mean (SD)	0.03 (0.16)	0.01 (0.12)	RoboQoL CRF
		Missing (N%)	42 (26.4)	4 (5.8)	
Hospital Overnight Visit		Mean (SD)	0.12 (0.71)	0.34 (1.38)	RoboQoL CRF
		Missing (N%)	42 (26.4)	3 (4.3)	
Week 3					
**Primary Care**					

For peer review only

General Practitioner (GP)	Home Visit	Mean (SD)	0 (0)	0 (0)	RoboQoL CRF
		Missing (N%)	44 (27.7)	11 (17.7)	
	Surgery Visit	Mean (SD)	0.11 (0.35)	0.06 (0.25)	RoboQoL CRF
		Missing (N%)	45 (28.3)	11 (17.7)	
	Phone Appointment	Mean (SD)	0.07 (0.29)	0.03 (0.18)	RoboQoL CRF
		Missing (N%)	44 (27.7)	11 (17.7)	
Nurse	Home Visit	Mean (SD)	0.14 (0.94)	0 (0)	RoboQoL CRF
		Missing (N%)	44 (27.7)	11 (17.7)	
	Surgery Visit	Mean (SD)	0.09 (0.34)	0.05 (0.22)	RoboQoL CRF
		Missing (N%)	44 (27.7)	11 (17.7)	
	Phone Appointment	Mean (SD)	0.03 (0.16)	0.03 (0.18)	RoboQoL CRF
		Missing (N%)	44 (27.7)	11 (17.7)	
Occupational Therapist (OT)	Home Visit	Mean (SD)	0 (0)	0 (0)	RoboQoL CRF
		Missing (N%)	44 (27.7)	11 (17.7)	
	Surgery Visit	Mean (SD)	0 (0)	0.03 (0.25)	RoboQoL CRF
		Missing (N%)	44 (27.7)	11 (17.7)	
	Hospital Visit	Mean (SD)	0 (0)	0 (0)	RoboQoL CRF
		Missing (N%)	44 (27.7)	11 (17.7)	
Physiotherapist	Home Visit	Mean (SD)	0 (0)	0 (0)	RoboQoL CRF
		Missing (N%)	45 (28.3)	11 (17.7)	
	Surgery Visit	Mean (SD)	45 (0)	11 (0)	RoboQoL CRF
		Missing (N%)	45 (28.3)	11 (17.7)	
	Phone Appointment	Mean (SD)	0.02 (0.13)	0 (0)	RoboQoL CRF
		Missing (N%)	45 (28.3)	11 (17.7)	
	Hospital Visit	Mean (SD)	0.01 (0.09)	0 (0)	RoboQoL CRF
		Missing (N%)	45 (28.3)	11 (17.7)	
	Day Visit	Mean (SD)	0 (0)	0 (0)	RoboQoL CRF
		Missing (N%)	45 (28.3)	11 (17.7)	
**Secondary Care**					
Outpatient Appointment		Mean (SD)	0.3 (0.3)	0.19 (0.19)	RoboQoL CRF
		Missing (N%)	43 (27)	10 (15.9)	
Ambulance See and treat		Mean (SD)	0 (0)	0 (0)	RoboQoL CRF
		Missing (N%)	45 (28.3)	11 (17.7)	
Ambulance see and convey		Mean (SD)	0 (0)	0 (0)	RoboQoL CRF
		Missing (N%)	45 (28.3)	11 (17.7)	
A&E		Mean (SD)	0.01 (0.09)	0 (0)	RoboQoL CRF
		Missing (N%)	45 (28.3)	11 (17.7)	
Hospital Ward Day		Mean (SD)	0 (0)	0.02 (0.13)	RoboQoL CRF
		Missing (N%)	45 (28.3)	11 (17.7)	
Hospital Overnight Visit		Mean (SD)	0.05 (0.26)	0.16 (1.15)	RoboQoL CRF
		Missing (N%)	45 (28.3)	11 (17.7)	
Week 4					
**Primary Care**					
General Practitioner (GP)	Home Visit	Mean (SD)	0.01 (0.09)	0.02 (0.13)	RoboQoL CRF
		Missing (N%)	44 (27.7)	10 (15.9)	

For peer review only

	Surgery Visit	Mean (SD)	0.08 (0.27)	0.1 (0.35)	RoboQoL CRF
		Missing (N%)	46 (28.9)	10 (15.9)	
	Phone Appointment	Mean (SD)	0.04 (0.2)	0.03 (0.25)	RoboQoL CRF
		Missing (N%)	44 (27.7)	10 (15.9)	
Nurse	Home Visit	Mean (SD)	0.09 (0.66)	0 (0)	RoboQoL CRF
		Missing (N%)	46 (28.9)	10 (15.9)	
	Surgery Visit	Mean (SD)	0.04 (0.25)	0.06 (0.25)	RoboQoL CRF
		Missing (N%)	46 (28.9)	10 (15.9)	
	Phone Appointment	Mean (SD)	0 (0)	0 (0)	RoboQoL CRF
		Missing (N%)	46 (28.9)	10 (15.9)	
Occupational Therapist (OT)	Home Visit	Mean (SD)	0 (0)	0 (0)	RoboQoL CRF
		Missing (N%)	45 (28.3)	10 (15.9)	
	Surgery Visit	Mean (SD)	0.01 (0.09)	0 (0)	RoboQoL CRF
		Missing (N%)	45 (28.3)	10 (15.9)	
	Hospital Visit	Mean (SD)	0 (0)	0 (0)	RoboQoL CRF
		Missing (N%)	45 (28.3)	10 (15.9)	
Physiotherapist	Home Visit	Mean (SD)	0 (0)	0 (0)	RoboQoL CRF
		Missing (N%)	47 (29.6)	10 (15.9)	
	Surgery Visit	Mean (SD)	47 (0)	10 (0)	RoboQoL CRF
		Missing (N%)	47 (29.6)	10 (15.9)	
	Phone Appointment	Mean (SD)	0 (0)	0 (0)	RoboQoL CRF
		Missing (N%)	48 (30.2)	10 (15.9)	
	Hospital Visit	Mean (SD)	0.01 (0.09)	0 (0)	RoboQoL CRF
		Missing (N%)	47 (29.6)	10 (15.9)	
	Day Visit	Mean (SD)	0 (0)	0 (0)	RoboQoL CRF
		Missing (N%)	47 (29.6)	10 (15.9)	
**Secondary Care**					
Outpatient Appointment		Mean (SD)	0.34 (0.34)	0.11 (0.11)	RoboQoL CRF
		Missing (N%)	46 (28.9)	8 (12.3)	
Ambulance See and treat		Mean (SD)	0.01 (0.09)	0 (0)	RoboQoL CRF
		Missing (N%)	45 (28.3)	9 (14.1)	
Ambulance see and convey		Mean (SD)	0 (0)	0 (0)	RoboQoL CRF
		Missing (N%)	45 (28.3)	9 (14.1)	
A&E		Mean (SD)	0.01 (0.09)	0 (0)	RoboQoL CRF
		Missing (N%)	45 (28.3)	9 (14.1)	
Hospital Ward Day		Mean (SD)	0 (0)	0.02 (0.13)	RoboQoL CRF
		Missing (N%)	45 (28.3)	9 (14.1)	
Hospital Overnight Visit		Mean (SD)	0.01 (0.09)	0.02 (0.13)	RoboQoL CRF
		Missing (N%)	45 (28.3)	9 (14.1)	
Week 5					
**Primary Care**					
General Practitioner (GP)	Home Visit	Mean (SD)	0 (0)	0 (0)	RoboQoL CRF
		Missing (N%)	56 (35.2)	14 (23.7)	
	Surgery Visit	Mean (SD)	0.1 (0.33)	0.07 (0.25)	RoboQoL CRF
		Missing (N%)	57 (35.8)	14 (23.7)	

For peer review only

	Phone Appointment	Mean (SD)	0.04 (0.2)	0.05 (0.22)	RoboQoL CRF
		Missing (N%)	57 (35.8)	14 (23.7)	
Nurse	Home Visit	Mean (SD)	0 (0)	0 (0)	RoboQoL CRF
		Missing (N%)	57 (35.8)	14 (23.7)	
	Surgery Visit	Mean (SD)	0.09 (0.32)	0.08 (0.28)	RoboQoL CRF
		Missing (N%)	57 (35.8)	14 (23.7)	
	Phone Appointment	Mean (SD)	0.01 (0.1)	0 (0)	RoboQoL CRF
		Missing (N%)	57 (35.8)	14 (23.7)	
Occupational Therapist (OT)	Home Visit	Mean (SD)	0 (0)	0 (0)	RoboQoL CRF
		Missing (N%)	57 (35.8)	15 (25.9)	
	Surgery Visit	Mean (SD)	0 (0)	0 (0)	RoboQoL CRF
		Missing (N%)	57 (35.8)	15 (25.9)	
	Hospital Visit	Mean (SD)	0 (0)	0 (0)	RoboQoL CRF
		Missing (N%)	57 (35.8)	15 (25.9)	
Physiotherapist	Home Visit	Mean (SD)	0 (0)	0 (0)	RoboQoL CRF
		Missing (N%)	56 (35.2)	15 (25.9)	
	Surgery Visit	Mean (SD)	56 (0)	15 (0)	RoboQoL CRF
		Missing (N%)	56 (35.2)	15 (25.9)	
	Phone Appointment	Mean (SD)	0 (0)	0 (0)	RoboQoL CRF
		Missing (N%)	56 (35.2)	15 (25.9)	
	Hospital Visit	Mean (SD)	0 (0)	0 (0)	RoboQoL CRF
		Missing (N%)	56 (35.2)	15 (25.9)	
	Day Visit	Mean (SD)	0 (0)	0 (0)	RoboQoL CRF
		Missing (N%)	56 (35.2)	15 (25.9)	
**Secondary Care**					
Outpatient Appointment		Mean (SD)	0.26 (0.26)	0.1 (0.1)	RoboQoL CRF
		Missing (N%)	57 (35.8)	14 (23.7)	
Ambulance See and treat		Mean (SD)	0 (0)	0 (0)	RoboQoL CRF
		Missing (N%)	59 (37.1)	14 (23.7)	
Ambulance see and convey		Mean (SD)	0 (0)	0 (0)	RoboQoL CRF
		Missing (N%)	59 (37.1)	14 (23.7)	
A&E		Mean (SD)	0.02 (0.14)	0 (0)	RoboQoL CRF
		Missing (N%)	56 (35.2)	14 (23.7)	
Hospital Ward Day		Mean (SD)	0 (0)	0.02 (0.13)	RoboQoL CRF
		Missing (N%)	56 (35.2)	14 (23.7)	
Hospital Overnight Visit		Mean (SD)	0 (0)	0.02 (0.13)	RoboQoL CRF
		Missing (N%)	56 (35.2)	14 (23.7)	
Week 6					
**Primary Care**					
General Practitioner (GP)	Home Visit	Mean (SD)	0 (0)	0.02 (0.13)	RoboQoL CRF
		Missing (N%)	55 (34.6)	14 (23.7)	
	Surgery Visit	Mean (SD)	0.16 (0.37)	0.09 (0.29)	RoboQoL CRF
		Missing (N%)	55 (34.6)	16 (28.1)	
	Phone Appointment	Mean (SD)	0.04 (0.19)	0.03 (0.18)	RoboQoL CRF
		Missing (N%)	55 (34.6)	13 (21.7)	

For peer review only

Nurse	Home Visit	Mean (SD)	0 (0)	0.03 (0.18)	RoboQoL CRF
		Missing (N%)	55 (34.6)	13 (21.7)	
	Surgery Visit	Mean (SD)	0.01 (0.1)	0.03 (0.18)	RoboQoL CRF
		Missing (N%)	55 (34.6)	14 (23.7)	
	Phone Appointment	Mean (SD)	0 (0)	0.05 (0.22)	RoboQoL CRF
		Missing (N%)	55 (34.6)	13 (21.7)	
Occupational Therapist (OT)	Home Visit	Mean (SD)	0 (0)	0 (0)	RoboQoL CRF
		Missing (N%)	55 (34.6)	13 (21.7)	
	Surgery Visit	Mean (SD)	0 (0)	0 (0)	RoboQoL CRF
		Missing (N%)	55 (34.6)	13 (21.7)	
	Hospital Visit	Mean (SD)	0 (0)	0 (0)	RoboQoL CRF
		Missing (N%)	55 (34.6)	13 (21.7)	
Physiotherapist	Home Visit	Mean (SD)	0 (0)	0 (0)	RoboQoL CRF
		Missing (N%)	55 (34.6)	13 (21.7)	
	Surgery Visit	Mean (SD)	55 (0)	13 (0)	RoboQoL CRF
		Missing (N%)	55 (34.6)	13 (21.7)	
	Phone Appointment	Mean (SD)	0 (0)	0 (0)	RoboQoL CRF
		Missing (N%)	55 (34.6)	13 (21.7)	
	Hospital Visit	Mean (SD)	0.01 (0.1)	0 (0)	RoboQoL CRF
		Missing (N%)	55 (34.6)	13 (21.7)	
	Day Visit	Mean (SD)	0 (0)	0 (0)	RoboQoL CRF
		Missing (N%)	55 (34.6)	13 (21.7)	
**Secondary Care**					
Outpatient Appointment		Mean (SD)	0.31 (0.31)	0.12 (0.12)	RoboQoL CRF
		Missing (N%)	55 (34.6)	13 (21.7)	
Ambulance See and treat		Mean (SD)	0 (0)	0 (0)	RoboQoL CRF
		Missing (N%)	55 (34.6)	13 (21.7)	
Ambulance see and convey		Mean (SD)	0 (0)	0 (0)	RoboQoL CRF
		Missing (N%)	55 (34.6)	13 (21.7)	
A&E		Mean (SD)	0 (0)	0.02 (0.13)	RoboQoL CRF
		Missing (N%)	56 (35.2)	13 (21.7)	
Hospital Ward Day		Mean (SD)	0 (0)	0.02 (0.13)	RoboQoL CRF
		Missing (N%)	56 (35.2)	13 (21.7)	
Hospital Overnight Visit		Mean (SD)	0 (0)	0.05 (0.39)	RoboQoL CRF
		Missing (N%)	56 (35.2)	13 (21.7)	
Week 7					
**Primary Care**					
General Practitioner (GP)	Home Visit	Mean (SD)	0 (0)	0.03 (0.18)	RoboQoL CRF
		Missing (N%)	54 (34)	13 (21.7)	
	Surgery Visit	Mean (SD)	0.08 (0.3)	0.09 (0.28)	RoboQoL CRF
		Missing (N%)	55 (34.6)	15 (25.9)	
	Phone Appointment	Mean (SD)	0.05 (0.26)	0.02 (0.13)	RoboQoL CRF
		Missing (N%)	56 (35.2)	13 (21.7)	
Nurse	Home Visit	Mean (SD)	0 (0)	0.03 (0.18)	RoboQoL CRF
		Missing (N%)	56 (35.2)	13 (21.7)	

For peer review only

	Surgery Visit	Mean (SD)	0.01 (0.1)	0.05 (0.22)	RoboQoL CRF
		Missing (N%)	57 (35.8)	15 (25.9)	
	Phone Appointment	Mean (SD)	0.01 (0.1)	0.02 (0.13)	RoboQoL CRF
		Missing (N%)	57 (35.8)	13 (21.7)	
Occupational Therapist (OT)	Home Visit	Mean (SD)	0 (0)	0 (0)	RoboQoL CRF
		Missing (N%)	57 (35.8)	13 (21.7)	
	Surgery Visit	Mean (SD)	0 (0)	0 (0)	RoboQoL CRF
		Missing (N%)	58 (36.5)	13 (21.7)	
	Hospital Visit	Mean (SD)	0 (0)	0 (0)	RoboQoL CRF
		Missing (N%)	58 (36.5)	13 (21.7)	
Physiotherapist	Home Visit	Mean (SD)	0 (0)	0 (0)	RoboQoL CRF
		Missing (N%)	55 (34.6)	13 (21.7)	
	Surgery Visit	Mean (SD)	55 (0)	13 (0)	RoboQoL CRF
		Missing (N%)	55 (34.6)	13 (21.7)	
	Phone Appointment	Mean (SD)	0 (0)	0 (0)	RoboQoL CRF
		Missing (N%)	55 (34.6)	13 (21.7)	
	Hospital Visit	Mean (SD)	0 (0)	0 (0)	RoboQoL CRF
		Missing (N%)	55 (34.6)	13 (21.7)	
	Day Visit	Mean (SD)	0 (0)	0 (0)	RoboQoL CRF
		Missing (N%)	55 (34.6)	13 (21.7)	
**Secondary Care**					
Outpatient Appointment		Mean (SD)	0.5 (0.5)	0.28 (0.28)	RoboQoL CRF
		Missing (N%)	56 (35.2)	13 (21.7)	
Ambulance See and treat		Mean (SD)	0 (0)	0 (0)	RoboQoL CRF
		Missing (N%)	55 (34.6)	13 (21.7)	
Ambulance see and convey		Mean (SD)	0 (0)	0 (0)	RoboQoL CRF
		Missing (N%)	55 (34.6)	13 (21.7)	
A&E		Mean (SD)	0 (0)	0 (0)	RoboQoL CRF
		Missing (N%)	56 (35.2)	13 (21.7)	
Hospital Ward Day		Mean (SD)	0.03 (0.17)	0 (0)	RoboQoL CRF
		Missing (N%)	56 (35.2)	13 (21.7)	
Hospital Overnight Visit		Mean (SD)	0 (0)	0 (0)	RoboQoL CRF
		Missing (N%)	56 (35.2)	13 (21.7)	
Week 8					
**Primary Care**					
General Practitioner (GP)	Home Visit	Mean (SD)	0 (0)	0.03 (0.18)	RoboQoL CRF
		Missing (N%)	57 (35.8)	13 (21.7)	
	Surgery Visit	Mean (SD)	0.08 (0.3)	0.16 (0.37)	RoboQoL CRF
		Missing (N%)	57 (35.8)	15 (25.9)	
	Phone Appointment	Mean (SD)	0.01 (0.1)	0.02 (0.13)	RoboQoL CRF
		Missing (N%)	58 (36.5)	13 (21.7)	
Nurse	Home Visit	Mean (SD)	0 (0)	0.02 (0.13)	RoboQoL CRF
		Missing (N%)	55 (34.6)	14 (23.7)	
	Surgery Visit	Mean (SD)	0.03 (0.17)	0.1 (0.31)	RoboQoL CRF
		Missing (N%)	57 (35.8)	15 (25.9)	

For peer review only

	Phone Appointment	Mean (SD)	0.02 (0.14)	0 (0)	RoboQoL CRF
		Missing (N%)	55 (34.6)	14 (23.7)	
Occupational Therapist (OT)	Home Visit	Mean (SD)	0 (0)	0 (0)	RoboQoL CRF
		Missing (N%)	56 (35.2)	14 (23.7)	
	Surgery Visit	Mean (SD)	0 (0)	0 (0)	RoboQoL CRF
		Missing (N%)	56 (35.2)	14 (23.7)	
	Hospital Visit	Mean (SD)	0 (0)	0 (0)	RoboQoL CRF
		Missing (N%)	56 (35.2)	14 (23.7)	
Physiotherapist	Home Visit	Mean (SD)	0 (0)	0 (0)	RoboQoL CRF
		Missing (N%)	57 (35.8)	14 (23.7)	
	Surgery Visit	Mean (SD)	57 (0)	14 (0)	RoboQoL CRF
		Missing (N%)	57 (35.8)	14 (23.7)	
	Phone Appointment	Mean (SD)	0 (0)	0 (0)	RoboQoL CRF
		Missing (N%)	57 (35.8)	14 (23.7)	
	Hospital Visit	Mean (SD)	0 (0)	0 (0)	RoboQoL CRF
		Missing (N%)	57 (35.8)	14 (23.7)	
	Day Visit	Mean (SD)	0 (0)	0 (0)	RoboQoL CRF
		Missing (N%)	57 (35.8)	14 (23.7)	
**Secondary Care**					
Outpatient Appointment		Mean (SD)	0.55 (0.55)	0.2 (0.2)	RoboQoL CRF
		Missing (N%)	56 (35.2)	13 (21.7)	
Ambulance See and treat		Mean (SD)	0 (0)	0 (0)	RoboQoL CRF
		Missing (N%)	57 (35.8)	14 (23.7)	
Ambulance see and convey		Mean (SD)	0 (0)	0 (0)	RoboQoL CRF
		Missing (N%)	57 (35.8)	14 (23.7)	
A&E		Mean (SD)	0 (0)	0 (0)	RoboQoL CRF
		Missing (N%)	57 (35.8)	14 (23.7)	
Hospital Ward Day		Mean (SD)	0.01 (0.1)	0 (0)	RoboQoL CRF
		Missing (N%)	57 (35.8)	14 (23.7)	
Hospital Overnight Visit		Mean (SD)	0.02 (0.2)	0 (0)	RoboQoL CRF
		Missing (N%)	57 (35.8)	14 (23.7)	
Week 9					
**Primary Care**					
General Practitioner (GP)	Home Visit	Mean (SD)	0 (0)	0 (0)	RoboQoL CRF
		Missing (N%)	55 (34.6)	13 (21.7)	
	Surgery Visit	Mean (SD)	0.08 (0.27)	0.1 (0.3)	RoboQoL CRF
		Missing (N%)	56 (35.2)	13 (21.7)	
	Phone Appointment	Mean (SD)	0.02 (0.14)	0 (0)	RoboQoL CRF
		Missing (N%)	55 (34.6)	13 (21.7)	
Nurse	Home Visit	Mean (SD)	0.01 (0.1)	0 (0)	RoboQoL CRF
		Missing (N%)	55 (34.6)	13 (21.7)	
	Surgery Visit	Mean (SD)	0.02 (0.14)	0.07 (0.25)	RoboQoL CRF
		Missing (N%)	55 (34.6)	13 (21.7)	
	Phone Appointment	Mean (SD)	0.01 (0.1)	0 (0)	RoboQoL CRF
		Missing (N%)	55 (34.6)	13 (21.7)	

For peer review only

Occupational Therapist (OT)	Home Visit	Mean (SD)	0 (0)	0 (0)	RoboQoL CRF
		Missing (N%)	56 (35.2)	13 (21.7)	
	Surgery Visit	Mean (SD)	0 (0)	0 (0)	RoboQoL CRF
		Missing (N%)	56 (35.2)	13 (21.7)	
	Hospital Visit	Mean (SD)	0 (0)	0 (0)	RoboQoL CRF
		Missing (N%)	56 (35.2)	13 (21.7)	
Physiotherapist	Home Visit	Mean (SD)	0 (0)	0 (0)	RoboQoL CRF
		Missing (N%)	56 (35.2)	13 (21.7)	
	Surgery Visit	Mean (SD)	56 (0)	13 (0)	RoboQoL CRF
		Missing (N%)	56 (35.2)	13 (21.7)	
	Phone Appointment	Mean (SD)	0 (0)	0 (0)	RoboQoL CRF
		Missing (N%)	56 (35.2)	13 (21.7)	
	Hospital Visit	Mean (SD)	0 (0)	0 (0)	RoboQoL CRF
		Missing (N%)	56 (35.2)	13 (21.7)	
	Day Visit	Mean (SD)	0 (0)	0 (0)	RoboQoL CRF
		Missing (N%)	56 (35.2)	13 (21.7)	
**Secondary Care**					
Outpatient Appointment		Mean (SD)	0.51 (0.51)	0.33 (0.33)	RoboQoL CRF
		Missing (N%)	57 (35.8)	13 (21.7)	
Ambulance See and treat		Mean (SD)	0 (0)	0 (0)	RoboQoL CRF
		Missing (N%)	56 (35.2)	14 (23.7)	
Ambulance see and convey		Mean (SD)	0 (0)	0 (0)	RoboQoL CRF
		Missing (N%)	56 (35.2)	14 (23.7)	
A&E		Mean (SD)	0 (0)	0 (0)	RoboQoL CRF
		Missing (N%)	56 (35.2)	13 (21.7)	
Hospital Ward Day		Mean (SD)	0 (0)	0.08 (0.65)	RoboQoL CRF
		Missing (N%)	56 (35.2)	13 (21.7)	
Hospital Overnight Visit		Mean (SD)	0.01 (0.1)	0.02 (0.13)	RoboQoL CRF
		Missing (N%)	56 (35.2)	13 (21.7)	
Week 10					
**Primary Care**					
General Practitioner (GP)	Home Visit	Mean (SD)	0 (0)	0 (0)	RoboQoL CRF
		Missing (N%)	56 (35.2)	13 (21.7)	
	Surgery Visit	Mean (SD)	0.06 (0.24)	0.07 (0.25)	RoboQoL CRF
		Missing (N%)	56 (35.2)	13 (21.7)	
	Phone Appointment	Mean (SD)	0.03 (0.17)	0 (0)	RoboQoL CRF
		Missing (N%)	56 (35.2)	13 (21.7)	
Nurse	Home Visit	Mean (SD)	0 (0)	0 (0)	RoboQoL CRF
		Missing (N%)	57 (35.8)	13 (21.7)	
	Surgery Visit	Mean (SD)	0.02 (0.14)	0.08 (0.28)	RoboQoL CRF
		Missing (N%)	57 (35.8)	13 (21.7)	
	Phone Appointment	Mean (SD)	0.03 (0.17)	0.02 (0.13)	RoboQoL CRF
		Missing (N%)	57 (35.8)	13 (21.7)	
Occupational Therapist (OT)	Home Visit	Mean (SD)	0 (0)	0 (0)	RoboQoL CRF
		Missing (N%)	57 (35.8)	13 (21.7)	

For peer review only

	Surgery Visit	Mean (SD)	0 (0)	0 (0)	RoboQoL CRF
		Missing (N%)	57 (35.8)	13 (21.7)	
	Hospital Visit	Mean (SD)	0 (0)	0 (0)	RoboQoL CRF
		Missing (N%)	57 (35.8)	13 (21.7)	
Physiotherapist	Home Visit	Mean (SD)	0 (0)	0.03 (0.26)	RoboQoL CRF
		Missing (N%)	57 (35.8)	13 (21.7)	
	Surgery Visit	Mean (SD)	57 (0)	13 (0)	RoboQoL CRF
		Missing (N%)	57 (35.8)	13 (21.7)	
	Phone Appointment	Mean (SD)	0.04 (0.31)	0 (0)	RoboQoL CRF
		Missing (N%)	57 (35.8)	13 (21.7)	
	Hospital Visit	Mean (SD)	0 (0)	0 (0)	RoboQoL CRF
		Missing (N%)	57 (35.8)	13 (21.7)	
	Day Visit	Mean (SD)	0 (0)	0 (0)	RoboQoL CRF
		Missing (N%)	57 (35.8)	13 (21.7)	
**Secondary Care**					
Outpatient Appointment		Mean (SD)	0.58 (0.58)	0.12 (0.12)	RoboQoL CRF
		Missing (N%)	58 (36.5)	13 (21.7)	
Ambulance See and treat		Mean (SD)	0 (0)	0.02 (0.13)	RoboQoL CRF
		Missing (N%)	57 (35.8)	13 (21.7)	
Ambulance see and convey		Mean (SD)	0 (0)	0.02 (0.13)	RoboQoL CRF
		Missing (N%)	57 (35.8)	13 (21.7)	
A&E		Mean (SD)	0.01 (0.1)	0.02 (0.13)	RoboQoL CRF
		Missing (N%)	57 (35.8)	14 (23.7)	
Hospital Ward Day		Mean (SD)	0.03 (0.17)	0.1 (0.66)	RoboQoL CRF
		Missing (N%)	57 (35.8)	13 (21.7)	
Hospital Overnight Visit		Mean (SD)	0 (0)	0.05 (0.39)	RoboQoL CRF
		Missing (N%)	57 (35.8)	13 (21.7)	
Week 11					
**Primary Care**					
General Practitioner (GP)	Home Visit	Mean (SD)	0 (0)	0 (0)	RoboQoL CRF
		Missing (N%)	56 (35.2)	13 (21.7)	
	Surgery Visit	Mean (SD)	0.06 (0.27)	0.1 (0.3)	RoboQoL CRF
		Missing (N%)	56 (35.2)	13 (21.7)	
	Phone Appointment	Mean (SD)	0.04 (0.2)	0.03 (0.18)	RoboQoL CRF
		Missing (N%)	57 (35.8)	13 (21.7)	
Nurse	Home Visit	Mean (SD)	0 (0)	0 (0)	RoboQoL CRF
		Missing (N%)	57 (35.8)	14 (23.7)	
	Surgery Visit	Mean (SD)	0.04 (0.24)	0.05 (0.22)	RoboQoL CRF
		Missing (N%)	57 (35.8)	14 (23.7)	
	Phone Appointment	Mean (SD)	0.03 (0.22)	0 (0)	RoboQoL CRF
		Missing (N%)	57 (35.8)	15 (25.9)	
Occupational Therapist (OT)	Home Visit	Mean (SD)	0 (0)	0 (0)	RoboQoL CRF
		Missing (N%)	56 (35.2)	14 (23.7)	
	Surgery Visit	Mean (SD)	0 (0)	0 (0)	RoboQoL CRF
		Missing (N%)	56 (35.2)	14 (23.7)	

	Hospital Visit	Mean (SD)	0 (0)	0 (0)	RoboQoL CRF
		Missing (N%)	56 (35.2)	14 (23.7)	
Physiotherapist	Home Visit	Mean (SD)	0 (0)	0 (0)	RoboQoL CRF
		Missing (N%)	56 (35.2)	14 (23.7)	
	Surgery Visit	Mean (SD)	56 (0)	14 (0)	RoboQoL CRF
		Missing (N%)	56 (35.2)	14 (23.7)	
	Phone Appointment	Mean (SD)	0 (0)	0 (0)	RoboQoL CRF
		Missing (N%)	56 (35.2)	14 (23.7)	
	Hospital Visit	Mean (SD)	0 (0)	0 (0)	RoboQoL CRF
		Missing (N%)	56 (35.2)	14 (23.7)	
	Day Visit	Mean (SD)	0 (0)	0 (0)	RoboQoL CRF
		Missing (N%)	56 (35.2)	15 (25.9)	
**Secondary Care**					
Outpatient Appointment		Mean (SD)	0.58 (0.58)	0.22 (0.22)	RoboQoL CRF
		Missing (N%)	58 (36.5)	14 (23.7)	
Ambulance See and treat		Mean (SD)	0.01 (0.1)	0.02 (0.13)	RoboQoL CRF
		Missing (N%)	56 (35.2)	14 (23.7)	
Ambulance see and convey		Mean (SD)	0.01 (0.1)	0.02 (0.13)	RoboQoL CRF
		Missing (N%)	56 (35.2)	14 (23.7)	
A&E		Mean (SD)	0.03 (0.22)	0.05 (0.39)	RoboQoL CRF
		Missing (N%)	57 (35.8)	14 (23.7)	
Hospital Ward Day		Mean (SD)	0 (0)	0.02 (0.13)	RoboQoL CRF
		Missing (N%)	57 (35.8)	14 (23.7)	
Hospital Overnight Visit		Mean (SD)	0 (0)	0.02 (0.13)	RoboQoL CRF
		Missing (N%)	57 (35.8)	14 (23.7)	
Week 12					
**Primary Care**					
General Practitioner (GP)	Home Visit	Mean (SD)	0.01 (0.1)	0 (0)	RoboQoL CRF
		Missing (N%)	51 (32.1)	15 (25.9)	
	Surgery Visit	Mean (SD)	0.06 (0.28)	0.05 (0.22)	RoboQoL CRF
		Missing (N%)	51 (32.1)	15 (25.9)	
	Phone Appointment	Mean (SD)	0.06 (0.31)	0.04 (0.19)	RoboQoL CRF
		Missing (N%)	51 (32.1)	16 (28.1)	
Nurse	Home Visit	Mean (SD)	0 (0)	0 (0)	RoboQoL CRF
		Missing (N%)	52 (32.7)	15 (25.9)	
	Surgery Visit	Mean (SD)	0.04 (0.23)	0.07 (0.26)	RoboQoL CRF
		Missing (N%)	52 (32.7)	15 (25.9)	
	Phone Appointment	Mean (SD)	0 (0)	0 (0)	RoboQoL CRF
		Missing (N%)	52 (32.7)	15 (25.9)	
Occupational Therapist (OT)	Home Visit	Mean (SD)	0 (0)	0 (0)	RoboQoL CRF
		Missing (N%)	53 (33.3)	15 (25.9)	
	Surgery Visit	Mean (SD)	0 (0)	0 (0)	RoboQoL CRF
		Missing (N%)	54 (34)	15 (25.9)	
	Hospital Visit	Mean (SD)	0 (0)	0 (0)	RoboQoL CRF
		Missing (N%)	54 (34)	15 (25.9)	

For peer review only

Physiotherapist	Home Visit	Mean (SD)	0 (0)	0.02 (0.13)	RoboQoL CRF
		Missing (N%)	53 (33.3)	15 (25.9)	
	Surgery Visit	Mean (SD)	53 (0)	15 (0)	RoboQoL CRF
		Missing (N%)	53 (33.3)	15 (25.9)	
	Phone Appointment	Mean (SD)	0 (0)	0 (0)	RoboQoL CRF
		Missing (N%)	53 (33.3)	15 (25.9)	
	Hospital Visit	Mean (SD)	0 (0)	0 (0)	RoboQoL CRF
		Missing (N%)	53 (33.3)	15 (25.9)	
	Day Visit	Mean (SD)	0 (0)	0 (0)	RoboQoL CRF
		Missing (N%)	53 (33.3)	15 (25.9)	
**Secondary Care**					
Outpatient Appointment		Mean (SD)	0.7 (0.7)	0.29 (0.29)	RoboQoL CRF
		Missing (N%)	54 (34)	15 (25.9)	
Ambulance See and treat		Mean (SD)	0 (0)	0 (0)	RoboQoL CRF
		Missing (N%)	53 (33.3)	15 (25.9)	
Ambulance see and convey		Mean (SD)	0 (0)	0 (0)	RoboQoL CRF
		Missing (N%)	53 (33.3)	15 (25.9)	
A&E		Mean (SD)	0 (0)	0 (0)	RoboQoL CRF
		Missing (N%)	53 (33.3)	15 (25.9)	
Hospital Ward Day		Mean (SD)	0.08 (0.62)	0 (0)	RoboQoL CRF
		Missing (N%)	53 (33.3)	15 (25.9)	
Hospital Overnight Visit		Mean (SD)	0 (0)	0 (0)	RoboQoL CRF
		Missing (N%)	53 (33.3)	15 (25.9)	

For peer review only

Supplemental Material S2: unit costs

Table S2.1 Unit costs of resources used during the surgical procedure including length of stay

Resource or unit intervention	Laparoscopic	Robotic	Cost per unit (£)	Cost Source
**Fixed costs of overheads**				
Operating theatre	Yes	Yes	250.00	South Tees Hospitals NHS Foundation based on personal communication with Alison Waines between Jan-Nov 2020
**Staff**				
**Resource or unit intervention**	**Laparoscopic**	**Robotic**	**Cost per unit (£)**	**Cost Source**
Consultant surgeon	Yes	Yes	109.00	PSSRU 2018/2019 based on "Consultant Surgical"
Assistant surgeon (registrar)	Yes	Yes	47.00	PSSRU 2018/2019 based on "Registrar"
Consultant anaesthetist	Yes	Yes	109.00	PSSRU 2018/2019 based on "Consultant medical"
Anaesthetist registrar	Yes	Yes	47.00	PSSRU 2018/2019 based on "Registrar"
Anaesthetist nurse	Yes	Yes	38.00	PSSRU 2018/2019 based on "hospital based nurses" (Band 5)
Nurse	Yes	Yes	38.00	PSSRU 2018/2019 based on "hospital based nurses" (Band 5)
Healthcare assistant	Yes	Yes	8.93	NHS Employers website 2018/2019 hourly rate (Band 2 with 3-4 years of experience)
Recovery practitioner	Yes	Yes	38.00	PSSRU 2018/2019 based on "hospital based nurses" (Band 5)
**Capital equipment**				
			**Cost (£)**	**Cost Source**

For peer review only

Resource or unit intervention	Laparoscopic	Robotic	Capital (life expectancy)	Annualised cost	Per case	South Tees Hospitals NHS Foundation based on personal communication with Alison Waines between Jan-Nov 2020
Laparoscopic device (screens included)	Yes	n/a	90 000.00 (8 years)	1 ,092.90	£17.25	KARL STORZ Endoscopy (UK) Ltd, based on personal communication with Matthew Taylor (Endoscopic sales specialist) in August 2020
Robotic device	n/a	Yes	1 000 300.00 (8 years)	145 520.29	191.73	South Tees Hospitals NHS Foundation based on personal communication with Jennifer Donnelly between Jan-Nov 2020
Robotic device maintenance cost	n/a	Yes	100 000.00 (per annum)	395.26	131.75	South Tees Hospitals NHS Foundation based on personal communication with Jeremy Twigg between Jan-Nov 2020
Diathermy console	Yes	Yes	10 800.75 (10 years)	1 298.70	1.71	South Tees Hospitals NHS Foundation based on personal communication with Jeremy Twigg between Jan-Nov 2020
Anaesthetic machine	Yes	Yes	20 000.00 (8 years)	2 909.53	3.83	South Tees Hospitals NHS Foundation based on personal communication with Jennifer Donnelly between Jan-Nov 2020
PCA pump	Yes	Yes	5 000.00 (5 years)	1 107.41	1.46	South Tees Hospitals NHS Foundation based on personal communication with Jennifer Donnelly between Jan-Nov 2020
Suction machine	Yes	Yes	2 000 (5 years)	442.96	0.58	South Tees Hospitals NHS Foundation based on personal communication with Jennifer Donnelly between Jan-Nov 2020
All annualised costs above have been annualised based on calculated annualisation factors, days worked per year is assumed as 253 days and equipment used on average three times per day.						
**Consumables Surgical equipment specific to laparoscopic and robotic surgery**						
**Resource or unit intervention**	**Laparoscopic**	**Robotic**	**Cost per unit (£)**			**Cost Source**
Gel port/trocar	Yes	n/a	26.00			South Tees Hospitals NHS Foundation based on personal communication with Alison Waines between Jan-Nov 2020
55mm dual port/trocar	Yes	n/a	96.00			South Tees Hospitals NHS Foundation based on personal communication with Alison Waines between Jan-Nov 2020

For peer review only

11mm port/trocar	Yes		24.00	South Tees Hospitals NHS Foundation based on personal communication with Alison Waines between Jan-Nov 2020
Verris lap pack	Yes	n/a	5.00	South Tees Hospitals NHS Foundation based on personal communication with Alison Waines between Jan-Nov 2020
Vcare manipulator	Yes	1	50.00	South Tees Hospitals NHS Foundation based on personal communication with Alison Waines between Jan-Nov 2020
Applied scissors	Yes	n/a	20.00	South Tees Hospitals NHS Foundation based on personal communication with Alison Waines between Jan-Nov 2020
Harmonic (ethicon)	Yes	n/a	550.00	South Tees Hospitals NHS Foundation based on personal communication with Alison Waines between Jan-Nov 2020
Misc. forceps	Yes	n/a	100.00	South Tees Hospitals NHS Foundation based on personal communication with Alison Waines between Jan-Nov 2020
Vlock suture	Yes	Yes	25.00	South Tees Hospitals NHS Foundation based on personal communication with Alison Waines between Jan-Nov 2020
Endoclose	Yes	Yes	10.00	South Tees Hospitals NHS Foundation based on personal communication with Alison Waines between Jan-Nov 2020
Syringes	Yes	Yes	0.20	South Tees Hospitals NHS Foundation based on personal communication with Alison Waines between Jan-Nov 2020
Scissors (robot specific)	n/a	Yes	342.00	South Tees Hospitals NHS Foundation based on personal communication with Alison Waines between Jan-Nov 2020
Pro-grasp	n/a	Yes	234.96	South Tees Hospitals NHS Foundation based on personal communication with Alison Waines between Jan-Nov 2020
Maryland bi-polar	n/a	Yes	289.20	South Tees Hospitals NHS Foundation based on personal communication with Alison Waines between Jan-Nov 2020
Needle holder	n/a	Yes	234.96	South Tees Hospitals NHS Foundation based on personal

For peer review only

				communication with Alison Waines between Jan-Nov 2020
Scope sterilisation for 30 degrees & 0 degrees	n/a	Yes	220.00	South Tees Hospitals NHS Foundation based on personal communication with Alison Waines between Jan-Nov 2020
Accessory set sterilisation	n/a	Yes	100.00	South Tees Hospitals NHS Foundation based on personal communication with Alison Waines between Jan-Nov 2020
**General consumable surgical equipment**				
**Resource or unit intervention**	**Laparoscopic**	**Robotic**	**Cost per unit (£)**	**Cost Source**
Arterial line	Yes	Yes	45.00	South Tees Hospitals NHS Foundation based on personal communication with Alison Waines and Richard Bickle between Jan-Nov 2020
Central line	Yes	Yes	140.00	South Tees Hospitals NHS Foundation based on personal communication with Alison Waines and Richard Bickle between Jan-Nov 2020
12F catheter	Yes	Yes	3.00	South Tees Hospitals NHS Foundation based on personal communication with Alison Waines and Richard Bickle between Jan-Nov 2020
Urine drainage bag	Yes	Yes	1.00	South Tees Hospitals NHS Foundation based on personal communication with Alison Waines and Richard Bickle between Jan-Nov 2020
Monocryl	Yes	Yes	48.00	South Tees Hospitals NHS Foundation based on personal communication with Alison Waines and Richard Bickle between Jan-Nov 2020
Bair Hugger warmer	Yes	Yes	6.00	South Tees Hospitals NHS Foundation based on personal communication with Alison Waines and Richard Bickle between Jan-Nov 2020
Fluid warmer	Yes	Yes	4.00	South Tees Hospitals NHS Foundation based on personal communication with Alison

For peer review only

				Waines and Richard Bickle between Jan-Nov 2020
Drape	1	n/a	22.30	South Tees Hospitals NHS Foundation based on personal communication with Alison Waines between Jan-Nov 2020
4 arm drape	n/a	Yes	276.00	South Tees Hospitals NHS Foundation based on personal communication with Alison Waines between Jan-Nov 2020
Scissor tip cover	n/a	1	18.15	South Tees Hospitals NHS Foundation based on personal communication with Alison Waines between Jan-Nov 2020
**Blood products**				
**Resource or unit intervention**	**Laparoscopic**	**Robotic**	**Cost per unit (£)**	**Cost Source**
Standard red blood cells	Yes	n/a	128.99	NHS Blood and Transplant price 2018/19
**Other resources**				
**Resource or unit intervention**	**Laparoscopic**	**Robotic**	**Cost per unit (£)**	**Cost Source**
Anaesthetic equipment	Yes	Yes	18.00	South Tees Hospitals NHS Foundation based on personal communication with Richard Bickle between Jan-Nov 2020
Anaesthetic drugs	Yes	Yes	47.00	South Tees Hospitals NHS Foundation based on personal communication with Richard Bickle between Jan-Nov 2020
IV fluids	Yes	Yes	5.50	South Tees Hospitals NHS Foundation based on personal communication with Richard Bickle between Jan-Nov 2020
**Type of stay**				
ICU	Yes	Yes	1 417.63	Fromhttps://www.nice.org.uk/guidance/GID-NG10072/documents/evidence-review-12inflated using PSSRU
Ward day	Yes	Yes	£416.90	From https://www.nice.org.uk/guidance/GID-NG10072/documents/evidence-

For peer review only

				review-12, inflated using PSSRU index

Table S2.2 Unit costs for NHS resources used during follow-up

Resource	Cost (£)	Source
GP Home	100.62	From Unit Costs of Health and Social Care 2019, a GP costs £4.30 per minute.<br>Unit Costs of Health and Social Care 2015, the average home visit is 11.4 minutes with 12 minutes of travel time.
GP Surgery	39	Costs from Unit Costs of Health and Social Care 2019
GP Phone	15.32	Costs from Unit Costs of Health and Social Care 2019
Nurse Home	16.38	Costs from Unit Costs of Health and Social Care 2019<br>We assumed the travel and contact time was the same as GP home visit.
Nurse Surgery	10.85	Costs from Unit Costs of Health and Social Care 2019<br>From Unit Costs of Health and Social Care 2015, the average surgery contact time was 15.5 minutes.
Nurse Phone	7.8	Cost from Unit Costs of Health and Social Care 2019
OT Home	46.4	Costs from Unit Costs of Health and Social Care 2019<br>From Unit Costs of Health and Social Care 2010 , an average OT session is 40 minutes.<br>We assumed the travel time was the same as a GP.
OT Surgery	32	Costs from Unit Costs of Health and Social Care 2019<br>From Unit Costs of Health and Social Care 2010 , an average OT session is 40 minutes.
OT Hospital	70	Costs from Unit Costs of Health and Social Care 2019
Physio Home	34.23	Costs from Unit Costs of Health and Social Care 2019, we assumed the average between bands 5 and 6.<br>We assumed same length appointment as OT (40 minutes).<br>We assumed the same travel time as GP.
Physio Surgery	26.33	Costs from Unit Costs of Health and Social Care 2019, we assumed the average between bands 5 and 6.<br>We assumed same length appointment as OT (40 minutes).
Physio Phone	4.56	Costs from Unit Costs of Health and Social Care 2019, we assumed the average between bands 5 and 6.<br>From Unit Costs of Health and Social Care 2019, we assumed same length phone call as a nurse.
Physio Hospital	58	Costs from Unit Costs of Health and Social Care 2019.
Physio Day Ward	58	Cost from Unit Costs of Health and Social Care 2019.
Outpatient	135	Cost from Unit Costs of Health and Social Care 2019.
Emergency Ambulance	196	Cost from Unit Costs of Health and Social Care 2019. We assumed the costs for "See and treat and refer"
Emergency Ambulance Hospital	258	Cost from Unit Costs of Health and Social Care 2019. We assumed the costs for "See and treat and convey"

For peer review only

Hospital A&E	135.8	Costs from NHS Reference costs 2018/2019, we assumed Accident and Emergency
Hospital Day Ward	416.9	Cost from https://www.nice.org.uk/guidance/GID-NG10072/documents/evidence-review-12. This was inflated to 2019 prices using Unit Costs of Health and Social Care 2019.
Hospital Overnight ward	416.9	Cost from https://www.nice.org.uk/guidance/GID-NG10072/documents/evidence-review-12. This was inflated to 2019 prices using Unit Costs of Health and Social Care 2019.
**Serious Adverse Events**		
Resource	Unit Cost (£)	Source
Sepsis	2 205	Weighted average of NHS tariff HRG codes: WJ06A, WJ06B, WJ06C, WJ06D, WJ06E, WJ06F, WJ06G, WJ06H, WJ06J
Bowel obstruction	3 340	Weighted average of NHS tariff HRG codes: FF61A, FF61B, FF61C
Embolism/CVA	3 334	Weighted average of NHS tariff HRG codes: AA35A, AA35B, AA35C, AA35D, AA35E, AA35F

For peer review only

Supplemental material S3: Patient flow diagram

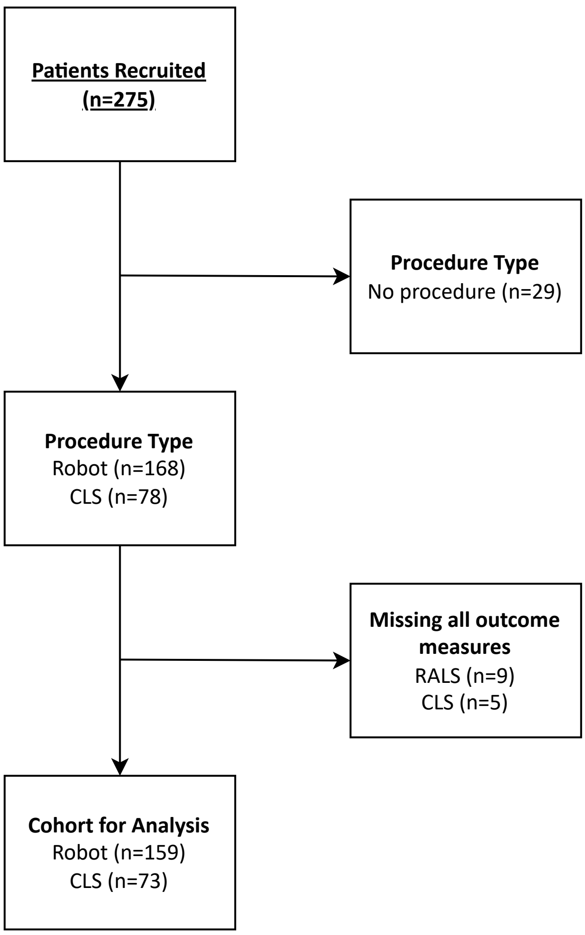

Supplementary material S4

S4.1: Patient characteristics and procedure information

Descriptor	RALS (n=159)	CLS (n=73)	P-value
Demographics (baseline)			
**Age (years)***			0.690
Mean ± SD	61.5 ± 11.8	60.8 ± 12.2	
Median (IQR)	62 (55 to 71)	61 (55 to 69)	
Unknown	4	0	
**BMI***			0.763
Mean ± SD	31.5 ± 8.0	30.6 ± 7.6	
Median (IQR)	30.9 (25.6 to 35.9)	29.2 (24.8 to 34.2)	
Unknown	11	4	
**Smoking Status**			0.849
Non-Smoker	116 (72.96%)	55 (75.34%)	
Ex-Smoker	6 (3.77%)	2 (2.74%)	
Smoker	26 (16.35%)	14 (19.18%)	
**Ethnicity**			0.011
White	129 (83.77%)	68 (93.15%)	
Non-White	25 (16.23%)	3 (4.11%)	
Unknown	11 (6.92%)	2 (2.74%)	
Medical history (baseline)			
**Number of prior surgeries**			0.476
0	129 (81.12%)	55 (75.34%)	
1	21 (15.72%)	15 (20.55%)	
2	8 (6.92%)	3 (4.11%)	
3	1 (0.63%)	0 (0%)	
Unknown	0 (0%)	0 (0%)	
**Histology**			0.145
Adenocarcinoma	15 (9.43%)	13 (17.81%)	
Aden squamous	2 (1.26%)	0 (0%)	
Benign	12 (7.55%)	10 (13.70%)	
Carcinoma sarcoma	5 (3.14%)	1 (1.37%)	
Clear cell	6 (3.77%)	1 (1.37%)	
Endometrioid	81 (50.94%)	36 (49.32%)	
Sarcoma	1 (0.63%)	1 (1.37%)	
Serous	24 (15.09%)	3 (4.11%)	
Squamous	6 (3.77%)	1 (1.37%)	
Unknown	7 (4.40%)	7 (9.59%)	
**Cancer Grade**			0.004

For peer review only

Benign	12 (7.55%)	10 (13.70%)	
1	41 (25.79%)	29 (39.73%)	
2	27 (16.98%)	11 (15.07%)	
3	46 (28.93%)	7 (9.59%)	
Unknown	33 (20.75%)	16 (21.92%)	
**Cancer Stage**			0.012
No Stage	12 (7.55)	10 (13.70%)	
1	110 (69.18%)	44 (60.27%)	
2	6 (3.77%)	8 (10.96%)	
3	15 (9.43%)	1 (1.37%)	
4	3 (1.89%)	0 (0%)	
Unknown	13 (8.18%)	10 (13.70%)	
Outcomes (baseline)			
**EORTC summary score**			0.754
Mean ± SD	86.29 ± 11. 38	86.79 ± 10.59	
Median (IQR)	89.10 (79.70 to 95.58)	89.87 (80.94 to 94.87)	
Unknown	11	5	
**EQ-5D-3L utility**			0.195
Mean ± SD	0.830 ± 0.150	0.800 ± 0.162	
Median (IQR)		0.837 (0.721 to 0.879)	
Unknown	11	5	
**6MWD (m)**			
Mean ± SD	374.80 ± 365.30	386.54 ± 109.71	0.454
Median (IQR)	365.3 (312.8 to 439.5)	400 (315 to 460)	
Unknown	11	6	
Surgical methods and length of stay			
**Lymph node dissection**			<0.001
Yes<br>*Full*<br>*Sentinel*	102 (64.14%)<br>*49*<br>*53*	6 (8.22%)<br>*3*<br>*3*	
No	57 (35.85%)	67 (91.78%)	
Unknown	0 (0%)	0 (0%)	
**Type of procedure**			0.166

For peer review only

Radical	16 (10.06%)	12 (16.44%)	
Simple	143 (89.94%)	61 (83.56%)	
Unknown	0 (0%)	0 (0%)	
**Type of hysterectomy**			0.263
With parametrium	18 (11.32%)	12 (16.44%)	
Without parametrium	141 (88.68%)	60 (82.19%)	
Unknown	0 (0%)	1 (1.37%)	
**Theatre times**			0.179
Mean ± SD	183 (68)	168 (93)	
Median IQR	172 (129 to 226)	151 (98 to 210)	
Unknown			
**Ward Days**			0.177
Mean SD	1.3 (2.1)	1.8 (2.4)	
Median IQR	1 (1,1)	1 (1,2)	
Unknown	0	0	
**ICU days**			0.774
Mean ± SD	0.08 (0.31)	0.07 (0.34)	
Median IQR	0 (0,0)	0 (0,0)	
Unknown	0	0	
**Total Length of Stay**			0.199
Mean ± SD	1.4 (2.1)	1.9 (2.4)	
Median IQR	1 (1,1)	1 (1,2)	
Unknown	0	0	

Data presented as n(%) unless otherwise stated
CLS conventional laparoscopic surgery
RALS robotically assisted laparoscopic surgery

For peer review only

Supplemental Material S5

Table S5.1 list of serious adverse events

Serious Adverse event	Event time	RALS (n=159)	CLS (n=73)
Abdominal infection	Post-procedure	0	1
Accidental dislodgement of a needle	Post-procedure	0	1
CVA and DVT	Post-procedure	1	0
Haematoma with active bleeding	Post-procedure	1	0
lower suprapubic abdominal pain	Post-procedure	1	0
Nausea and vomiting	Post-procedure	2	0
Nervous system disorders	Post-procedure	1	0
Vaginal prolapse	Post-procedure	0	1
Sepsis	Week-1	0	2
Bowel obstruction	Week-2	0	1
CVA	Week-2	1	0

For peer review only

Supplemental material S6

S6.1: Procedure and post-procedure to discharge cost summaries for CLS and RALS patients.

Cost component	RALS (n=159)	CLS (n=73)	P
**Procedure Costs**			
**Staff cost**			**0.231**
Mean ± SD	1 851 (688)	1 703 (940)	
Median (IQR)	1 733 (1 068 to 2 278)	1 522 (987 to 2 117)	
**Theatre cost**			**0.231**
Mean ± SD	765 (284)	704 (388)	
Median (IQR)	716 (537 to 941)	630 (408 to 875)	
**Blood Products Cost**			**0.848**
Mean ± SD	7 (42)	9 (62)	
Median (IQR)	0 (0 to 0)	0 (0 to 0)	
**Equipment cost***	2 134	1 325	
**Total Procedure Cost**			**<0.001**
Mean ± SD	4 758 (972)	3 741 (1 350)	
Median (IQR)	4 585 (3 972 to 5 356)	3 477 (2 722 to 4 317)	
**Length of stay cost**			**0.352**
Mean ± SD	704 (1 002)	851 (1 159)	
Median (IQR)	416 (416 to 416)	416 (416 to 833)	**0.002**
**Total cost**			
Mean ± SD	5 462 (1 472)	4 592 (2 072)	
Median (IQR)	5 126 (4 404 to 6 142)	3 905 (3 195 to 5 162)	

All data are presented as mean ± (SD) or median (interquartile range (IQR)) unless otherwise stated. All numbers of costs in £ Sterling.
*Equipment costs are from what are used in a 'typical' procedure and are assumed a fixed cost across all patients in the same cohort.
SD standard deviation, IQR interquartile range, CLS – conventional laparoscopic surgery, RALS robotically assisted laparoscopic surgery.

S6.2 Summary information for follow-up costs of RALS and CLS patients

Cost component	RALS (n=159)	CLS (n=73)	P
**Week 1**			**0.092**
Mean ± SD	125 ± 333	336 ± 996	
Median (IQR)	0 (0 to 115)	0 (0 to 143)	
Missing	45	9	
**Week 2**			**0.188**
Mean ± SD	94 ± 300	246 ± 907	
Median (IQR)	0 (0 to 135)	0 (0 to 15)	
Missing	45	7	

For peer review only

**Week 3**			**0.578**
Mean ± SD	69 ± 135	103 ± 496	
Median (IQR)	0 (0 to 135)	0 (0 to 39)	
Missing	47	11	
**Week 4**			**0.084**
Mean ± SD	55 ± 87	30 ± 81	
Median (IQR)	0 (0 to 135)	0 (0 to 11)	
Missing	52	10	
**Week 5**			**0.748**
Mean ± SD	40 ± 71	33 ± 99	
Median (IQR)	0 (0 to 39)	0 (0 to 11)	
Missing	61	15	
**Week 6**			**0.268**
Mean ± SD	49 ± 95	29 ± 99	
Median (IQR)	0 (0 to 54)	0 (0 to 135)	
Missing	56	16	
**Week 7**			**0.187**
Mean ± SD	80 ± 145	44 ± 153	
Median (IQR)	0 (0 to 135)	0 (0 to 11)	
Missing	61	15	
**Week 8**			**0.023**
Mean ± SD	88 ± 186	31 ± 101	
Median (IQR)	0 (0 to 50)	0 (0 to 11)	
Missing	62	16	
**Week 9**			**0.784**
Mean ± SD	77 ± 183	91 ± 380	
Median (IQR)	0 (0 to 15)	0 (0 to 39)	
Missing	58	14	
**Week 10**			**0.891**
Mean ± SD	96 ± 209	87 ± 329	
Median (IQR)	0 (0 to 39)	0 (0 to 11)	
Missing	58	14	
**Week 11**			**0.608**
Mean ± SD	84 ± 214	64 ± 164	
Median (IQR)	0 (0 to 15)	0 (0 to 15)	
Missing	61	15	
**Week 12**			**0.049**
Mean ± SD	116 ± 331	44 ± 110	
Median (IQR)	0 (0 to 39)	0 (0 to 26)	
Missing	55	16	

For peer review only

For peer review only

Supplemental Material S7

Fig. S7.1: Graph A: Cost-effectiveness plane for adjusted sensitivity analysis assuming 1 procedure per day. Graph B: Willingness to pay for adjusted sensitivity analysis assuming 1 procedure per day

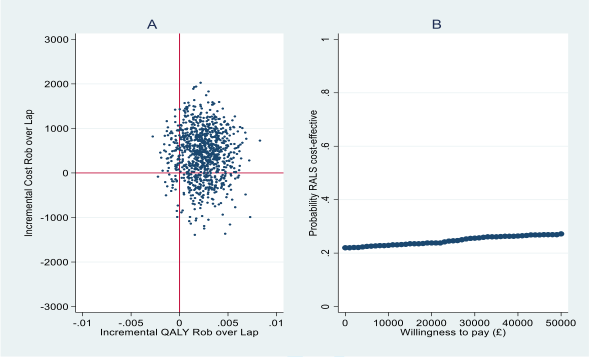

Fig. S7.2: Graph A: Cost-effectiveness plane for adjusted sensitivity analysis assuming 2 procedures per day. Graph B: Willingness to pay for adjusted sensitivity analysis assuming 2 procedures per day

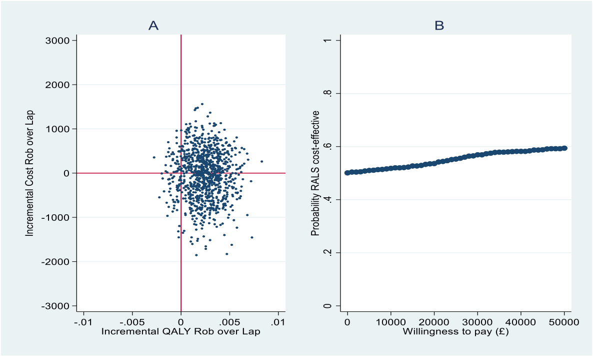

For peer review only

Fig. S7.3: Graph A: Cost-effectiveness plane for adjusted sensitivity analysis assuming 4 procedures per day. Graph B: Willingness to pay for adjusted sensitivity analysis assuming 4 procedures per day

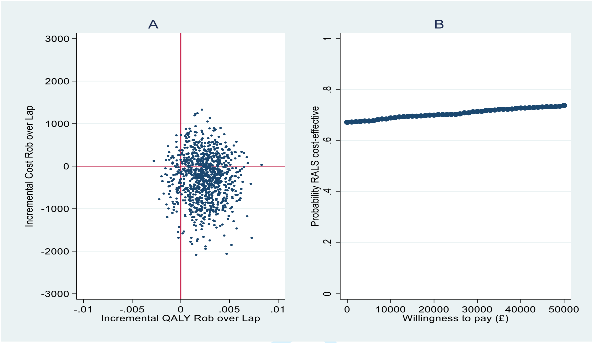

For peer review only

STROBE Statement—Checklist of items that should be included in reports of ***cohort studies***

	Item No	Recommendation	Page No
**Title and abstract**	1	(*a*) Indicate the study's design with a commonly used term in the title or the abstract	1
		(*b*) Provide in the abstract an informative and balanced summary of what was done and what was found	2
**Introduction**			
Background/rationale	2	Explain the scientific background and rationale for the investigation being reported	3,4
Objectives	3	State specific objectives, including any prespecified hypotheses	4
**Methods**			
Study design	4	Present key elements of study design early in the paper	4
Setting	5	Describe the setting, locations, and relevant dates, including periods of recruitment, exposure, follow-up, and data collection	4
Participants	6	(*a*) Give the eligibility criteria, and the sources and methods of selection of participants. Describe methods of follow-up	4,5
		(*b*) For matched studies, give matching criteria and number of exposed and unexposed	
Variables	7	Clearly define all outcomes, exposures, predictors, potential confounders, and effect modifiers. Give diagnostic criteria, if applicable	5,6
Data sources/ measurement	8*	For each variable of interest, give sources of data and details of methods of assessment (measurement). Describe comparability of assessment methods if there is more than one group	5,6
Bias	9	Describe any efforts to address potential sources of bias	5,6
Study size	10	Explain how the study size was arrived at	N/A
Quantitative variables	11	Explain how quantitative variables were handled in the analyses. If applicable, describe which groupings were chosen and why	5,6
Statistical methods	12	(*a*) Describe all statistical methods, including those used to control for confounding	5,6
		(*b*) Describe any methods used to examine subgroups and interactions	
		(*c*) Explain how missing data were addressed	
		(*d*) If applicable, explain how loss to follow-up was addressed	
		(*e*) Describe any sensitivity analyses	
**Results**			
Participants	13*	(a) Report numbers of individuals at each stage of study—eg numbers potentially eligible, examined for eligibility, confirmed eligible, included in the study, completing follow-up, and analysed	7
		(b) Give reasons for non-participation at each stage	
		(c) Consider use of a flow diagram	
Descriptive data	14*	(a) Give characteristics of study participants (eg demographic, clinical, social) and information on exposures and potential confounders	7
		(b) Indicate number of participants with missing data for each variable of interest	
		(c) Summarise follow-up time (eg, average and total amount)	
Outcome data	15*	Report numbers of outcome events or summary measures over time	7,8

For peer review only - http://bmjopen.bmj.com/site/about/guidelines.xhtml
For peer review only

Main results	16	(*a*) Give unadjusted estimates and, if applicable, confounder-adjusted estimates and their precision (eg, 95% confidence interval). Make clear which confounders were adjusted for and why they were included<br>(*b*) Report category boundaries when continuous variables were categorized<br>(*c*) If relevant, consider translating estimates of relative risk into absolute risk for a meaningful time period	7,8
Other analyses	17	Report other analyses done—eg analyses of subgroups and interactions, and sensitivity analyses	8
**Discussion**			
Key results	18	Summarise key results with reference to study objectives	9,10,11
Limitations	19	Discuss limitations of the study, taking into account sources of potential bias or imprecision. Discuss both direction and magnitude of any potential bias	11
Interpretation	20	Give a cautious overall interpretation of results considering objectives, limitations, multiplicity of analyses, results from similar studies, and other relevant evidence	11,12
Generalisability	21	Discuss the generalisability (external validity) of the study results	9,10,11
**Other information**			
Funding	22	Give the source of funding and the role of the funders for the present study and, if applicable, for the original study on which the present article is based	21

*Give information separately for exposed and unexposed groups.

Note: An Explanation and Elaboration article discusses each checklist item and gives methodological background and published examples of transparent reporting. The STROBE checklist is best used in conjunction with this article (freely available on the Web sites of PLoS Medicine at http://www.plosmedicine.org/, Annals of Internal Medicine at http://www.annals.org/, and Epidemiology at http://www.epidem.com/). Information on the STROBE Initiative is available at http://www.strobe-statement.org.

For peer review only

For peer review only - http://bmjopen.bmj.com/site/about/guidelines.xhtml

CHEERS 2022 Checklist

Topic	No.	Item	Location where item is reported
**Title**			
	1	Identify the study as an economic evaluation and specify the interventions being compared.	Title, page 1
**Abstract**			
	2	Provide a structured summary that highlights context, key methods, results, and alternative analyses.	Abstract, page 2
**Introduction**			
**Background and objectives**	3	Give the context for the study, the study question, and its practical relevance for decision making in policy or practice.	Pages 3,4
**Methods**			
**Health economic analysis plan**	4	Indicate whether a health economic analysis plan was developed and where available.	Not Reported
**Study population**	5	Describe characteristics of the study population (such as age range, demographics, socioeconomic, or clinical characteristics).	Pages 4,5
**Setting and location**	6	Provide relevant contextual information that may influence findings.	Page 4,5
**Comparators**	7	Describe the interventions or strategies being compared and why chosen.	Page 5
**Perspective**	8	State the perspective(s) adopted by the study and why chosen.	Page 6
**Time horizon**	9	State the time horizon for the study and why appropriate.	Page 6
**Discount rate**	10	Report the discount rate(s) and reason chosen.	N/A
**Selection of outcomes**	11	Describe what outcomes were used as the measure(s) of benefit(s) and harm(s).	Page 6
**Measurement of outcomes**	12	Describe how outcomes used to capture benefit(s) and harm(s) were measured.	Pages 5,6

For peer review only - http://bmjopen.bmj.com/site/about/guidelines.xhtml

Topic	No.	Item	Location where item is reported
**Valuation of outcomes**	13	Describe the population and methods used to measure and value outcomes.	Pages 6
**Measurement and valuation of resources and costs**	14	Describe how costs were valued.	Pages 6
**Currency, price date, and conversion**	15	Report the dates of the estimated resource quantities and unit costs, plus the currency and year of conversion.	Page 6
**Rationale and description of model**	16	If modelling is used, describe in detail and why used. Report if the model is publicly available and where it can be accessed.	N/A
**Analytics and assumptions**	17	Describe any methods for analysing or statistically transforming data, any extrapolation methods, and approaches for validating any model used.	N/A
**Characterising heterogeneity**	18	Describe any methods used for estimating how the results of the study vary for subgroups.	N/A
**Characterising distributional effects**	19	Describe how impacts are distributed across different individuals or adjustments made to reflect priority populations.	N/A
**Characterising uncertainty**	20	Describe methods to characterise any sources of uncertainty in the analysis.	page 6
**Approach to engagement with patients and others affected by the study**	21	Describe any approaches to engage patients or service recipients, the general public, communities, or stakeholders (such as clinicians or payers) in the design of the study.	N/A
**Results**			
**Study parameters**	22	Report all analytic inputs (such as values, ranges, references) including uncertainty or distributional assumptions.	Pages 7,8
**Summary of main results**	23	Report the mean values for the main categories of costs and outcomes of interest and summarise them in the most appropriate overall measure.	Page 8
**Effect of uncertainty**	24	Describe how uncertainty about analytic judgments, inputs, or projections affect findings. Report the effect of choice of discount rate and time horizon, if applicable.	Pages 8,9

For peer review only - http://bmjopen.bmj.com/site/about/guidelines.xhtml

Topic	No.	Item	Location where item is reported
**Effect of engagement with patients and others affected by the study**	25	Report on any difference patient/service recipient, general public, community, or stakeholder involvement made to the approach or findings of the study	N/A
**Discussion**			
**Study findings, limitations, generalisability, and current knowledge**	26	Report key findings, limitations, ethical or equity considerations not captured, and how these could affect patients, policy, or practice.	Pages 9,10,11
**Other relevant information**			
**Source of funding**	27	Describe how the study was funded and any role of the funder in the identification, design, conduct, and reporting of the analysis	Page 13
**Conflicts of interest**	28	Report authors conflicts of interest according to journal or International Committee of Medical Journal Editors requirements.	Page 12

From: Husereau D, Drummond M, Augustovski F, et al. Consolidated Health Economic Evaluation Reporting Standards 2022 (CHEERS 2022) Explanation and Elaboration: A Report of the ISPOR CHEERS II Good Practices Task Force. Value Health 2022;25. doi:10.1016/j.jval.2021.10.008

For peer review only

BMJ Open

Robotic and Laparoscopic Gynaecological Surgery: A Prospective Multi-Centre Observational Cohort Study and Economic Evaluation in England.

Journal:	*BMJ Open*
Manuscript ID	bmjopen-2023-073990.R1
Article Type:	Original research
Date Submitted by the Author:	17-Aug-2023

Complete List of Authors:	McCarthy, Andrew; Northumbria University, Department of Nursing, Midwifery and Health<br>Samarakoon, Dilupa; Northumbria University, Department of Nursing, Midwifery and Health<br>Gray, Joanne; Northumbria University,<br>Mcmeekin, Peter; Northumbria University, Faculty of Health and Life Sciences<br>McCarthy, Stephen; Northumbria University, Department of Nursing, Midwifery and Health<br>Newton, Claire; University Hospitals Bristol and Weston NHS Foundation Trust, Department of Gynaecology Oncology; University of Bristol<br>Nobbenhuis, Marielle; Royal Marsden NHS Foundation Trust, Department of Gynaecological Oncology<br>Lippiatt, Jonathan; University Hospitals Dorset NHS Foundation Trust, Department of Gynaecological Oncology<br>Twigg, Jeremy; Coventry and Warwickshire Hospital, Department of Gynaecological Oncology

**Primary Subject Heading**:	Surgery
Secondary Subject Heading:	Health economics
Keywords:	Gynaecological oncology < GYNAECOLOGY, Minimally invasive surgery < GYNAECOLOGY, HEALTH ECONOMICS

SCHOLARONE™
Manuscripts

I, the Submitting Author has the right to grant and does grant on behalf of all authors of the Work (as defined in the below author licence), an exclusive licence and/or a non-exclusive licence for contributions from authors who are: i) UK Crown employees; ii) where BMJ has agreed a CC-BY licence shall apply, and/or iii) in accordance with the terms applicable for US Federal Government officers or employees acting as part of their official duties; on a worldwide, perpetual, irrevocable, royalty-free basis to BMJ Publishing Group Ltd ("BMJ") its licensees and where the relevant Journal is co-owned by BMJ to the co-owners of the Journal, to publish the Work in this journal and any other BMJ products and to exploit all rights, as set out in our licence.

The Submitting Author accepts and understands that any supply made under these terms is made by BMJ to the Submitting Author unless you are acting as an employee on behalf of your employer or a postgraduate student of an affiliated institution which is paying any applicable article publishing charge ("APC") for Open Access articles. Where the Submitting Author wishes to make the Work available on an Open Access basis (and intends to pay the relevant APC), the terms of reuse of such Open Access shall be governed by a Creative Commons licence – details of these licences and which Creative Commons licence will apply to this Work are set out in our licence referred to above.

Other than as permitted in any relevant BMJ Author's Self Archiving Policies, I confirm this Work has not been accepted for publication elsewhere, is not being considered for publication elsewhere and does not duplicate material already published. I confirm all authors consent to publication of this Work and authorise the granting of this licence.

For peer review only

Robotic and Laparoscopic Gynaecological Surgery: A Prospective Multi-Centre Observational Cohort Study and Economic Evaluation in England.

Authors:

Andrew McCarthy[1*], Dilupa Samarakoon[1], Joanne Gray[1], Peter McMeekin[1], Stephen McCarthy[1], Claire Newton[2,3], Marielle Nobbenhuis[4], Jonathan Lippiatt[5], Jeremy Twigg[6]

Institutions:

1 Department of Nursing, Midwifery and Health, Northumbria University, Newcastle Upon Tyne, UK

2 Department of Gynaecology Oncology, St Michaels hospital, University Hospitals Bristol and Weston NHS foundation trust, UK.

3. University of Bristol, Beaconhouse, Queens Road, Bristol, BS8 1QU, UK

4 Department of Gynaecological Oncology, The Royal Marsden NHS Foundation Trust, Chelsea, London SW3 6JJ, UK

5 Department of Gynaecological Oncology, University Hospitals Dorset NHS Foundation Trust, Poole, UK

6 Department of Gynaecological Oncology, University Hospital of Coventry and Warwickshire, Coventry, UK

**Correspondence to:* Andrew McCarthy, Coach Lane Campus, Northumbria University, Newcastle upon Tyne, NE7 7XA, United Kingdom. Telephone: 0191 215 6718. Email: andrew2.mccarthy@northumbria.ac.uk

For peer review only

ABSTRACT

Objective

To compare the health related quality of life and cost effectiveness of robot-assisted laparoscopic surgery (RALS) versus conventional "*straight stick*" laparoscopic surgery (CLS) in women undergoing hysterectomy as part of their treatment for either suspected or proven gynaecological malignancy.

Design

Multicentre prospective observational cohort study.

Setting

Patients aged 16+ undergoing hysterectomy as part of their treatment for gynaecological malignancy at 12 NHS cancer units and centres in England between August 2017 and February 2020.

Participants

275 patients recruited with 159 RALS, 73 CLS eligible for analysis.

Outcome measures

Primary outcome was the European Organisation for Research and Treatment of Cancer Quality of Life measure (EORTC). Secondary outcomes included EQ-5D-5L utility, six-minute walk test (6MWT), NHS costs using pounds Sterling (£) 2018-19 prices and cost-effectiveness. The cost-effectiveness evaluation compared EQ-5D-5L quality adjusted life years and costs between RALS and CLS.

Results

No difference identified between RALS and CLS for EORTC, EQ-5D-5L utility, and 6MWT. RALS had unadjusted mean cost difference of £556 (95%CI -£314 to £1 315) versus CLS and mean quality adjusted life year (QALY) difference of 0.0024 (95%CI -0.00051 to 0.0057), non-parametric ICER of £152 843 per QALY. For the adjusted cost-effectiveness analysis, RALS dominated CLS with a mean

For peer review only

cost difference of £-188 (95%CI -£1 321 to £827) and QALY difference of 0.0024 (95%CI -0.0008 to 0.0057).

Conclusions

Findings suggest that RALS versus CLS in women undergoing hysterectomy (after adjusting for differences in morbidity) is cost effective with lower costs and QALYs. Results are highly sensitive to the usage of robotic hardware with higher usage increasing the probability of cost-effectiveness. Non-inferiority RCT would be of benefit to decision makers to provide further evidence on the cost-effectiveness of RALS versus CLS but may not be practical due to surgical preferences of surgeons and the extensive roll out of RALS.

For peer review only

Strengths and limitations of this study

- Regression methods were used to account for differences in baseline characteristics between groups.
- Cost estimates were derived from detailed micro-costing of resource use.
- Economic evaluation adhered to CHEERS guidelines and used appropriate recommended methods
- Follow-up limited to 12 weeks post-surgery and as such does not consider potential longer-term effects.

For peer review only - http://bmjopen.bmj.com/site/about/guidelines.xhtml

INTRODUCTION

Background

Surgery used to treat early-stage gynaecological cancer has changed over the last 20 years through the introduction of minimal access surgical approaches. Surgeons have developed “keyhole” (laparoscopic) techniques using “straight stick” surgical instruments. This technique is termed conventional laparoscopic surgery (CLS). A number of studies have randomized patients with gynaecological cancer to CLS or open surgery. The GOG LAP2 study showed that patients receiving CLS had an improved safety profile, less antibiotic use and shorter hospital stay as well as higher quality of life scores (better physical functioning, better body image, less pain), earlier resumption of normal activities and earlier return to work over the 6 weeks following surgery(1), compared to laparotomy in patients with endometrial cancer. A second trial (LACE study) showed quality of life improvements and an adverse event profile that favoured CLS compared to OH (Open Hysterectomy)(2). A Dutch study also confirmed that a laparoscopic approach provided benefit with respect to shorter hospital stay, less pain, and quicker resumption of daily activities compared to laparotomy(1). CLS in gynaecological oncology has not been a panacea. The LAP2 and Dutch studies showed that despite the use of CLS, conversion rates varied from 10% - 26%, and this rate increased to 57% in women with a body mass index (BMI) greater than 40 kg/m2. Further, uptake of CLS in the UK has been slow and fragmented: for example, the ASTEC study (A Study in the Treatment of Endometrial Cancer, which recruited from 1998 to 2005) demonstrated that only 7% of patients being treated for endometrial cancer had a laparoscopic hysterectomy, whilst the remaining 93% had open surgery(3). Whilst the adverse events were lower in the CLS group compared to the OH group in ASTEC, a third of CLS patients in this study had their procedure converted to open surgery.

The introduction of robot assisted laparoscopic surgery (RALS) has facilitated a greater number of surgeons being able to offer minimal access surgery to their patients(4). The combination of a wristed instrument design and 3-D high definition optics of the robot, allows the surgeon to dissect

For peer review only - http://bmjopen.bmj.com/site/about/guidelines.xhtml

remove and reconstruct tissues in a way that has not been possible (5). Outcomes for RALS appear favourable to open surgery and there are a considerable number of publications suggesting that standard hospital performance indicators are as good for RALS as they are for other surgical techniques(6-9). However, RALS brings with it a greater cost than CLS: operative times are longer, and equipment costs are greater for benign as well as oncological gynaecological procedures with no difference in key performance indicators(4, 10). A recent Cochrane review concluded "…we found limited evidence on the effectiveness and safety of RALS compared with CLS or open surgery for surgical procedures performed for gynaecological cancer; therefore, its use should be limited to clinical trials. Ongoing trials are likely to have an important impact on evidence related to the use of RALS in gynaecology…"(11).

As a consequence, health care commissioners and policy makers in the UK have made RALS an area of investigation so that informed decisions can be made about the routine commissioning of robotics. For now, NHS England have routinely commissioned RALS for the treatment of prostate cancer but for all other RALS services, robotic consumables are excluded from NHS tariff(12, 13), which is a process in which NHS providers in England receive healthcare funding . Simultaneous to the debate around healthcare costs, health and social care policymakers have focused on patient-reported outcome measures (PROMs). These are increasingly recognised as important in healthcare resource allocation decisions both globally and in the NHS(14). NHS England analysis of PROMs collected following surgical procedures enables "…providers, commissioners and other stakeholders to make informed changes to the delivery of their services…"(15). This data feeds into key NHS Outcomes Framework Indicators and the evidence of performance in these domains impacts on funding decisions across the healthcare system(16) .

There is little research which compares health-related quality of life between RALS and CLS. Whilst Herling et al provided evidence of a speedy recovery in many domains following RALS, comparison

For peer review only - http://bmjopen.bmj.com/site/about/guidelines.xhtml

of health related quality of life recovery across different modes of surgery has not been explored(17). Kumar et al attempted a comparison between open surgery and RALS, looking retrospectively at comparative health related quality of life in endometrial cancer(18). They found that RALS was associated with improved health related quality of life compared with an open approach. The study however, did not compare RALS with CLS, and nor did it use validated health related quality of life questionnaires or use recommended metrics for endometrial cancer(19).

Any future decisions on provision of RALS in gynaecological cancer should be determined by informed decision making of both the effectiveness and cost-effectiveness of this intervention compared to CLS. However, there is a paucity of data regarding comparative cost- effectiveness in this area.

Objectives

The aims of this study were to compare the effectiveness in terms of health related quality of life, and cost effectiveness of robot-assisted laparoscopic surgery (RALS) versus conventional "*straight stick*" laparoscopic surgery (CLS) in women undergoing hysterectomy as part of their treatment for either suspected or proven gynaecological malignancy.

METHODS

Study design

This prospective comparative observational cohort study with embedded cost-effectiveness analysis recruited patients between August 2017 and February 2020 across 12 NHS Cancer Units and Centres in England. Patients referred for suspected gynaecological cancer or with a proven diagnosis of endometrial, cervical, and ovarian cancer were given a patient information sheet and asked for consent to be part of this study. Demographic and anthropometric data was collected at baseline

For peer review only - http://bmjopen.bmj.com/site/about/guidelines.xhtml

alongside relevant outcome measures. and participants were followed up at 2-, 4- and 12-weeks post-procedure.

Participant inclusion criteria

Inclusion criteria consisted of patients aged over 16 years who were referred with diagnosis (or suspected diagnosis) of gynaecological malignancy and about to undergo surgery RALS or CLS. Patients were also required to be fit for surgery, have the ability to complete the six-minute walk test (6MWT) and they were also required to be having a hysterectomy as part of their surgical treatment to be eligible.

Sites were responsible for ensuring that patients had the capacity to give informed consent. A consent form which included an approved patient information sheet was provided in English to potential participants, with an offer for an interpreter where necessary to ensure full informed consent from participants. In addition, an appropriately trained staff member at each site would provide a full explanation of the trial and additional trial requirements prior to trial entry of participants. An adequate amount of time was given to patients to consider the information and to discuss participation in the trial. Written informed consent was obtained before any trial specific procedures were conducted. Furthermore, parental consent was also required to be obtained for patients aged between 16 and 18.

Patients were excluded if surgery was only possible via the abdominal approach or if the midline laparotomy incision was anticipated to go beyond 4cm above the umbilicus. Other exclusion criteria were surgery for a large ovarian tumour and patients undergoing radical cytoreductive surgery. Patients were also excluded if they were unable to complete outcome measures due to cognitive or language impairments disabling them from completing the quality-of-life questionnaires or lacked capacity to give consent.

Public and patient involvement

For peer review only - http://bmjopen.bmj.com/site/about/guidelines.xhtml

Patient and Public Involvement were not included in this research project.

Surgical Procedures

Surgical procedures followed established practice for performing hysterectomy and bilateral salpingo-oophorectomy with/without pelvic lymph node dissection according to local cancer network guidelines for FIGO Stage of disease (International Federation of Gynaecology and Obstetrics) as determined by availability of either surgically skilled robotic or laparoscopic surgeons.

Primary Outcome

The primary outcome for this study was the European Organisation for Research and Treatment of Cancer Quality of Life (EORTC QLQ C-30) health related quality of life measure. The EORTC QLQ-C30 is a 30-item questionnaire assessing quality of life in cancer patients. It contains five functional scales (physical, role, cognitive, emotional, and social), three symptom scales (fatigue, pain, and nausea and vomiting), and a global health and quality-of-life scale. The remaining single items assess additional symptoms commonly reported by cancer patients (dyspnoea, appetite loss, sleep disturbance, constipation, and diarrhoea), as well as the perceived financial impact of the disease and treatment. Higher scores indicate better function and heightened symptoms. The QLQ C30 summary score is a validated measure for assessing quality of life for cancer patients and it presented as recommended by the EORTC Quality of Life Group(20). The summary score is based on 27 items, the quality-of-life scale and the financial impact item are excluded(20).

Secondary Outcomes

Secondary outcome measures included functional capacity at follow-up measured using the six-minute walk test (6MWT), health related quality of life (EQ-5D-5L QALYs), NHS costs and cost-effectiveness. The 6MWT is a sub-maximal test of aerobic capacity which requires the individual to walk as far as possible during six minutes around a 30-meter course and the distance covered is

For peer review only - http://bmjopen.bmj.com/site/about/guidelines.xhtml

recorded. It is a validated measure which has been recommended for use in cancer patients(21). Furthermore, the 6MWThas been applied in a clinical setting in a number of conditions as well as pre- and post-operatively(22, 23). The EQ-5D-5L comprises five domains each assessing a specific dimension of health related quality of life (mobility, self-care, usual activities, pain, and anxiety and depression) with three response levels (‘‘no problems’’, ‘‘some or moderate problems’’, and ‘‘extreme problems’’)(24, 25). Responses are converted into a weighted single utility score on a scale from 1 (perfect health) to 0 (death) in order to estimate Quality Adjusted Life Years (QALYs)(24, 25).

Statistical methods

Reporting of study findings was informed by the Strengthening the Reporting of Observational studies (STROBE) guidelines(26). Sample size was calculated for the primary outcome of this study, the health-related quality of life measured by the EORTC QLQ C-30. Minimum clinically important difference of the global health score of the EORTC QLQ C-30 was defined as a standardised mean difference of 0.3 SDs(27). With 90% power (beta) and a 90% confidence interval (alpha) it was anticipated that the study would require a total of 138 patients in each arm (with a maximum unequal allocation ratio of 2:1). Allowing for 25% attrition, this study aimed to recruit 173 patients in each cohort.

Patient characteristics and differences in surgical methods were compared using the appropriate statistical tests (t-test for continuous variables and χ^2 test for categorical variables). The EORTC QLQ C-30 summary score, 6MWT, and EQ-5D-5L utility were evaluated using a constrained baseline longitudinal regression model(28). The equality at baseline constraint was imposed to counteract regression to the mean, whereby, a group of patients with a worse average baseline score generally improve more than the group with better baseline scores, independent of any intervention effect(29). This type of regression model also accounts for the hierarchical data structure and provides a principled method for dealing with missing outcome data, including missing baseline data with baseline as part of the outcome vector in this linear mixed model(28). A Satterwhite correction

For peer review only - http://bmjopen.bmj.com/site/about/guidelines.xhtml

For peer review only

of degrees of freedom was applied. The model adjusts for potential confounders identified as differences in baseline characteristics and surgical methods as fixed effects, alongside each individual patient modelled as a random effect. Parameters for interaction terms between time and treatment represent the estimated treatment effects at each follow-up. Treatment effects are reported with 95% confidence intervals (95% C.I.) and p-values. P-values of less than 0.05 were considered statistically significant. All analyses were undertaken using Stata version 15.1.

Economic Evaluation

Estimating the cost of surgery

The costing perspective was that of the U.K. National Health Service (NHS) with all prices being in pounds sterling (£) using 2018-19 prices. In order to increase precision and transferability into international contexts, the cost of each surgical procedure was micro-costed with resource use including equipment, staffing, and consumables being collected from the study specific case report forms (CRF) and expert clinical guidance. General ward and Intensive Care Unit (ICU) length of stay (LOS) following surgery until discharge were also collected from the study CRF. Following discharge, resource use was collected weekly for 12 weeks post-surgery using a Health Service Utilisation Questionnaire CRF. Follow-up resource use included primary care services e.g., general practice visits, district nurse visits as well as secondary care services e.g., inpatient stays, and outpatient visits. Serious adverse events from initial admission through to final follow-up were also recorded using a study specific CRF. Resource use is reported in supplemental material S1.

Unit costs were derived from routine data sources(30, 31) alongside study specific estimates. All re-usable equipment were assigned a ‘per use’ cost. Where equipment had a short re-usable lifespan (e.g., 10 uses), the unit cost was divided by the number of uses to get a cost per use. Capital equipment such as laparoscopic stack systems and robotic components (as opposed to single or limited use surgical instrumentation) with prolonged lifespans were annuitized using a discount rate

For peer review only - http://bmjopen.bmj.com/site/about/guidelines.xhtml

of 3.5%(32) to estimate yearly costs. These were then converted to a cost per use by assuming 3 surgical procedures per day across 254 days per year (this is total number of working days Monday to Friday excluding public holidays in England). Serious adverse events that occurred before discharge were included in the bed day costs. Serious adverse events that required a hospital admission during follow-up were costed using the appropriate NHS tariff cost(31). Unit prices are reported in supplemental material S2.

Quality of life

Quality of life was measured using EQ-5D-5L QALYs. To calculate a QALY, results from the EQ-5D-5L were converted into health state utilities at baseline, 2, 4, 12 -weeks using a representative sample of the UK population(33). These utility values were then used to estimate QALYS at 12 weeks using the area under the curve approach(34).

Estimating Cost-effectiveness

A cost-effectiveness analysis at 12 weeks post-primary procedure was conducted to compare the incremental cost per quality adjusted life-year (QALY). The incremental cost-effectiveness ratio (ICER) was calculated as the difference in costs divided by the difference in QALYs between the two surgical groups using a bootstrap analysis with 1000 simulations. From this, a cost-effectiveness plane was produced, each quadrant indicating whether the surgical procedures were more or less expensive and more or less effective while also illustrating decision uncertainty(35). For outcomes in which an intervention is both more costly and provided more QALYs can be further evaluated through consideration of willingness to pay, the additional cost willing to be paid in order to gain an additional QALY. To do this, a cost-effectiveness acceptability curve (CEAC) was produced which shows the number of bootstrapped iterations in which RALS was considered cost-effective over CLS across a range willingness to pay of threshold values (£0 to £50 000 per additional QALY gained). In England, NICE typically use willingness to pay threshold values of £20 000 to £30 000 per QALY(36). These analyses were conducted using Stata 15.1. The cost effectiveness analysis was reported in

For peer review only - http://bmjopen.bmj.com/site/about/guidelines.xhtml

For peer review only

accordance to the Consolidated Health Economic Evaluation reporting Standards (CHEERS) statement(37).

One way sensitivity analysis was also conducted for the adjusted cost-effectiveness analysis to investigate how sensitive the results were to the assumptions for the annuitization and per-use cost of the capital equipment. This was conducted assuming different rates of daily usage (once, twice, and four times) across 254 days per year.

RESULTS

Participants

A total of 275 patients recruited to this study, 29 patients were excluded as they did not have procedures recorded. Of the remaining 246 patients, 168 underwent RALS, 78 underwent CLS. 9 RALS patients and 5 CLS patients were excluded due to missing all outcome data. Overall, 159 RALS and 73 CLS were eligible for analysis. A flow diagram is presented in supplemental material S3.

Baseline characteristics and surgical information of study participants are recorded in Table 1. The average age, BMI, smoking status, and number of prior surgeries were similar across both the RALS and CLS cohorts. However, there was a higher proportion of Caucasian patients in the CLS compared to the RALS cohort (n=68 (95.77%) versus n=129 (83.77%), p=0.011). The RALS cohort consisted of more patients with higher cancer grades (p=0.004) and higher cancer stages (p=0.012). There was no difference in EORTC or EQ-5D-5L health related quality of life scores at baseline between both cohorts. The CLS cohort travelled more distance in the 6MWT at baseline compared to the RALS patients (386.54 ± 109.71 versus 374.80 ± 365.30, p=0.454).

Table 1: Patient characteristics and surgical information

Descriptor	RALS (n=159)	CLS (n=73)	P
Demographics (baseline)			
**Age (years)**	61.5 ± 11.8	60.8 ± 12.2	0.690

For peer review only - http://bmjopen.bmj.com/site/about/guidelines.xhtml

**BMI** Mean ± SD	31.5 ± 8.0	30.6 ± 7.6	0.763
**Smoking Status**			0.849
Non-Smoker	116 (72.96%)	55 (75.34%)	
Ex-Smoker	6 (3.77%)	2 (2.74%)	
Smoker	26 (16.35%)	14 (19.18%)	
**Ethnicity**			0.011
White	129 (83.77%)	68 (93.15%)	
Non-White	25 (16.23%)	3 (4.11%)	
Unknown	11 (6.92%)	2 (2.74%)	
Medical history (baseline)			
**Number of prior surgeries**			0.476
0	129 (81.12%)	55 (75.34%)	
1	21 (15.72%)	15 (20.55%)	
2	8 (6.92%)	3 (4.11%)	
3	1 (0.63%)	0 (0%)	
Unknown	0 (0%)	0 (0%)	
**Histology**			0.145
Adenocarcinoma	15 (9.43%)	13 (17.81%)	
Aden squamous	2 (1.26%)	0 (0%)	
Benign	12 (7.55%)	10 (13.70%)	
Carcinoma sarcoma	5 (3.14%)	1 (1.37%)	
Clear cell	6 (3.77%)	1 (1.37%)	
Endometrioid	81 (50.94%)	36 (49.32%)	
Sarcoma	1 (0.63%)	1 (1.37%)	
Serous	24 (15.09%)	3 (4.11%)	
Squamous	6 (3.77%)	1 (1.37%)	
Unknown	7 (4.40%)	7 (9.59%)	
**Cancer Grade**			0.004
Benign	12 (7.55%)	10 (13.70%)	
1	41 (25.79%)	29 (39.73%)	
2	27 (16.98%)	11 (15.07%)	
3	46 (28.93%)	7 (9.59%)	
Unknown	33 (20.75%)	16 (21.92%)	
**Cancer Stage**			0.012
No Stage	12 (7.55)	10 (13.70%)	
1	110 (69.18%)	44 (60.27%)	
2	6 (3.77%)	8 (10.96%)	
3	15 (9.43%)	1 (1.37%)	
4	3 (1.89%)	0 (0%)	
Unknown	13 (8.18%)	10 (13.70%)	
Outcomes (baseline)			
**EORTC summary score**	86.29 ± 11. 38	86.79 ± 10.59	0.754
**EQ-5D-3L utility**	0.830 ± 0.150	0.800 ± 0.162	0.195
**6MWD (m)**	374.80 ± 365.30	386.54 ± 109.71	0.454
Surgical methods and length of stay			
**Lymph node dissection**			<0.001
Yes<br>*Full*<br>*Sentinel*	102 (64.14%)<br>*49*<br>*53*	6 (8.22%)<br>*3*<br>*3*	
No	57 (35.85%)	67 (91.78%)	

For peer review only - http://bmjopen.bmj.com/site/about/guidelines.xhtml

**Type of procedure**			0.166
Radical	16 (10.06%)	12 (16.44%)	
Simple	143 (89.94%)	61 (83.56%)	
**Type of hysterectomy**			0.263
With parametrium	18 (11.32%)	12 (16.44%)	
Without parametrium	141 (88.68%)	60 (82.19%)	
**Theatre times**	183 (68)	168 (93)	0.179
**Ward Days**	1.3 (2.1)	1.8 (2.4)	0.177
**ICU days**	0.08 (0.31)	0.07 (0.34)	0.774
**Total Length of Stay**	1.4 (2.1)	1.9 (2.4)	0.199
Data presented as n(%) or mean ± SD<br>CLS conventional laparoscopic surgery<br>RALS robotically assisted laparoscopic surgery			

Intra operative and length of stay

Very few patients had a lymph node dissection during CLS surgery compared to RALS (n=6 (8.22%) versus n=102 (64.14%), p=0.001). Of those who had a lymph node dissection in both surgeries, around half had a full lymph node dissection with the remainder having a sentinel procedure alone. The majority of both CLS and RALS procedures were simple hysterectomies (n= 61 (83.56%) and n= 143 (89.94%), p=0.166). Radical hysterectomy made up a very small proportion of patients for both procedures. Hysterectomy without parametrium were n= 60 (83.33%) and n= 141 (88.68%) (p=0.263) in CLS and RALS respectively. Theatre time was shorter on average for CLS (168 ± 93 minutes) compared to the RALS (183 ± 68 minutes) although this was not significant (p=0.179). Length of stay (LoS) in terms of general ward bed days was higher for CLS relative to RALS (1.8 ± 2.4 days versus 1.3 ± 2.1 days, p=0.177). Very few patients required an ICU stay for either surgical procedure (mean LOS of 0.07 ± 0.34 for CLS, 0.08 ± 0.31 for RALS, p=0.774). As such, total LoS was slightly higher on average for CLS compared to the RALS (1.9 ± 2.4 days versus 1.4 ± 2.1 days, p=0.199). There were no deaths in either cohort up until first discharge date.

Serious adverse events

There were 13 serious adverse events (SAE) recorded during this study (supplemental material S4), 6 occurred in CLS and 7 in RALS. For CLS, three occurred following the procedure but before discharge

For peer review only - http://bmjopen.bmj.com/site/about/guidelines.xhtml

with another three were detected over the first two weeks of follow-up. For RALS, there were 6 SAEs prior to discharge, and 1 SAE in week 2 of follow-up.

Primary Outcome

The crude and adjusted treatment effects for all outcomes are presented in Table 2. Mean EORTC health related quality of life at the 2-week follow-up compared to baseline fell for both RALS and CLS (-15.22 ± 13.82, -15.97 ± 15.28). The adjusted between-group difference (treatment effect) in EORTC from baseline to week 2 post-surgery was higher for RALS (1.6 (95% CI -2.8 to 6.1)). The mean EORTC at week 4 for both surgeries were also lower than baseline (-5.95 ± 13.31 for RALS and -4.49 ± 13.65 for CLS). The adjusted between group difference in the EORTC score from baseline to week 4 was lower for RALS (-0.3 (95% CI -4.8 to 4.1)). By 12 weeks follow-up, the EORTC score compared to baseline, had improved in both RALS (0.85 ± 11.36) and CLS (2.09 ± 11.45) respectively with an adjusted between group difference of 0.4 (95% CI -4.1 to 4.9) in favour of RALS.

Table 2: Crude change in outcomes from baseline at different follow-up points and estimated adjusted treatment effects with 95% C.I.

**Time-point**	**Number of participants**		**Crude change from baseline ***		**Estimated treatment effect (group difference) in mean change† with 95% C.I.**	**P-value**
	**RALS**	**CLS**	**RALS**	**CLS**		
Primary Outcome						
**EORTC score**						
2-week follow-up	114	55	-15.22 ± 13.82	-15.97 ± 15.28	1.6 (-2.8 to 6.1)	0.469
4-week follow-up	112	55	-5.95 ± 13.31	-4.49 ± 13.65	-0.3 (-4.8 to 4.1)	0.884
12-week follow-up	103	51	0.85 ± 11.36	2.09 ± 11.45	0.4 (-4.1 to 4.9)	0.861
Secondary Outcomes						
**6MWD (m)**						
2-week follow-up	86	52	-12.31 ± 84.05	-13.71 ± 104.04	18.1 (-11.0 to 47.2)	0.223
4-week follow-up	102	49	15.46 ± 71.03	31.08 ± 86.96	-3.4 (-33.2 to 26.4)	0.823
12-week follow-up	82	35	27.33 ± 86.94	32.48 ± 74.11	3.4 (-27.6 to 34.4)	0.830
**EQ-5D-3L utility**						

For peer review only - http://bmjopen.bmj.com/site/about/guidelines.xhtml

2-week follow-up	115	57	-0.11 ± 0.20	-0.09 ± 0.21	0.036 (-0.023 to 0.095)	0.227
4-week follow-up	114	55	-0.06 ± 0.16	-0.01 ± 0.20	0.010 (-0.050 to 0.069)	0.745
12-week follow-up	107	50	0.01 ± 0.16	0.02 ± 0.20	-0.002 (-0.061 to 0.058)	0.959

*Crude change from baseline given as mean change ± SD of this change
† Estimated treatment effect for all outcomes are the between group differences in the change from baseline derived from the mixed effects model adjusting for lymph node dissection and cancer stage as fixed effects
C.I. Confidence Interval, SD Standard Deviation, 6MWD six-minute walk distance, EORTC European Organisation for Research and Treatment of Cancer Quality of Life, CLS conventional laparoscopic surgery, RALS robotically assisted laparoscopic surgery

Secondary Outcomes

For both RALS and CLS, EQ-5D utility scores were lower in weeks 2 and 4 compared to baseline but were higher by week 12. The distance travelled in the 6MWT was lower for RALS and CLS in week 2 compared to baseline but was higher in weeks 4 and 12. However the adjusted analysis for both outcomes showed no statistically significant treatment effect.

Costs

Complete data was obtained for each participant regarding procedure costs and costs up until discharge. Summary costs of procedure and follow-up for both RALS and CLS are presented in supplemental material S5. Procedure costs included costs relating to staff, theatre time, equipment, and length of stay from admission to discharge. The mean procedure cost was higher for RALS compared to CLS (£4 758 ± 972 versus £3 741 ± 1 350, p<0.001). This was due to RALS having a higher mean staff costs (£1 851 ± 688 versus, £1 703 ± 940, p=0.231), theatre costs (£765 ± 284 versus £704 ± 388, p=0.231), and equipment costs (£2 134 versus £1 325). However, the mean cost for total LoS following the procedure was lower for RALS (£704 ± 1 002 versus £851 ± 1 159, p=0.352). Overall, the total mean cost from admission to discharge including the procedure costs were higher for RALS compared to CLS (£5 462 ± 1 472 versus £4 592 ± 2 072, p= 0.002).

For peer review only - http://bmjopen.bmj.com/site/about/guidelines.xhtml

Follow-up resource was collected up to 12 weeks post-discharge. In weeks 1-3 follow-up, post-discharge mean costs were higher for CLS relative to RALS, likely due to higher overnight stays in hospital. Thereafter, mean costs incurred in each week were similar between the two groups. Costs in week 12 were higher for RALS due to secondary care costs.

Cost-effectiveness analysis

Table 3 reports the outcomes of the constrained baseline longitudinal regression model unadjusted (no confounder) and adjusted cost-effectiveness analyses with Figure 1 presenting the cost-effectiveness acceptability curves and the willingness to pay.

Table 3: Results for the cost-effectiveness models

	Unadjusted Analysis	Adjusted Analysis ‡
Cost Difference * (£)	556 (-314 to 1 315)	-188 (-1 321 to 827)
QALYS Difference *	0.0024 (-0.0005 to 0.0057)	0.0024 (-0.0010 to 0.0057)
Non-parametric ICER (£ per QALY)	231 667	Dominated
Willingness to Pay (£20,000 threshold) †	0.117	0.657
Willingness to Pay (£25,000 threshold) †	0.126	0.664
Willingness to Pay (£30,000 threshold) †	0.133	0.671

Differences are shown as RALS – CLS.
* Reported as mean (95% confidence interval)
† Proportion of bootstrapped iterations in which RALS is cost-effective
‡ Models adjust for lymph node dissections and cancer stage
CLS conventional laparoscopic surgery, RALS robotically assisted laparoscopic surgery

For the unadjusted cost-effectiveness analysis, including follow-up over 12 weeks, the RALS cohort had a mean total cost difference of £556 (95%CI -£314 to £1 315) compared to CLS and a mean QALY difference of 0.0024 (95%CI -0.00051 to 0.0057). This resulted in a non-parametric ICER of £152 843

For peer review only - http://bmjopen.bmj.com/site/about/guidelines.xhtml

per QALY. At a willingness to pay threshold of £20 000 per QALY, 11.7% of the bootstrapped iterations resulted in RALS surgery being cost-effective, rising to 13.3% of iterations being cost-effective at £30 000 per QALY.

When adjusting for lymph node dissections and cancer stage, over 12 weeks, RALS had a mean cost difference of £-188 (95%CI -£1 321 to £827) compared to CLS and a mean QALY difference of 0.0024 (95%CI -0.0008 to 0.0057). This resulted in a non-parametric ICER being dominant. At a willingness to pay threshold of £20 000 per QALY, 65.7% of the bootstrapped iterations resulted in RALS being cost-effective, rising slightly higher to 67.1% of iterations being cost-effective at a £30 000 per QALY threshold.

Sensitivity analysis

The results of the one-way sensitivity analyses are presented in Table 4 with the cost-effectiveness plane, and willingness to pay figures presented supplemental material S6. RALS surgery had a higher average cost compared to CLS when assuming only 1 procedure per day (mean cost difference £430 (95%CI £-703 to £1 445)), with a resulting non-parametric ICER of £179 167 per QALY. When assuming only 1 procedure per day, only 23.8% of the bootstrapped iterations are cost-effective with a willingness to pay of £20 000 per QALY. When assuming 2 or 4 procedures per day, the cost of the robot is lower than CLS (mean cost difference: £-34 (95% CI £-1 167 to £981) and £-266 (£-1 399 to £749)) respectively. The non-parametric ICER for both scenarios were dominant in both cases, suggesting that RALS surgery provides more QALYs and has a lower cost compared to CLS. Assuming two procedures per day, RALS was cost-effective for 53.6% of bootstrapped iterations, and when assuming four procedures per day RALS was cost effective for 71.4% of the bootstrapped iterations at a willingness to pay of £20 000 per QALY.

Table 4: Results of the one-way sensitivity analysis of the adjusted cost-effectiveness model, varying the number of procedures performed per day.

	One procedure per day ‡	Two procedures per day ‡	Four procedures per day ‡

For peer review only - http://bmjopen.bmj.com/site/about/guidelines.xhtml

Cost Difference * (£)	430 (-703 to 1 445)	-34 (-1 167 to 981)	-266 (-1 399 to 749)
QALYS Difference *	0.0024 (-0.0010 to 0.0057)	0.0024 (-0.0010 to 0.0057)	0.0024 (-0.0010 to 0.0057)
Non-parametric ICER (£ per QALY)	179 167	Dominated	Dominated
Willingness to Pay (£20,000 threshold) †	0.238	0.536	0.700
Willingness to Pay (£25,000 threshold) †	0.246	0.553	0.703
Willingness to Pay (£30,000 threshold) †	0.256	0.569	0.714

Differences are shown as RALS – CLS.
* Reported as mean (95% confidence interval)
† Proportion of bootstrapped iterations in which RALS is cost-effective
‡ Models adjust for lymph node dissections and cancer stage
CLS conventional laparoscopic surgery, RALS robotically assisted laparoscopic surgery

DISCUSSION

The delivery of surgical care in gynaecological cancer in the UK National Health Service (NHS) continues to evolve. Minimal access surgery remains the mainstay for treating patients with endometrial cancer. Robotic surgery continues to be rolled out across NHS organisations but there is no national program to coordinate this, and the availability of robotic surgery is dependent on local trust finances and agreements with local commissioning groups(38). Thus, the type of surgical procedure a patient is offered to treat gynaecological malignancy is dependent upon their postcode and what is available in their local NHS hospital.

This pragmatic prospective comparative observational cohort study compares outcomes and cost effectiveness for patients undergoing conventional laparoscopic surgery or robotically assisted laparoscopic surgery reflecting real world care pathways. The results in terms of impact on functional outcomes and health related quality of life showed very little difference between the two

For peer review only - http://bmjopen.bmj.com/site/about/guidelines.xhtml

surgical methods and are in keeping with previous findings(39, 40). Therefore, the results of the cost-effectiveness analyses were largely driven by costs, including the cost of procurement and maintenance of the Robotic device and subsequent changes in resource use after surgery.

The unadjusted cost-effectiveness analysis in our study suggested that RALS was not cost-effective using the National Institute for Health and Care Excellence (NICE) threshold, of £30 000/€ 34 000(41). However, as with any observational study there were differences in characteristics of the patients in each of the surgical groups which led to confounding and bias in the unadjusted analysis. The characteristics in question were related to higher cancer stages and a higher number of lymph node evaluations in the RALS group. Explanation for these differences is difficult to attribute as one would expect patients to be treated by the same protocol according to the national guidelines which exist in England to direct practice. However, our results suggest that other factors come in to play when undertaking surgery, for example as body mass index rises surgical factors may influence what technically can be achieved between the two platforms.

Once these adjustments were made to the analysis, the probability of RALS being cost-effective was much higher. The difference in cost-effectiveness between the unadjusted and adjusted base case analysis was driven by cost differences. One possible explanation for this difference may be that the higher cancer stages and increased number of lymph node evaluations in the RALS cohort resulted in longer theatre times and subsequently higher theatre and staff costs.

Despite national guidelines(42) on best practice for lymph node evaluation, current practice varies across the UK dependent upon local resources and surgical paradigm. Further, we noted that some hysterectomies had been classified as ‘radical’, yet they had not had parametrial or lymph node dissections. Further analysis (data not shown) revealed that these patients had surgery for endometrial cancer rather than cervical cancer. The explanation for this lay in the way in that NHS Trusts coded their surgical procedures on the basis of commissioning because this had implications for payments to Trusts on procedures performed. For example, some operations were coded as a

For peer review only - http://bmjopen.bmj.com/site/about/guidelines.xhtml

modified radical hysterectomy if there was any element of ureterolysis. This meant that some endometrial cancers were described as being 'modified radical hysterectomy' but lymph node dissections may not have been indicated, as per local guidelines. The authors confirmed in those centres where this discrepancy was identified that all gynae oncologists would perform full bilateral pelvic lymphadenectomies for those patients having a radical hysterectomy for cervical cancer.

To our knowledge this study is the first to conduct a multi-center full cost-effectiveness evaluation comparing RALS versus CLS for hysterectomies following gynaecological cancer. Other studies in this area only focus on costs. An older prospective single center study comparing costs of RALS versus CLS was conducted by Sarlos, Kots (43). This study collected prospective data from June 2007 to May 2009 of 40 RALS patients following the introduction of robotic surgery in a single centre and compared this with a matched set of 40 CLS patients from retrospective records. Patients were matched 1:1 according to surgeon, uterus weight, BMI, and age. Unlike the findings of our adjusted analysis, Sarlos, Kots (43) estimated a higher cost of RALS £4066.84 compared to CLS £2150.76. However, we note that although the costs of Sarlos, Kots (43) are micro costed, they do not include the cost of the Robotic device. Furthermore, one potential driver of higher costs of RALS in their study could be due to surgeon inexperience. The RALS centres in our study include surgeons who were experienced in performing RALS and likely represents a truer reflection of practice today. As they conducted a matched case control study, Sarlos, Kots (43) do not present adjusted costs. However, we argue that there are potential limitations in the matching used as no consideration is given to patient comorbidities, which have been identified as drivers of cost (44).

Another study by Ind et al(45) investigated the costs of RALS compared CLS in a prospective cohort study of a single centre in England, where surgeons and surgical teams had prior experience with robotic surgery. It was noted that the robotic device was donated by a charity. Although the robotic device was donated to the centre, the micro costing conducted by Ind, Marshall (45) also included a depreciated per use cost of the purchase of the Robotic device. The results of Ind et al are similar to

For peer review only - http://bmjopen.bmj.com/site/about/guidelines.xhtml

our own study, with the 30-day cost of RALS estimated to be lower at £8 481 compared to CLS at £9 979. However, it should be noted that the cost estimates of both interventions are much higher than in our own study due to Ind, Marshall (45) reporting much higher theatre costs, length of stay, and high dependency use. For example, Ind, Marshall (45). report a median length of stay of 3 days for CLS and 2 days for RALS with 80.5% of CLS and 70.8% of RALS patients requiring high-dependency post-operative care. This differs from our own findings in which median length of stay for CLS and Robotic surgery was 1 day each, with very few patients in either arm requiring stay in ICU. Furthermore, length of time for surgery reported by Ind, Marshall (45) was higher for both interventions compared to our study. These differences may be due to factors such as changes in practice, advances in both RALS and CLS, or due to small sample size of Ind, Marshall (45).

A larger, more recent retrospective cohort study was conducted by Moss et al (44) comparing costs of different routes of surgery for the management of endometrial cancer with up to 1 year follow up using NHS England HES data between 2011-2018. The unadjusted cost at 90 days in the RALS cohort was on average £246 more expensive compared to CLS. However, the patients undergoing RALS had higher Charlson Comorbidity Index scores (CCI) and an increased number were obese compared to CLS. When controlling for the differences between the cohorts, Moss, Morgan (44) estimated a reduced cost difference at 90 days with RALS being only £89 more expensive. These results are similar to our own findings with the cost difference identified by Moss, Morgan (44) situated within the 95% confidence interval of the cost difference in our adjusted analysis £-188 95%CI (-1 321 to 827). However, there are still some limitations to their study compared to our own. Firstly, Moss, Morgan (44) utilised HRG tariff to estimate costs rather than detailed micro costing. Furthermore, unlike our detailed micro costing the HRG tariff estimates do not include capital costs or maintenance costs of the robotic devices, which as our study shows is a large and important component of surgical costs with robotic surgery. As a prospective multicentre cohort study, our study uses a much more rigorous inclusion criteria compared to Moss, Morgan (44) and data

For peer review only

For peer review only - http://bmjopen.bmj.com/site/about/guidelines.xhtml

collection of resource use may be more accurate in our study as we do not rely on HES data and also capture primary care resource use.

The sensitivity analysis conducted as part of our study identified that results of the cost-effectiveness analysis were highly sensitive to the volume of procedures performed. The finding that increasing the utilisation of the robotic devices increases the probability of RALS being cost effective is in keeping with cost-effectiveness analyses comparing robotic surgery in other pathologies(46). We therefore argue that the relationship between usage and cost-effectiveness of RALS should stimulate centres using robotic devices to maximise the volume of cases that are carried out using this equipment.

Acquisition of surgical robots has been managed in England to date by individual hospital trusts and a reliance on charitable donations with no centralised strategy. This may have unintended consequences for health service provision. Firstly, trusts may feel obligated to prioritise treatment of diseases on which the donating charity is focussed, even where this may not be cost-effective. Furthermore, we question the long-term viability of relying on charitable donations, particularly when the robotic devices become obsolete and reach the end of their life span and need to be replaced. Overreliance on charitable donations may not be sustainable into the future as RALS moves to becoming more mainstream.

Lam et al. examined the adoption of surgical robots through a nationwide Freedom of Information Request to all acute NHS trusts in England(38) and confirmed our argument regarding increasing the utilisation of the robotic equipment. Specifically, their results suggested that despite the number of robotic procedures increasing, national accessibility to robotic services and case volumes were highly variable with many trusts having low volume use representing missed efficiency savings for the NHS. We therefore argue that policies regarding the deployment and use of robotic surgical equipment are needed at national, regional and/or hospital level depending on the configuration of the health care system. One possible solution to this challenge in publicly funded national health systems such

For peer review only

For peer review only - http://bmjopen.bmj.com/site/about/guidelines.xhtml

as the U.K NHS is the centralisation of robotic centres into 'innovation hubs' than can establish best practice, clinical evidence, and patient volumes(38). In the absence of centralised initiatives, hospital operational policies should aim to procure robotic equipment over a range of surgical specialities parallel to a drive to undertake procedures in extended time slots to aid cost-effectiveness.

Limitations

We acknowledge the limitations of this study as a pragmatic prospective cohort study. This was necessary because of the current geography and delivery of RALS and CLS and the hub and spoke structure of cancer services in the UK. The fact that the two populations of patients differed in simple factors such as grade and stage of cancer has impacted the generalisability of this data. Although this study contained 12-week follow-up, in which there was no mortality recorded, it should be noted that stage differences may impact longer term mortality. Future studies which consider longer-term outcomes may need to consider the impact that stage differences may have on outcomes. Of some reassurance in terms of justifying robotic costs in endometrial cancer is the finding that the higher stage and grade patient cohort showed a swing in cost effectiveness favouring robotics despite worse prognostic factors. A randomised multi centre prospective trial would circumvent this limitation and provide data with greater generalisability to organisations wanting to establish the cost effectiveness of robotics ahead of implementation of a programme in their institution. However, design of such a trial would need significant and careful consideration to overcome the difficulties of acceptability to patients and surgeons alike, nationally and internationally, so that recruitment and ownership of the trial made it successful. In addition, the expansion of RALS across the NHS is advancing and could make it difficult to recruit enough CLS participants in any future trial, and indeed may have contributed towards the under recruitment of CLS patients in this study. Identifying cost-effectiveness through the gold standard RCT approach may not be feasible and as such this study is of great value in adding to the literature regarding the

For peer review only - http://bmjopen.bmj.com/site/about/guidelines.xhtml

cost-effectiveness comparison of RALS and CLS and helping to inform decision makers on efficient use of resources.

Although this study recruited a sufficient number of RALS patients, it struggled to recruit CLS patients and as such results of the primary analysis are underpowered. Attrition was lower than anticipated, however CLS still fell short of requirements. It should be noted however, that the cost-effectiveness analysis collected 'real world evidence' of costs and utilities for patients undergoing CLS and RALS, which will be informative and of great value to health care funders. Furthermore, cost-effectiveness analysis has long been recognised as being underpowered as a secondary outcome in many studies(47). The techniques used in this study follow recommended guidelines and focus on estimating the cost and effect differences alongside the likelihood of and intervention being cost-effective, rather than hypothesis testing, with consideration given to uncertainty around point estimates and comparisons(48).

This study did not include any cases which required conversion to open surgery. Future cost-effectiveness studies would benefit by accounting for these cases as they may impact cost-effectiveness results. A meta-analysis of robotic surgery(49) suggests that RALS for endometrial cancer offers lower conversion rates than CLS and as such it may be inferred that accounting for conversion cases may further increase the cost-effectiveness of RALS versus CLS.

A further limitation of this study is that confounding adjustment was managed within a regression model by including control variables. This required a well specified model to reduce residual confounding and consequent bias. Propensity scoring is an alternative and increasingly popular method of controlling potential confounding in observational studies that compare the effectiveness of healthcare interventions(50, 51). Propensity matching is typically used in cohort studies and involves fitting regression models to predict treatment groups based on selected characteristics(51). However, there is evidence to suggest that there is equivalence between confounding adjustment to various propensity score-based approaches(52, 53).

For peer review only - http://bmjopen.bmj.com/site/about/guidelines.xhtml

Conclusion

This study identified no differences in health related quality of life or functional outcomes between patients undergoing RALS or CLS for treatment of confirmed and suspected gynaecological endometrial and cervical cancers. However, this study has shown that there is evidence to suggest that RALS may be cost-effective compared to CLS, but results are highly sensitive to assumptions around the usage, and subsequent per use cost, of the robotic hardware. As robotic surgery becomes more mainstream, it is of vital importance that utilisation of robots are optimised to ensure efficient resource use. Further investigation is needed in this area is needed and a non-inferiority RCT would be of benefit in decision makers with regards to cost-effectiveness, however this may not be practical or achievable in the NHS.

Author Contributions

The study was conceptualisation by JG, PM, CN, MN, JL, JT. Data was curated by AM, DS, SM with formal analysis being conducted by AM, DS, JG, PM, SM. Funding was acquired by JG and JT. All authors were involved in the investigation and interpretation of data with methodology provided by AM, DS, JG, SM, PM. Original draft was written by AM and JG with all authors contributing to the review and editing. Final approval was obtained from all authors before submission. AM is the is the guarantor and attests that all listed authors meet authorship criteria and that no others meeting the criteria have been omitted.

Competing Interests

All authors have completed the ICMHE uniform disclosure form at http://www.icmje.org/disclosure-of-interest/ and declare: all authors had financial support from Intuitive for the submitted work. JL has received renumeration for time given as a proctor at intuitive, MN has received payment from intuitive for consultation and also for time given as a proctor and for development of educational lectures, in addition MN is President of the British & Irish Association of Robotic Gynaecological

For peer review only - http://bmjopen.bmj.com/site/about/guidelines.xhtml

For peer review only

Surgeons (BIARGS); no other relationships or activities that could appear to have influenced the submitted work.

Ethics

This NIHR Portfolio research study fully conformed to the ethical norms and standards in the Declaration of Helsinki. The study received the appropriate regulatory approvals in the UK before proceeding having been approved by an NHS Research Ethics Committee and registered with each participating NHS Trust as per their local Research department's process. Ethical approval was obtained from West Midlands Solihull Research Ethics Committee (NG1 6FSIRAS Ref 221315 - REC Ref - 17/WM/0056 R&D Approval date 22/06/2017). Furthermore, South Tees Hospitals NHS Foundation Trust as sponsor and trial coordinating organisation carried out several duties (such as provision of Site Initiation Visits and check-in calls, data validation reports, monitoring visits) to promote high quality trial conduct and adherence to Good Clinical Practice.

Acknowledgements

Sarah Kiddell (formerly Clinical Trial Practitioner for South Tees Hospitals NHS Foundation Trust) was the Trial Manager with primary responsibility for the day-to-day running of the study and reporting duties.

Richard Hodgson (formerly Research Data Officer for South Tees Hospitals NHS Foundation Trust), followed by Lesley Harris (formerly Research Data Officer for South Tees Hospitals NHS Foundation Trust) were the Data Support Officers checking data completeness and accuracy.

Julie Rowbotham (formerly Research Manager for South Tees Hospitals NHS Foundation Trust) acted as Sponsor Representative for the research study, followed by Joe Millar (Research Governance Manager for South Tees Hospitals NHS Foundation Trust). Both were responsible for overseeing the delivery of sponsorship responsibilities and providing research governance advice throughout.

For peer review only

For peer review only - http://bmjopen.bmj.com/site/about/guidelines.xhtml

The following contacts are all acknowledged for their role as Principal Investigator for each of the listed research sites:

Mr. Simon Butler-Manuel, Royal Surrey NHS Foundation Trust

Mr. Allan Gillespie, The Sheffield Teaching Hospitals NHS Foundation Trust

Mr. Pierre Martin-Hirsch, Lancashire Teaching Hospitals NHS Foundation Trust

Mr. Richard Edmondson, Manchester University NHS Foundation Trust

Mr. Mike Smith, The Christie NHS Foundation Trust

Mr. David Milliken, Somerset NHS Foundation Trust

Mr. Partha Sengupta, Country Durham & Darlington NHS Foundation Trust

Mr. Tony Challoub, Newcastle upon Tyne Hospitals NHS Foundation Trust

Data Sharing Statement

The complete research data supporting the findings of this paper were all stored in a web research database maintained by the funder Intuitive, which has now been data locked and exported as an excel record held by both Intuitive and the project team. Data is available on request due to privacy/ethical restrictions.

The corresponding author affirms that the manuscript is an honest, accurate, and transparent account of the study being reported. No important aspects of the study have been omitted and any discrepancies from the study as planned have been disclosed.

Funder Statement

The study was supported by Intuitive through a £93,850 funding grant and provision of a secure data management web service. Grant/award number: N/A. However, the study was managed and conducted by a wholly independent project team. The grant funding paid for identified project costs

For peer review only

such as project management staff support, statistician and health economist support, equipment, supplies and hospital staff support.

References

1. Press JZ, Gotlieb WH. Controversies in the treatment of early stage endometrial carcinoma. Obstet Gynecol Int. 2012;2012:578490.
2. Janda M, Gebski V, Brand A, Hogg R, Jobling TW, Land R, et al. Quality of life after total laparoscopic hysterectomy versus total abdominal hysterectomy for stage I endometrial cancer (LACE): a randomised trial. Lancet Oncol. 2010;11(8):772-80.
3. Kyrgiou M, Swart AM, Qian W, Warwick J. A Comparison of Outcomes Following Laparoscopic and Open Hysterectomy With or Without Lymphadenectomy for Presumed Early-Stage Endometrial Cancer: Results From the Medical Research Council ASTEC Trial. Int J Gynecol Cancer. 2015;25(8):1424-36.
4. Wright JD, Ananth CV, Lewin SN, Burke WM, Lu YS, Neugut AI, et al. Robotically assisted vs laparoscopic hysterectomy among women with benign gynecologic disease. Jama. 2013;309(7):689-98.
5. Matt A, Michelle, H. & Barry, O. C. . New developments Robot-assisted surgery in gynaecology Learning objectives : Ethical issues. 2011:183–8.
6. Boggess JF, Gehrig PA, Cantrell L, Shafer A, Ridgway M, Skinner EN, et al. A case-control study of robot-assisted type III radical hysterectomy with pelvic lymph node dissection compared with open radical hysterectomy. Am J Obstet Gynecol. 2008;199(4):357.e1-7.
7. Eklind S, Lindfors A, Sjöli P, Dahm-Kähler P. A prospective, comparative study on robotic versus open-surgery hysterectomy and pelvic lymphadenectomy for endometrial carcinoma. Int J Gynecol Cancer. 2015;25(2):250-6.
8. Patzkowsky KE, As-Sanie S, Smorgick N, Song AH, Advincula AP. Perioperative outcomes of robotic versus laparoscopic hysterectomy for benign disease. JSLS. 2013;17(1):100-6.
9. Sert BM, Boggess JF, Ahmad S, Jackson AL, Stavitzski NM, Dahl AA, et al. Robot-assisted versus open radical hysterectomy: A multi-institutional experience for early-stage cervical cancer. European Journal of Surgical Oncology (EJSO). 2016;42(4):513-22.
10. Desille-Gbaguidi H, Hebert T, Paternotte-Villemagne J, Gaborit C, Rush E, Body G. Overall care cost comparison between robotic and laparoscopic surgery for endometrial and cervical cancer. European Journal of Obstetrics & Gynecology and Reproductive Biology. 2013;171(2):348-52.
11. Liu H, Lawrie TA, Lu D, Song H, Wang L, Shi G. Robot-assisted surgery in gynaecology. Cochrane Database of Systematic Reviews. 2014(12).
12. England N. Clinical Commissioning Policy : Robotic-Assisted Surgical Procedures for Prostate Cancer. 2015. p. 1-14.
13. England N. 2015/16 Enhanced Tariff Option. 2015.
14. Calvert M, Blazeby J, Altman DG, Revicki DA, Moher D, Brundage MD. Reporting of patient-reported outcomes in randomized trials: the CONSORT PRO extension. Jama. 2013;309(8):814-22.
15. Centre HaSCI. Background information about PROMs. 2015.
16. Centre HaSCI. Outcomes Framework Indicators. 2015.
17. Herling SF, Møller AM, Palle C, Thomsen T. Health-related quality of life after robotic-assisted laparoscopic hysterectomy for women with endometrial cancer — A prospective cohort study. Gynecologic Oncology. 2016;140(1):107-13.
18. Kumar S, Paiz A, Santoso J, Tillmanns T. A retrospective study to compare quality of life in patients surgically treated by total abdominal hysterectomy versus robotic assisted hysterectomy for early stage endometrial cancer. Gynecologic Oncology. 2012;127(1):S24-S5.

For peer review only - http://bmjopen.bmj.com/site/about/guidelines.xhtml

19. Preston NJ, Wilson N, Wood NJ, Brine J, Ferreira J, Brearley SG. Patient-reported outcome measures for use in gynaecological oncology: a systematic review. Bjog. 2015;122(5):615-22.
20. Giesinger JM, Kieffer JM, Fayers PM, Groenvold M, Petersen MA, Scott NW, et al. Replication and validation of higher order models demonstrated that a summary score for the EORTC QLQ-C30 is robust. J Clin Epidemiol. 2016;69:79-88.
21. Schmidt K, Vogt L, Thiel C, Jäger E, Banzer W. Validity of the Six-Minute Walk Test in Cancer Patients. Int J Sports Med. 2013;34(07):631-6.
22. Holland AE, Spruit MA, Troosters T, Puhan MA, Pepin V, Saey D, et al. An official European Respiratory Society/American Thoracic Society technical standard: field walking tests in chronic respiratory disease. European Respiratory Journal. 2014;44(6):1428-46.
23. Laboratories ACoPSfCPF. ATS statement: guidelines for the six-minute walk test. Am J Respir Crit Care Med. 2002;166:111-7.
24. Rabin R, Gudex C, Selai C, Herdman M. From translation to version management: a history and review of methods for the cultural adaptation of the EuroQol five-dimensional questionnaire. Value Health. 2014;17(1):70-6.
25. Group TE. EuroQol--a new facility for the measurement of health-related quality of life. Health Policy. 1990;16(3):199-208.
26. von Elm E, Altman DG, Egger M, Pocock SJ, Gøtzsche PC, Vandenbroucke JP. The Strengthening the Reporting of Observational Studies in Epidemiology (STROBE) statement: guidelines for reporting observational studies. The Lancet. 2007;370(9596):1453-7.
27. Maringwa JT, Quinten C, King M, Ringash J, Osoba D, Coens C, et al. Minimal important differences for interpreting health-related quality of life scores from the EORTC QLQ-C30 in lung cancer patients participating in randomized controlled trials. Support Care Cancer. 2011;19(11):1753-60.
28. Coffman CJ, Edelman D, Woolson RF. To condition or not condition? Analysing 'change' in longitudinal randomised controlled trials. BMJ Open. 2016;6(12):e013096.
29. Bland JM, Altman DG. Regression towards the mean. Bmj. 1994;308(6942):1499.
30. Curtice L. BA. Unit Costs of Health and Social Care 2019. In: Unit PSSR, editor. Canterbury: University of Kent; 2019.
31. Care DoHaS. NHS Reference Costs 2018 to 2019. In: Care DoHaS, editor. London2019.
32. Walker D, Kumaranayake L. Allowing for differential timing in cost analyses: discounting and annualization. Health Policy Plan. 2002;17(1):112-8.
33. Szende A JB, Cabases J, editors. Self-Reported Population Health: An International Perspective based on EQ-5D. Dordecht (NL)2014.
34. Manca A, Hawkins N, Sculpher MJ. Estimating mean QALYs in trial-based cost-effectiveness analysis: the importance of controlling for baseline utility. Health Economics. 2005;14(5):487-96.
35. Fenwick E, Claxton K, Sculpher M. Representing uncertainty: the role of cost-effectiveness acceptability curves. Health Econ. 2001;10(8):779-87.
36. Excellence NIfHaC. Guide to the methofs of technology appraisal. London2018.
37. Husereau D, Drummond M, Augustovski F, de Bekker-Grob E, Briggs AH, Carswell C, et al. Consolidated Health Economic Evaluation Reporting Standards 2022 (CHEERS 2022) statement: updated reporting guidance for health economic evaluations. BMC Medicine. 2022;20(1):23.
38. Lam K, Clarke J, Purkayastha S, Kinross JM. Uptake and accessibility of surgical robotics in England. The International Journal of Medical Robotics and Computer Assisted Surgery. 2021;17(1):e2174.
39. Kim JY, Lee YH, Chong GO, Lee YS, Cho YL, Hong DG. Comparative Study Between Total Laparoscopic and Total Robotic Radical Hysterectomy for Cervical Carcinoma: Clinical Study. Anticancer Res. 2015;35(9):5015-21.
40. Roh HF, Nam SH, Kim JM. Robot-assisted laparoscopic surgery versus conventional laparoscopic surgery in randomized controlled trials: A systematic review and meta-analysis. PLoS One. 2018;13(1):e0191628-e.

For peer review only - http://bmjopen.bmj.com/site/about/guidelines.xhtml

41. McCabe C, Claxton K, Culyer AJ. The NICE Cost-Effectiveness Threshold. PharmacoEconomics. 2008;26(9):733-44.
42. Morrison J, Balega J, Buckley L, Clamp A, Crosbie E, Drew Y, et al. British Gynaecological Cancer Society (BGCS) uterine cancer guidelines: Recommendations for practice. European Journal of Obstetrics and Gynecology and Reproductive Biology. 2022;270:50-89.
43. Sarlos D, Kots L, Stevanovic N, Schaer G. Robotic hysterectomy versus conventional laparoscopic hysterectomy: Outcome and cost analyses of a matched case–control study. European Journal of Obstetrics and Gynecology and Reproductive Biology. 2010;150(1):92-6.
44. Moss EL, Morgan G, Martin A, Sarhanis P, Ind T. Economic evaluation of different routes of surgery for the management of endometrial cancer: a retrospective cohort study. BMJ Open. 2021;11(5):e045888.
45. Ind TEJ, Marshall C, Hacking M, Harris M, Bishop L, Barton D, et al. Introducing robotic surgery into an endometrial cancer service–a prospective evaluation of clinical and economic outcomes in a UK institution. The International Journal of Medical Robotics and Computer Assisted Surgery. 2016;12(1):137-44.
46. Labban M, Dasgupta P, Song C, Becker R, Li Y, Kreaden US, et al. Cost-effectiveness of Robotic-Assisted Radical Prostatectomy for Localized Prostate Cancer in the UK. JAMA Network Open. 2022;5(4):e225740-e.
47. Briggs A. Economic evaluation and clinical trials: size matters: the need for greater power in cost analyses poses an ethical dilemma. British Medical Journal Publishing Group; 2000. p. 1362-3.
48. Petrou S, Gray A. Economic evaluation alongside randomised controlled trials: design, conduct, analysis, and reporting. BMJ. 2011;342:d1548.
49. Ran L, Jin J, Xu Y, Bu Y, Song F. Comparison of Robotic Surgery with Laparoscopy and Laparotomy for Treatment of Endometrial Cancer: A Meta-Analysis. PLOS ONE. 2014;9(9):e108361.
50. Kuss O, Blettner M, Börgermann J. Propensity Score: an Alternative Method of Analyzing Treatment Effects. Dtsch Arztebl Int. 2016;113(35-36):597-603.
51. Freemantle N, Marston L, Walters K, Wood J, Reynolds MR, Petersen I. Making inferences on treatment effects from real world data: propensity scores, confounding by indication, and other perils for the unwary in observational research. Bmj. 2013;347:f6409.
52. Stürmer T, Schneeweiss S, Brookhart MA, Rothman KJ, Avorn J, Glynn RJ. Analytic strategies to adjust confounding using exposure propensity scores and disease risk scores: nonsteroidal antiinflammatory drugs and short-term mortality in the elderly. Am J Epidemiol. 2005;161(9):891-8.
53. Kurth T, Walker AM, Glynn RJ, Chan KA, Gaziano JM, Berger K, et al. Results of multivariable logistic regression, propensity matching, propensity adjustment, and propensity-based weighting under conditions of nonuniform effect. Am J Epidemiol. 2006;163(3):262-70.

Figure 1: Graph A: Cost-effectiveness plane for unadjusted analysis. Graph B: Willingness to pay for unadjusted analysis. Graph C: Cost-effectiveness plane for adjusted analysis. Graph D: Willingness to pay for adjusted analysis.

For peer review only - http://bmjopen.bmj.com/site/about/guidelines.xhtml

Figure 1: Graph A: Cost-effectiveness plane for unadjusted analysis. Graph B: Willingness to pay for unadjusted analysis. Graph C: Cost-effectiveness plane for adjusted analysis. Graph D: Willingness to pay for adjusted analysis.

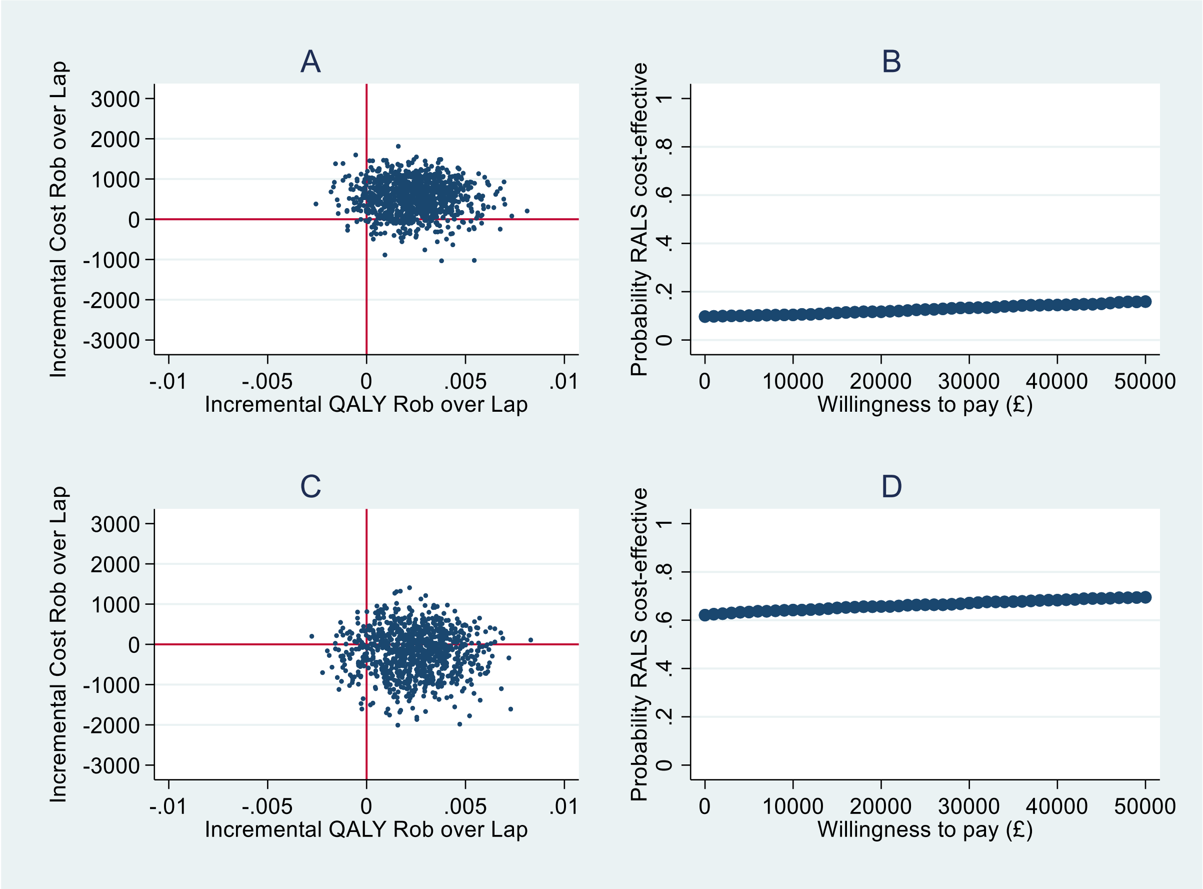

For peer review only

Supplemental material 1: Resource Use

Table S1.1: Resource use for surgical procedure

**Resource or unit intervention**	**Unit**	**Mean usage in standard laparoscopic**	**Mean usage in standard robotic**	**Resource source**
**Fixed theatre usage overheads**				
Operating theatre	Average theatre duration	168 minutes	183 minutes	RoBoQoL study procedure CRF form
**Staff**				
**Resource or unit intervention**	**Unit**	**Mean usage in standard laparoscopic**	**Mean usage in standard robotic**	**Resource source**
Consultant surgeon	Per hour	1	1	South Tees Hospitals NHS Foundation based on personal communication with Jeremy Twigg and Alison Waines between Jan-Nov 2020
Assistant surgeon (registrar)	Per hour	1	1	South Tees Hospitals NHS Foundation based on personal communication with Jeremy Twigg and Alison Waines between Jan-Nov 2020
Consultant anaesthetist	Per hour	1	1	South Tees Hospitals NHS Foundation based on personal communication with Jeremy Twigg and Alison Waines between Jan-Nov 2020
Anaesthetist registrar	Per hour	1	1	South Tees Hospitals NHS Foundation based on personal

For peer review only

				communication with Jeremy Twigg and Alison Waines between Jan-Nov 2020
Anaesthetist nurse	Per hour	1	1	South Tees Hospitals NHS Foundation based on personal communication with Jeremy Twigg and Alison Waines between Jan-Nov 2020
Nurse	Per hour	1	1	South Tees Hospitals NHS Foundation based on personal communication with Jeremy Twigg and Alison Waines between Jan-Nov 2020
Health care assistant	Per hour	1	1	South Tees Hospitals NHS Foundation based on personal communication with Jeremy Twigg and Alison Waines between Jan-Nov 2020
Recovery practitioner	Per hour	1	1	South Tees Hospitals NHS Foundation based on personal communication with Jeremy Twigg and Alison Waines between Jan-Nov 2020
**Capital equipment**				
**Resource or unit intervention**	**Unit**	**Mean usage in standard laparoscopic**	**Mean usage in standard robotic**	**Resource source**

For peer review only

Laparoscopic device (slave screens included)	Per procedure	1	1	South Tees Hospitals NHS Foundation based on personal communication with Jeremy Twigg and Alison Waines between Jan-Nov 2020
Robotic device	Per procedure	1	1	South Tees Hospitals NHS Foundation based on personal communication with Jeremy Twigg and Alison Waines between Jan-Nov 2020
Diathermy console	Per procedure	1	1	South Tees Hospitals NHS Foundation based on personal communication with Jeremy Twigg and Alison Waines between Jan-Nov 2020
Anaesthetic machine	Per procedure	1	1	South Tees Hospitals NHS Foundation based on personal communication with Jeremy Twigg and Alison Waines between Jan-Nov 2020
PCA pump	Per procedure	1	1	South Tees Hospitals NHS Foundation based on personal communication with Jeremy Twigg and Alison Waines between Jan-Nov 2020

For peer review only

Suction machine	Per procedure	1	1	South Tees Hospitals NHS Foundation based on personal communication with Jeremy Twigg and Alison Waines between Jan-Nov 2020
**Consumables<br>Surgical equipment specific to laparoscopic and robotic surgery**				
**Resource or unit intervention**	**Unit**	**Mean usage in standard laparoscopic**	**Mean usage in standard robotic**	**Resource source**
Gel port/trocar	Per procedure	1	n/a	South Tees Hospitals NHS Foundation based on personal communication with Jeremy Twigg and Alison Waines between Jan-Nov 2020
55mm dual port/trocar	Per procedure	4	n/a	South Tees Hospitals NHS Foundation based on personal communication with Jeremy Twigg and Alison Waines between Jan-Nov 2020
11mm port/trocar	Per procedure	1	n/a	South Tees Hospitals NHS Foundation based on personal communication with Jeremy Twigg and Alison Waines between Jan-Nov 2020
Verris lap pack	Per procedure	1	n/a	South Tees Hospitals NHS Foundation based on personal communication

For peer review only

				with Jeremy Twigg and Alison Waines between Jan-Nov 2020
Vcare manipulator	Per procedure	1	1	South Tees Hospitals NHS Foundation based on personal communication with Jeremy Twigg and Alison Waines between Jan-Nov 2020
Applied scissors	Per procedure	1	n/a	South Tees Hospitals NHS Foundation based on personal communication with Jeremy Twigg and Alison Waines between Jan-Nov 2020
Harmonic (ethicon)	Per procedure	1	n/a	South Tees Hospitals NHS Foundation based on personal communication with Jeremy Twigg and Alison Waines between Jan-Nov 2020
Misc. forceps	Per procedure	1	n/a	South Tees Hospitals NHS Foundation based on personal communication with Jeremy Twigg and Alison Waines between Jan-Nov 2020
Vlock suture	Per procedure	1	1	South Tees Hospitals NHS Foundation based on personal communication with Jeremy

For peer review only

				Twigg and Alison Waines between Jan-Nov 2020
Endoclose	Per procedure	1	1	South Tees Hospitals NHS Foundation based on personal communication with Jeremy Twigg and Alison Waines between Jan-Nov 2020
Syringes	Per procedure	1	1	South Tees Hospitals NHS Foundation based on personal communication with Jeremy Twigg and Alison Waines between Jan-Nov 2020
Scissors (robot specific)	Per procedure	1	1	South Tees Hospitals NHS Foundation based on personal communication with Jeremy Twigg and Alison Waines between Jan-Nov 2020
Pro-grasp	Per procedure	1	1	South Tees Hospitals NHS Foundation based on personal communication with Jeremy Twigg and Alison Waines between Jan-Nov 2020
Maryland bi-polar	Per procedure	1	1	South Tees Hospitals NHS Foundation based on personal communication with Jeremy Twigg and Alison

For peer review only

				Waines between Jan-Nov 2020
Needle holder	Per procedure	1	1	South Tees Hospitals NHS Foundation based on personal communication with Jeremy Twigg and Alison Waines between Jan-Nov 2020
Scope sterilisation for 30 degrees & 0 degrees	Per procedure	1	1	South Tees Hospitals NHS Foundation based on personal communication with Jeremy Twigg and Alison Waines between Jan-Nov 2020
Accessory set sterilisation	Per procedure	n/a	2	South Tees Hospitals NHS Foundation based on personal communication with Jeremy Twigg and Alison Waines between Jan-Nov 2020
**General consumable surgical equipment**				
**Resource or unit intervention**	**Unit**	**Mean usage in standard laparoscopic**	**Mean usage in standard robotic**	**Resource source**
Arterial line	Per procedure	1	1	South Tees Hospitals NHS Foundation based on personal communication with Jeremy Twigg, Alison Waines and Richard Bickle between Jan-Nov 2020
Central line	Per procedure	1	1	South Tees Hospitals NHS

For peer review only

				Foundation based on personal communication with Jeremy Twigg, Alison Waines and Richard Bickle between Jan-Nov 2020
12F catheter	Per procedure	1	1	South Tees Hospitals NHS Foundation based on personal communication with Jeremy Twigg, Alison Waines and Richard Bickle between Jan-Nov 2020
Urine drainage bag	Per procedure	1	1	South Tees Hospitals NHS Foundation based on personal communication with Jeremy Twigg, Alison Waines and Richard Bickle between Jan-Nov 2020
Monocryl	Per procedure	4	4	South Tees Hospitals NHS Foundation based on personal communication with Jeremy Twigg, Alison Waines and Richard Bickle between Jan-Nov 2020
Bair Hugger warmer	Per procedure	1	1	South Tees Hospitals NHS Foundation based on personal

For peer review only

				communication with Jeremy Twigg, Alison Waines and Richard Bickle between Jan-Nov 2020
Fluid warmer	Per procedure	1	1	South Tees Hospitals NHS Foundation based on personal communication with Jeremy Twigg, Alison Waines and Richard Bickle between Jan-Nov 2020
Drape	Per procedure	1	n/a	South Tees Hospitals NHS Foundation based on personal communication with Jeremy Twigg, Alison Waines and Richard Bickle between Jan-Nov 2020
4 arm drape	Per procedure	n/a	1	South Tees Hospitals NHS Foundation based on personal communication with Jeremy Twigg, Alison Waines and Richard Bickle between Jan-Nov 2020
**Scissor tip cover**	Per procedure	n/a	1	South Tees Hospitals NHS Foundation based on personal communication with Jeremy Twigg, Alison

For peer review only

				Waines and Richard Bickle between Jan-Nov 2020
**Blood products**				
**Resource or unit intervention**	**Unit**	**Mean usage in standard laparoscopic**	**Mean usage in standard robotic**	**Resource source**
Standard red blood cells	Per procedure	0.06 units	0.07 units	RoBoQoL study procedure CRF form
**Other resources**				
Anaesthetic equipment	Per procedure	1	1	South Tees Hospitals NHS Foundation based on personal communication with Richard Bickle between Jan-Nov 2020
Anaesthetic drugs	Per procedure	1	1	South Tees Hospitals NHS Foundation based on personal communication with Richard Bickle between Jan-Nov 2020
IV fluids	Per procedure	5	5	South Tees Hospitals NHS Foundation based on personal communication with Richard Bickle between Jan-Nov 2020
**Type of stay**				
ICU	Per day	0.07	0.08	RoBoQoL study procedure CRF
Ward	Per day	2.22	1.28	RoBoQoL study procedure CRF

Table S1.2: Resource use during follow-up

For peer review only

Resource or unit intervention		measure	RALS (n=159)	CLS (n=73)	Source
Week 1					
**Primary Care**					
General Practitioner (GP)	Home Visit	Mean (SD)	0.01 (0.09)	0 (0)	RoboQoL CRF
		Missing (N%)	39 (24.5)	6 (9)	
	Surgery Visit	Mean (SD)	0.13 (0.4)	0.05 (0.21)	RoboQoL CRF
		Missing (N%)	41 (25.8)	7 (10.6)	
	Phone Appointment	Mean (SD)	0.08 (0.3)	0.09 (0.29)	RoboQoL CRF
		Missing (N%)	40 (25.2)	6 (9)	
Nurse	Home Visit	Mean (SD)	0.16 (0.96)	0 (0)	RoboQoL CRF
		Missing (N%)	40 (25.2)	6 (9)	
	Surgery Visit	Mean (SD)	0.05 (0.22)	0.04 (0.27)	RoboQoL CRF
		Missing (N%)	41 (25.8)	6 (9)	
	Phone Appointment	Mean (SD)	0.03 (0.16)	0.03 (0.17)	RoboQoL CRF
		Missing (N%)	39 (24.5)	6 (9)	
Occupational Therapist (OT)	Home Visit	Mean (SD)	0 (0)	0 (0)	RoboQoL CRF
		Missing (N%)	39 (24.5)	7 (10.6)	
	Surgery Visit	Mean (SD)	0 (0)	0.05 (0.37)	RoboQoL CRF
		Missing (N%)	39 (24.5)	7 (10.6)	
	Hospital Visit	Mean (SD)	0 (0)	0 (0)	RoboQoL CRF
		Missing (N%)	39 (24.5)	7 (10.6)	
Physiotherapist	Home Visit	Mean (SD)	0 (0)	0 (0)	RoboQoL CRF
		Missing (N%)	39 (24.5)	6 (9)	
	Surgery Visit	Mean (SD)	39 (0)	6 (0)	RoboQoL CRF
		Missing (N%)	39 (24.5)	6 (9)	
	Phone Appointment	Mean (SD)	0.1 (0.4)	0.22 (0.42)	RoboQoL CRF
		Missing (N%)	39 (24.5)	6 (9)	
	Hospital Visit	Mean (SD)	0 (0)	0 (0)	RoboQoL CRF
		Missing (N%)	39 (24.5)	6 (9)	
	Day Visit	Mean (SD)	0 (0)	0 (0)	RoboQoL CRF
		Missing (N%)	39 (24.5)	6 (9)	
**Secondary Care**					
Outpatient Appointment		Mean (SD)	0.1 (0.1)	0.07 (0.07)	RoboQoL CRF
		Missing (N%)	39 (24.5)	6 (9)	
Ambulance See and treat		Mean (SD)	0.01 (0.09)	0 (0)	RoboQoL CRF
		Missing (N%)	39 (24.5)	6 (9)	
Ambulance see and convey		Mean (SD)	0 (0)	0 (0)	RoboQoL CRF
		Missing (N%)	39 (24.5)	6 (9)	
A&E		Mean (SD)	0.08 (0.66)	0.03 (0.17)	RoboQoL CRF
		Missing (N%)	41 (25.8)	6 (9)	
Hospital Ward Day		Mean (SD)	0.05 (0.26)	0.04 (0.21)	RoboQoL CRF
		Missing (N%)	41 (25.8)	6 (9)	
Hospital Overnight Visit		Mean (SD)	0.15 (0.65)	0.58 (1.55)	RoboQoL CRF
		Missing (N%)	40 (25.2)	6 (9)	

For peer review only

Week 2					
**Primary Care**					
General Practitioner (GP)	Home Visit	Mean (SD)	0.02 (0.18)	0 (0)	RoboQoL CRF
		Missing (N%)	42 (26.4)	3 (4.3)	
	Surgery Visit	Mean (SD)	0.07 (0.25)	0.04 (0.2)	RoboQoL CRF
		Missing (N%)	42 (26.4)	3 (4.3)	
	Phone Appointment	Mean (SD)	0.09 (0.35)	0.07 (0.26)	RoboQoL CRF
		Missing (N%)	42 (26.4)	3 (4.3)	
Nurse	Home Visit	Mean (SD)	0.14 (0.93)	0.01 (0.12)	RoboQoL CRF
		Missing (N%)	43 (27)	4 (5.8)	
	Surgery Visit	Mean (SD)	0.1 (0.31)	0.01 (0.12)	RoboQoL CRF
		Missing (N%)	43 (27)	6 (9)	
	Phone Appointment	Mean (SD)	0.03 (0.21)	0 (0)	RoboQoL CRF
		Missing (N%)	43 (27)	4 (5.8)	
Occupational Therapist (OT)	Home Visit	Mean (SD)	0 (0)	0 (0)	RoboQoL CRF
		Missing (N%)	43 (27)	4 (5.8)	
	Surgery Visit	Mean (SD)	0 (0)	0 (0)	RoboQoL CRF
		Missing (N%)	44 (27.7)	4 (5.8)	
	Hospital Visit	Mean (SD)	0 (0)	0 (0)	RoboQoL CRF
		Missing (N%)	43 (27)	4 (5.8)	
Physiotherapist	Home Visit	Mean (SD)	0 (0)	0 (0)	RoboQoL CRF
		Missing (N%)	42 (26.4)	4 (5.8)	
	Surgery Visit	Mean (SD)	42 (0)	4 (0)	RoboQoL CRF
		Missing (N%)	42 (26.4)	4 (5.8)	
	Phone Appointment	Mean (SD)	0 (0)	0.03 (0.17)	RoboQoL CRF
		Missing (N%)	42 (26.4)	4 (5.8)	
	Hospital Visit	Mean (SD)	0 (0)	0 (0)	RoboQoL CRF
		Missing (N%)	42 (26.4)	4 (5.8)	
	Day Visit	Mean (SD)	0 (0)	0 (0)	RoboQoL CRF
		Missing (N%)	42 (26.4)	4 (5.8)	
**Secondary Care**					
Outpatient Appointment		Mean (SD)	0.21 (0.21)	0.12 (0.12)	RoboQoL CRF
		Missing (N%)	39 (24.5)	4 (5.8)	
Ambulance See and treat		Mean (SD)	0.01 (0.09)	0.03 (0.17)	RoboQoL CRF
		Missing (N%)	42 (26.4)	3 (4.3)	
Ambulance see and convey		Mean (SD)	0.01 (0.09)	0.03 (0.17)	RoboQoL CRF
		Missing (N%)	42 (26.4)	3 (4.3)	
A&E		Mean (SD)	0.03 (0.18)	0.03 (0.17)	RoboQoL CRF
		Missing (N%)	42 (26.4)	3 (4.3)	
Hospital Ward Day		Mean (SD)	0.03 (0.16)	0.01 (0.12)	RoboQoL CRF
		Missing (N%)	42 (26.4)	4 (5.8)	
Hospital Overnight Visit		Mean (SD)	0.12 (0.71)	0.34 (1.38)	RoboQoL CRF
		Missing (N%)	42 (26.4)	3 (4.3)	
Week 3					
**Primary Care**					

For peer review only

General Practitioner (GP)	Home Visit	Mean (SD)	0 (0)	0 (0)	RoboQoL CRF
		Missing (N%)	44 (27.7)	11 (17.7)	
	Surgery Visit	Mean (SD)	0.11 (0.35)	0.06 (0.25)	RoboQoL CRF
		Missing (N%)	45 (28.3)	11 (17.7)	
	Phone Appointment	Mean (SD)	0.07 (0.29)	0.03 (0.18)	RoboQoL CRF
		Missing (N%)	44 (27.7)	11 (17.7)	
Nurse	Home Visit	Mean (SD)	0.14 (0.94)	0 (0)	RoboQoL CRF
		Missing (N%)	44 (27.7)	11 (17.7)	
	Surgery Visit	Mean (SD)	0.09 (0.34)	0.05 (0.22)	RoboQoL CRF
		Missing (N%)	44 (27.7)	11 (17.7)	
	Phone Appointment	Mean (SD)	0.03 (0.16)	0.03 (0.18)	RoboQoL CRF
		Missing (N%)	44 (27.7)	11 (17.7)	
Occupational Therapist (OT)	Home Visit	Mean (SD)	0 (0)	0 (0)	RoboQoL CRF
		Missing (N%)	44 (27.7)	11 (17.7)	
	Surgery Visit	Mean (SD)	0 (0)	0.03 (0.25)	RoboQoL CRF
		Missing (N%)	44 (27.7)	11 (17.7)	
	Hospital Visit	Mean (SD)	0 (0)	0 (0)	RoboQoL CRF
		Missing (N%)	44 (27.7)	11 (17.7)	
Physiotherapist	Home Visit	Mean (SD)	0 (0)	0 (0)	RoboQoL CRF
		Missing (N%)	45 (28.3)	11 (17.7)	
	Surgery Visit	Mean (SD)	45 (0)	11 (0)	RoboQoL CRF
		Missing (N%)	45 (28.3)	11 (17.7)	
	Phone Appointment	Mean (SD)	0.02 (0.13)	0 (0)	RoboQoL CRF
		Missing (N%)	45 (28.3)	11 (17.7)	
	Hospital Visit	Mean (SD)	0.01 (0.09)	0 (0)	RoboQoL CRF
		Missing (N%)	45 (28.3)	11 (17.7)	
	Day Visit	Mean (SD)	0 (0)	0 (0)	RoboQoL CRF
		Missing (N%)	45 (28.3)	11 (17.7)	
**Secondary Care**					
Outpatient Appointment		Mean (SD)	0.3 (0.3)	0.19 (0.19)	RoboQoL CRF
		Missing (N%)	43 (27)	10 (15.9)	
Ambulance See and treat		Mean (SD)	0 (0)	0 (0)	RoboQoL CRF
		Missing (N%)	45 (28.3)	11 (17.7)	
Ambulance see and convey		Mean (SD)	0 (0)	0 (0)	RoboQoL CRF
		Missing (N%)	45 (28.3)	11 (17.7)	
A&E		Mean (SD)	0.01 (0.09)	0 (0)	RoboQoL CRF
		Missing (N%)	45 (28.3)	11 (17.7)	
Hospital Ward Day		Mean (SD)	0 (0)	0.02 (0.13)	RoboQoL CRF
		Missing (N%)	45 (28.3)	11 (17.7)	
Hospital Overnight Visit		Mean (SD)	0.05 (0.26)	0.16 (1.15)	RoboQoL CRF
		Missing (N%)	45 (28.3)	11 (17.7)	
Week 4					
**Primary Care**					
General Practitioner (GP)	Home Visit	Mean (SD)	0.01 (0.09)	0.02 (0.13)	RoboQoL CRF
		Missing (N%)	44 (27.7)	10 (15.9)	

For peer review only

	Surgery Visit	Mean (SD)	0.08 (0.27)	0.1 (0.35)	RoboQoL CRF
		Missing (N%)	46 (28.9)	10 (15.9)	
	Phone Appointment	Mean (SD)	0.04 (0.2)	0.03 (0.25)	RoboQoL CRF
		Missing (N%)	44 (27.7)	10 (15.9)	
Nurse	Home Visit	Mean (SD)	0.09 (0.66)	0 (0)	RoboQoL CRF
		Missing (N%)	46 (28.9)	10 (15.9)	
	Surgery Visit	Mean (SD)	0.04 (0.25)	0.06 (0.25)	RoboQoL CRF
		Missing (N%)	46 (28.9)	10 (15.9)	
	Phone Appointment	Mean (SD)	0 (0)	0 (0)	RoboQoL CRF
		Missing (N%)	46 (28.9)	10 (15.9)	
Occupational Therapist (OT)	Home Visit	Mean (SD)	0 (0)	0 (0)	RoboQoL CRF
		Missing (N%)	45 (28.3)	10 (15.9)	
	Surgery Visit	Mean (SD)	0.01 (0.09)	0 (0)	RoboQoL CRF
		Missing (N%)	45 (28.3)	10 (15.9)	
	Hospital Visit	Mean (SD)	0 (0)	0 (0)	RoboQoL CRF
		Missing (N%)	45 (28.3)	10 (15.9)	
Physiotherapist	Home Visit	Mean (SD)	0 (0)	0 (0)	RoboQoL CRF
		Missing (N%)	47 (29.6)	10 (15.9)	
	Surgery Visit	Mean (SD)	47 (0)	10 (0)	RoboQoL CRF
		Missing (N%)	47 (29.6)	10 (15.9)	
	Phone Appointment	Mean (SD)	0 (0)	0 (0)	RoboQoL CRF
		Missing (N%)	48 (30.2)	10 (15.9)	
	Hospital Visit	Mean (SD)	0.01 (0.09)	0 (0)	RoboQoL CRF
		Missing (N%)	47 (29.6)	10 (15.9)	
	Day Visit	Mean (SD)	0 (0)	0 (0)	RoboQoL CRF
		Missing (N%)	47 (29.6)	10 (15.9)	
**Secondary Care**					
Outpatient Appointment		Mean (SD)	0.34 (0.34)	0.11 (0.11)	RoboQoL CRF
		Missing (N%)	46 (28.9)	8 (12.3)	
Ambulance See and treat		Mean (SD)	0.01 (0.09)	0 (0)	RoboQoL CRF
		Missing (N%)	45 (28.3)	9 (14.1)	
Ambulance see and convey		Mean (SD)	0 (0)	0 (0)	RoboQoL CRF
		Missing (N%)	45 (28.3)	9 (14.1)	
A&E		Mean (SD)	0.01 (0.09)	0 (0)	RoboQoL CRF
		Missing (N%)	45 (28.3)	9 (14.1)	
Hospital Ward Day		Mean (SD)	0 (0)	0.02 (0.13)	RoboQoL CRF
		Missing (N%)	45 (28.3)	9 (14.1)	
Hospital Overnight Visit		Mean (SD)	0.01 (0.09)	0.02 (0.13)	RoboQoL CRF
		Missing (N%)	45 (28.3)	9 (14.1)	
Week 5					
**Primary Care**					
General Practitioner (GP)	Home Visit	Mean (SD)	0 (0)	0 (0)	RoboQoL CRF
		Missing (N%)	56 (35.2)	14 (23.7)	
	Surgery Visit	Mean (SD)	0.1 (0.33)	0.07 (0.25)	RoboQoL CRF
		Missing (N%)	57 (35.8)	14 (23.7)	

	Phone Appointment	Mean (SD)	0.04 (0.2)	0.05 (0.22)	RoboQoL CRF
		Missing (N%)	57 (35.8)	14 (23.7)	
Nurse	Home Visit	Mean (SD)	0 (0)	0 (0)	RoboQoL CRF
		Missing (N%)	57 (35.8)	14 (23.7)	
	Surgery Visit	Mean (SD)	0.09 (0.32)	0.08 (0.28)	RoboQoL CRF
		Missing (N%)	57 (35.8)	14 (23.7)	
	Phone Appointment	Mean (SD)	0.01 (0.1)	0 (0)	RoboQoL CRF
		Missing (N%)	57 (35.8)	14 (23.7)	
Occupational Therapist (OT)	Home Visit	Mean (SD)	0 (0)	0 (0)	RoboQoL CRF
		Missing (N%)	57 (35.8)	15 (25.9)	
	Surgery Visit	Mean (SD)	0 (0)	0 (0)	RoboQoL CRF
		Missing (N%)	57 (35.8)	15 (25.9)	
	Hospital Visit	Mean (SD)	0 (0)	0 (0)	RoboQoL CRF
		Missing (N%)	57 (35.8)	15 (25.9)	
Physiotherapist	Home Visit	Mean (SD)	0 (0)	0 (0)	RoboQoL CRF
		Missing (N%)	56 (35.2)	15 (25.9)	
	Surgery Visit	Mean (SD)	56 (0)	15 (0)	RoboQoL CRF
		Missing (N%)	56 (35.2)	15 (25.9)	
	Phone Appointment	Mean (SD)	0 (0)	0 (0)	RoboQoL CRF
		Missing (N%)	56 (35.2)	15 (25.9)	
	Hospital Visit	Mean (SD)	0 (0)	0 (0)	RoboQoL CRF
		Missing (N%)	56 (35.2)	15 (25.9)	
	Day Visit	Mean (SD)	0 (0)	0 (0)	RoboQoL CRF
		Missing (N%)	56 (35.2)	15 (25.9)	
**Secondary Care**					
Outpatient Appointment		Mean (SD)	0.26 (0.26)	0.1 (0.1)	RoboQoL CRF
		Missing (N%)	57 (35.8)	14 (23.7)	
Ambulance See and treat		Mean (SD)	0 (0)	0 (0)	RoboQoL CRF
		Missing (N%)	59 (37.1)	14 (23.7)	
Ambulance see and convey		Mean (SD)	0 (0)	0 (0)	RoboQoL CRF
		Missing (N%)	59 (37.1)	14 (23.7)	
A&E		Mean (SD)	0.02 (0.14)	0 (0)	RoboQoL CRF
		Missing (N%)	56 (35.2)	14 (23.7)	
Hospital Ward Day		Mean (SD)	0 (0)	0.02 (0.13)	RoboQoL CRF
		Missing (N%)	56 (35.2)	14 (23.7)	
Hospital Overnight Visit		Mean (SD)	0 (0)	0.02 (0.13)	RoboQoL CRF
		Missing (N%)	56 (35.2)	14 (23.7)	
Week 6					
**Primary Care**					
General Practitioner (GP)	Home Visit	Mean (SD)	0 (0)	0.02 (0.13)	RoboQoL CRF
		Missing (N%)	55 (34.6)	14 (23.7)	
	Surgery Visit	Mean (SD)	0.16 (0.37)	0.09 (0.29)	RoboQoL CRF
		Missing (N%)	55 (34.6)	16 (28.1)	
	Phone Appointment	Mean (SD)	0.04 (0.19)	0.03 (0.18)	RoboQoL CRF
		Missing (N%)	55 (34.6)	13 (21.7)	

For peer review only

Nurse	Home Visit	Mean (SD)	0 (0)	0.03 (0.18)	RoboQoL CRF
		Missing (N%)	55 (34.6)	13 (21.7)	
	Surgery Visit	Mean (SD)	0.01 (0.1)	0.03 (0.18)	RoboQoL CRF
		Missing (N%)	55 (34.6)	14 (23.7)	
	Phone Appointment	Mean (SD)	0 (0)	0.05 (0.22)	RoboQoL CRF
		Missing (N%)	55 (34.6)	13 (21.7)	
Occupational Therapist (OT)	Home Visit	Mean (SD)	0 (0)	0 (0)	RoboQoL CRF
		Missing (N%)	55 (34.6)	13 (21.7)	
	Surgery Visit	Mean (SD)	0 (0)	0 (0)	RoboQoL CRF
		Missing (N%)	55 (34.6)	13 (21.7)	
	Hospital Visit	Mean (SD)	0 (0)	0 (0)	RoboQoL CRF
		Missing (N%)	55 (34.6)	13 (21.7)	
Physiotherapist	Home Visit	Mean (SD)	0 (0)	0 (0)	RoboQoL CRF
		Missing (N%)	55 (34.6)	13 (21.7)	
	Surgery Visit	Mean (SD)	55 (0)	13 (0)	RoboQoL CRF
		Missing (N%)	55 (34.6)	13 (21.7)	
	Phone Appointment	Mean (SD)	0 (0)	0 (0)	RoboQoL CRF
		Missing (N%)	55 (34.6)	13 (21.7)	
	Hospital Visit	Mean (SD)	0.01 (0.1)	0 (0)	RoboQoL CRF
		Missing (N%)	55 (34.6)	13 (21.7)	
	Day Visit	Mean (SD)	0 (0)	0 (0)	RoboQoL CRF
		Missing (N%)	55 (34.6)	13 (21.7)	
**Secondary Care**					
Outpatient Appointment		Mean (SD)	0.31 (0.31)	0.12 (0.12)	RoboQoL CRF
		Missing (N%)	55 (34.6)	13 (21.7)	
Ambulance See and treat		Mean (SD)	0 (0)	0 (0)	RoboQoL CRF
		Missing (N%)	55 (34.6)	13 (21.7)	
Ambulance see and convey		Mean (SD)	0 (0)	0 (0)	RoboQoL CRF
		Missing (N%)	55 (34.6)	13 (21.7)	
A&E		Mean (SD)	0 (0)	0.02 (0.13)	RoboQoL CRF
		Missing (N%)	56 (35.2)	13 (21.7)	
Hospital Ward Day		Mean (SD)	0 (0)	0.02 (0.13)	RoboQoL CRF
		Missing (N%)	56 (35.2)	13 (21.7)	
Hospital Overnight Visit		Mean (SD)	0 (0)	0.05 (0.39)	RoboQoL CRF
		Missing (N%)	56 (35.2)	13 (21.7)	
Week 7					
**Primary Care**					
General Practitioner (GP)	Home Visit	Mean (SD)	0 (0)	0.03 (0.18)	RoboQoL CRF
		Missing (N%)	54 (34)	13 (21.7)	
	Surgery Visit	Mean (SD)	0.08 (0.3)	0.09 (0.28)	RoboQoL CRF
		Missing (N%)	55 (34.6)	15 (25.9)	
	Phone Appointment	Mean (SD)	0.05 (0.26)	0.02 (0.13)	RoboQoL CRF
		Missing (N%)	56 (35.2)	13 (21.7)	
Nurse	Home Visit	Mean (SD)	0 (0)	0.03 (0.18)	RoboQoL CRF
		Missing (N%)	56 (35.2)	13 (21.7)	

	Surgery Visit	Mean (SD)	0.01 (0.1)	0.05 (0.22)	RoboQoL CRF
		Missing (N%)	57 (35.8)	15 (25.9)	
	Phone Appointment	Mean (SD)	0.01 (0.1)	0.02 (0.13)	RoboQoL CRF
		Missing (N%)	57 (35.8)	13 (21.7)	
Occupational Therapist (OT)	Home Visit	Mean (SD)	0 (0)	0 (0)	RoboQoL CRF
		Missing (N%)	57 (35.8)	13 (21.7)	
	Surgery Visit	Mean (SD)	0 (0)	0 (0)	RoboQoL CRF
		Missing (N%)	58 (36.5)	13 (21.7)	
	Hospital Visit	Mean (SD)	0 (0)	0 (0)	RoboQoL CRF
		Missing (N%)	58 (36.5)	13 (21.7)	
Physiotherapist	Home Visit	Mean (SD)	0 (0)	0 (0)	RoboQoL CRF
		Missing (N%)	55 (34.6)	13 (21.7)	
	Surgery Visit	Mean (SD)	55 (0)	13 (0)	RoboQoL CRF
		Missing (N%)	55 (34.6)	13 (21.7)	
	Phone Appointment	Mean (SD)	0 (0)	0 (0)	RoboQoL CRF
		Missing (N%)	55 (34.6)	13 (21.7)	
	Hospital Visit	Mean (SD)	0 (0)	0 (0)	RoboQoL CRF
		Missing (N%)	55 (34.6)	13 (21.7)	
	Day Visit	Mean (SD)	0 (0)	0 (0)	RoboQoL CRF
		Missing (N%)	55 (34.6)	13 (21.7)	
**Secondary Care**					
Outpatient Appointment		Mean (SD)	0.5 (0.5)	0.28 (0.28)	RoboQoL CRF
		Missing (N%)	56 (35.2)	13 (21.7)	
Ambulance See and treat		Mean (SD)	0 (0)	0 (0)	RoboQoL CRF
		Missing (N%)	55 (34.6)	13 (21.7)	
Ambulance see and convey		Mean (SD)	0 (0)	0 (0)	RoboQoL CRF
		Missing (N%)	55 (34.6)	13 (21.7)	
A&E		Mean (SD)	0 (0)	0 (0)	RoboQoL CRF
		Missing (N%)	56 (35.2)	13 (21.7)	
Hospital Ward Day		Mean (SD)	0.03 (0.17)	0 (0)	RoboQoL CRF
		Missing (N%)	56 (35.2)	13 (21.7)	
Hospital Overnight Visit		Mean (SD)	0 (0)	0 (0)	RoboQoL CRF
		Missing (N%)	56 (35.2)	13 (21.7)	
Week 8					
**Primary Care**					
General Practitioner (GP)	Home Visit	Mean (SD)	0 (0)	0.03 (0.18)	RoboQoL CRF
		Missing (N%)	57 (35.8)	13 (21.7)	
	Surgery Visit	Mean (SD)	0.08 (0.3)	0.16 (0.37)	RoboQoL CRF
		Missing (N%)	57 (35.8)	15 (25.9)	
	Phone Appointment	Mean (SD)	0.01 (0.1)	0.02 (0.13)	RoboQoL CRF
		Missing (N%)	58 (36.5)	13 (21.7)	
Nurse	Home Visit	Mean (SD)	0 (0)	0.02 (0.13)	RoboQoL CRF
		Missing (N%)	55 (34.6)	14 (23.7)	
	Surgery Visit	Mean (SD)	0.03 (0.17)	0.1 (0.31)	RoboQoL CRF
		Missing (N%)	57 (35.8)	15 (25.9)	

For peer review only

	Phone Appointment	Mean (SD)	0.02 (0.14)	0 (0)	RoboQoL CRF
		Missing (N%)	55 (34.6)	14 (23.7)	
Occupational Therapist (OT)	Home Visit	Mean (SD)	0 (0)	0 (0)	RoboQoL CRF
		Missing (N%)	56 (35.2)	14 (23.7)	
	Surgery Visit	Mean (SD)	0 (0)	0 (0)	RoboQoL CRF
		Missing (N%)	56 (35.2)	14 (23.7)	
	Hospital Visit	Mean (SD)	0 (0)	0 (0)	RoboQoL CRF
		Missing (N%)	56 (35.2)	14 (23.7)	
Physiotherapist	Home Visit	Mean (SD)	0 (0)	0 (0)	RoboQoL CRF
		Missing (N%)	57 (35.8)	14 (23.7)	
	Surgery Visit	Mean (SD)	57 (0)	14 (0)	RoboQoL CRF
		Missing (N%)	57 (35.8)	14 (23.7)	
	Phone Appointment	Mean (SD)	0 (0)	0 (0)	RoboQoL CRF
		Missing (N%)	57 (35.8)	14 (23.7)	
	Hospital Visit	Mean (SD)	0 (0)	0 (0)	RoboQoL CRF
		Missing (N%)	57 (35.8)	14 (23.7)	
	Day Visit	Mean (SD)	0 (0)	0 (0)	RoboQoL CRF
		Missing (N%)	57 (35.8)	14 (23.7)	
**Secondary Care**					
Outpatient Appointment		Mean (SD)	0.55 (0.55)	0.2 (0.2)	RoboQoL CRF
		Missing (N%)	56 (35.2)	13 (21.7)	
Ambulance See and treat		Mean (SD)	0 (0)	0 (0)	RoboQoL CRF
		Missing (N%)	57 (35.8)	14 (23.7)	
Ambulance see and convey		Mean (SD)	0 (0)	0 (0)	RoboQoL CRF
		Missing (N%)	57 (35.8)	14 (23.7)	
A&E		Mean (SD)	0 (0)	0 (0)	RoboQoL CRF
		Missing (N%)	57 (35.8)	14 (23.7)	
Hospital Ward Day		Mean (SD)	0.01 (0.1)	0 (0)	RoboQoL CRF
		Missing (N%)	57 (35.8)	14 (23.7)	
Hospital Overnight Visit		Mean (SD)	0.02 (0.2)	0 (0)	RoboQoL CRF
		Missing (N%)	57 (35.8)	14 (23.7)	
Week 9					
**Primary Care**					
General Practitioner (GP)	Home Visit	Mean (SD)	0 (0)	0 (0)	RoboQoL CRF
		Missing (N%)	55 (34.6)	13 (21.7)	
	Surgery Visit	Mean (SD)	0.08 (0.27)	0.1 (0.3)	RoboQoL CRF
		Missing (N%)	56 (35.2)	13 (21.7)	
	Phone Appointment	Mean (SD)	0.02 (0.14)	0 (0)	RoboQoL CRF
		Missing (N%)	55 (34.6)	13 (21.7)	
Nurse	Home Visit	Mean (SD)	0.01 (0.1)	0 (0)	RoboQoL CRF
		Missing (N%)	55 (34.6)	13 (21.7)	
	Surgery Visit	Mean (SD)	0.02 (0.14)	0.07 (0.25)	RoboQoL CRF
		Missing (N%)	55 (34.6)	13 (21.7)	
	Phone Appointment	Mean (SD)	0.01 (0.1)	0 (0)	RoboQoL CRF
		Missing (N%)	55 (34.6)	13 (21.7)	

For peer review only

Occupational Therapist (OT)	Home Visit	Mean (SD)	0 (0)	0 (0)	RoboQoL CRF
		Missing (N%)	56 (35.2)	13 (21.7)	
	Surgery Visit	Mean (SD)	0 (0)	0 (0)	RoboQoL CRF
		Missing (N%)	56 (35.2)	13 (21.7)	
	Hospital Visit	Mean (SD)	0 (0)	0 (0)	RoboQoL CRF
		Missing (N%)	56 (35.2)	13 (21.7)	
Physiotherapist	Home Visit	Mean (SD)	0 (0)	0 (0)	RoboQoL CRF
		Missing (N%)	56 (35.2)	13 (21.7)	
	Surgery Visit	Mean (SD)	56 (0)	13 (0)	RoboQoL CRF
		Missing (N%)	56 (35.2)	13 (21.7)	
	Phone Appointment	Mean (SD)	0 (0)	0 (0)	RoboQoL CRF
		Missing (N%)	56 (35.2)	13 (21.7)	
	Hospital Visit	Mean (SD)	0 (0)	0 (0)	RoboQoL CRF
		Missing (N%)	56 (35.2)	13 (21.7)	
	Day Visit	Mean (SD)	0 (0)	0 (0)	RoboQoL CRF
		Missing (N%)	56 (35.2)	13 (21.7)	
**Secondary Care**					
Outpatient Appointment		Mean (SD)	0.51 (0.51)	0.33 (0.33)	RoboQoL CRF
		Missing (N%)	57 (35.8)	13 (21.7)	
Ambulance See and treat		Mean (SD)	0 (0)	0 (0)	RoboQoL CRF
		Missing (N%)	56 (35.2)	14 (23.7)	
Ambulance see and convey		Mean (SD)	0 (0)	0 (0)	RoboQoL CRF
		Missing (N%)	56 (35.2)	14 (23.7)	
A&E		Mean (SD)	0 (0)	0 (0)	RoboQoL CRF
		Missing (N%)	56 (35.2)	13 (21.7)	
Hospital Ward Day		Mean (SD)	0 (0)	0.08 (0.65)	RoboQoL CRF
		Missing (N%)	56 (35.2)	13 (21.7)	
Hospital Overnight Visit		Mean (SD)	0.01 (0.1)	0.02 (0.13)	RoboQoL CRF
		Missing (N%)	56 (35.2)	13 (21.7)	
Week 10					
**Primary Care**					
General Practitioner (GP)	Home Visit	Mean (SD)	0 (0)	0 (0)	RoboQoL CRF
		Missing (N%)	56 (35.2)	13 (21.7)	
	Surgery Visit	Mean (SD)	0.06 (0.24)	0.07 (0.25)	RoboQoL CRF
		Missing (N%)	56 (35.2)	13 (21.7)	
	Phone Appointment	Mean (SD)	0.03 (0.17)	0 (0)	RoboQoL CRF
		Missing (N%)	56 (35.2)	13 (21.7)	
Nurse	Home Visit	Mean (SD)	0 (0)	0 (0)	RoboQoL CRF
		Missing (N%)	57 (35.8)	13 (21.7)	
	Surgery Visit	Mean (SD)	0.02 (0.14)	0.08 (0.28)	RoboQoL CRF
		Missing (N%)	57 (35.8)	13 (21.7)	
	Phone Appointment	Mean (SD)	0.03 (0.17)	0.02 (0.13)	RoboQoL CRF
		Missing (N%)	57 (35.8)	13 (21.7)	
Occupational Therapist (OT)	Home Visit	Mean (SD)	0 (0)	0 (0)	RoboQoL CRF
		Missing (N%)	57 (35.8)	13 (21.7)	

For peer review only

	Surgery Visit	Mean (SD)	0 (0)	0 (0)	RoboQoL CRF
		Missing (N%)	57 (35.8)	13 (21.7)	
	Hospital Visit	Mean (SD)	0 (0)	0 (0)	RoboQoL CRF
		Missing (N%)	57 (35.8)	13 (21.7)	
Physiotherapist	Home Visit	Mean (SD)	0 (0)	0.03 (0.26)	RoboQoL CRF
		Missing (N%)	57 (35.8)	13 (21.7)	
	Surgery Visit	Mean (SD)	57 (0)	13 (0)	RoboQoL CRF
		Missing (N%)	57 (35.8)	13 (21.7)	
	Phone Appointment	Mean (SD)	0.04 (0.31)	0 (0)	RoboQoL CRF
		Missing (N%)	57 (35.8)	13 (21.7)	
	Hospital Visit	Mean (SD)	0 (0)	0 (0)	RoboQoL CRF
		Missing (N%)	57 (35.8)	13 (21.7)	
	Day Visit	Mean (SD)	0 (0)	0 (0)	RoboQoL CRF
		Missing (N%)	57 (35.8)	13 (21.7)	
**Secondary Care**					
Outpatient Appointment		Mean (SD)	0.58 (0.58)	0.12 (0.12)	RoboQoL CRF
		Missing (N%)	58 (36.5)	13 (21.7)	
Ambulance See and treat		Mean (SD)	0 (0)	0.02 (0.13)	RoboQoL CRF
		Missing (N%)	57 (35.8)	13 (21.7)	
Ambulance see and convey		Mean (SD)	0 (0)	0.02 (0.13)	RoboQoL CRF
		Missing (N%)	57 (35.8)	13 (21.7)	
A&E		Mean (SD)	0.01 (0.1)	0.02 (0.13)	RoboQoL CRF
		Missing (N%)	57 (35.8)	14 (23.7)	
Hospital Ward Day		Mean (SD)	0.03 (0.17)	0.1 (0.66)	RoboQoL CRF
		Missing (N%)	57 (35.8)	13 (21.7)	
Hospital Overnight Visit		Mean (SD)	0 (0)	0.05 (0.39)	RoboQoL CRF
		Missing (N%)	57 (35.8)	13 (21.7)	
Week 11					
**Primary Care**					
General Practitioner (GP)	Home Visit	Mean (SD)	0 (0)	0 (0)	RoboQoL CRF
		Missing (N%)	56 (35.2)	13 (21.7)	
	Surgery Visit	Mean (SD)	0.06 (0.27)	0.1 (0.3)	RoboQoL CRF
		Missing (N%)	56 (35.2)	13 (21.7)	
	Phone Appointment	Mean (SD)	0.04 (0.2)	0.03 (0.18)	RoboQoL CRF
		Missing (N%)	57 (35.8)	13 (21.7)	
Nurse	Home Visit	Mean (SD)	0 (0)	0 (0)	RoboQoL CRF
		Missing (N%)	57 (35.8)	14 (23.7)	
	Surgery Visit	Mean (SD)	0.04 (0.24)	0.05 (0.22)	RoboQoL CRF
		Missing (N%)	57 (35.8)	14 (23.7)	
	Phone Appointment	Mean (SD)	0.03 (0.22)	0 (0)	RoboQoL CRF
		Missing (N%)	57 (35.8)	15 (25.9)	
Occupational Therapist (OT)	Home Visit	Mean (SD)	0 (0)	0 (0)	RoboQoL CRF
		Missing (N%)	56 (35.2)	14 (23.7)	
	Surgery Visit	Mean (SD)	0 (0)	0 (0)	RoboQoL CRF
		Missing (N%)	56 (35.2)	14 (23.7)	

For peer review only

	Hospital Visit	Mean (SD)	0 (0)	0 (0)	RoboQoL CRF
		Missing (N%)	56 (35.2)	14 (23.7)	
Physiotherapist	Home Visit	Mean (SD)	0 (0)	0 (0)	RoboQoL CRF
		Missing (N%)	56 (35.2)	14 (23.7)	
	Surgery Visit	Mean (SD)	56 (0)	14 (0)	RoboQoL CRF
		Missing (N%)	56 (35.2)	14 (23.7)	
	Phone Appointment	Mean (SD)	0 (0)	0 (0)	RoboQoL CRF
		Missing (N%)	56 (35.2)	14 (23.7)	
	Hospital Visit	Mean (SD)	0 (0)	0 (0)	RoboQoL CRF
		Missing (N%)	56 (35.2)	14 (23.7)	
	Day Visit	Mean (SD)	0 (0)	0 (0)	RoboQoL CRF
		Missing (N%)	56 (35.2)	15 (25.9)	
**Secondary Care**					
Outpatient Appointment		Mean (SD)	0.58 (0.58)	0.22 (0.22)	RoboQoL CRF
		Missing (N%)	58 (36.5)	14 (23.7)	
Ambulance See and treat		Mean (SD)	0.01 (0.1)	0.02 (0.13)	RoboQoL CRF
		Missing (N%)	56 (35.2)	14 (23.7)	
Ambulance see and convey		Mean (SD)	0.01 (0.1)	0.02 (0.13)	RoboQoL CRF
		Missing (N%)	56 (35.2)	14 (23.7)	
A&E		Mean (SD)	0.03 (0.22)	0.05 (0.39)	RoboQoL CRF
		Missing (N%)	57 (35.8)	14 (23.7)	
Hospital Ward Day		Mean (SD)	0 (0)	0.02 (0.13)	RoboQoL CRF
		Missing (N%)	57 (35.8)	14 (23.7)	
Hospital Overnight Visit		Mean (SD)	0 (0)	0.02 (0.13)	RoboQoL CRF
		Missing (N%)	57 (35.8)	14 (23.7)	
Week 12					
**Primary Care**					
General Practitioner (GP)	Home Visit	Mean (SD)	0.01 (0.1)	0 (0)	RoboQoL CRF
		Missing (N%)	51 (32.1)	15 (25.9)	
	Surgery Visit	Mean (SD)	0.06 (0.28)	0.05 (0.22)	RoboQoL CRF
		Missing (N%)	51 (32.1)	15 (25.9)	
	Phone Appointment	Mean (SD)	0.06 (0.31)	0.04 (0.19)	RoboQoL CRF
		Missing (N%)	51 (32.1)	16 (28.1)	
Nurse	Home Visit	Mean (SD)	0 (0)	0 (0)	RoboQoL CRF
		Missing (N%)	52 (32.7)	15 (25.9)	
	Surgery Visit	Mean (SD)	0.04 (0.23)	0.07 (0.26)	RoboQoL CRF
		Missing (N%)	52 (32.7)	15 (25.9)	
	Phone Appointment	Mean (SD)	0 (0)	0 (0)	RoboQoL CRF
		Missing (N%)	52 (32.7)	15 (25.9)	
Occupational Therapist (OT)	Home Visit	Mean (SD)	0 (0)	0 (0)	RoboQoL CRF
		Missing (N%)	53 (33.3)	15 (25.9)	
	Surgery Visit	Mean (SD)	0 (0)	0 (0)	RoboQoL CRF
		Missing (N%)	54 (34)	15 (25.9)	
	Hospital Visit	Mean (SD)	0 (0)	0 (0)	RoboQoL CRF
		Missing (N%)	54 (34)	15 (25.9)	

For peer review only

Physiotherapist	Home Visit	Mean (SD)	0 (0)	0.02 (0.13)	RoboQoL CRF
		Missing (N%)	53 (33.3)	15 (25.9)	
	Surgery Visit	Mean (SD)	53 (0)	15 (0)	RoboQoL CRF
		Missing (N%)	53 (33.3)	15 (25.9)	
	Phone Appointment	Mean (SD)	0 (0)	0 (0)	RoboQoL CRF
		Missing (N%)	53 (33.3)	15 (25.9)	
	Hospital Visit	Mean (SD)	0 (0)	0 (0)	RoboQoL CRF
		Missing (N%)	53 (33.3)	15 (25.9)	
	Day Visit	Mean (SD)	0 (0)	0 (0)	RoboQoL CRF
		Missing (N%)	53 (33.3)	15 (25.9)	
**Secondary Care**					
Outpatient Appointment		Mean (SD)	0.7 (0.7)	0.29 (0.29)	RoboQoL CRF
		Missing (N%)	54 (34)	15 (25.9)	
Ambulance See and treat		Mean (SD)	0 (0)	0 (0)	RoboQoL CRF
		Missing (N%)	53 (33.3)	15 (25.9)	
Ambulance see and convey		Mean (SD)	0 (0)	0 (0)	RoboQoL CRF
		Missing (N%)	53 (33.3)	15 (25.9)	
A&E		Mean (SD)	0 (0)	0 (0)	RoboQoL CRF
		Missing (N%)	53 (33.3)	15 (25.9)	
Hospital Ward Day		Mean (SD)	0.08 (0.62)	0 (0)	RoboQoL CRF
		Missing (N%)	53 (33.3)	15 (25.9)	
Hospital Overnight Visit		Mean (SD)	0 (0)	0 (0)	RoboQoL CRF
		Missing (N%)	53 (33.3)	15 (25.9)	

For peer review only

Supplemental Material S2: unit costs

Table S2.1 Unit costs of resources used during the surgical procedure including length of stay

**Resource or unit intervention**	**Laparoscopic**	**Robotic**	**Cost per unit (£)**	**Cost Source**
**Fixed costs of overheads**				
Operating theatre	Yes	Yes	250.00	South Tees Hospitals NHS Foundation based on personal communication with Alison Waines between Jan-Nov 2020
**Staff**				
**Resource or unit intervention**	**Laparoscopic**	**Robotic**	**Cost per unit (£)**	**Cost Source**
Consultant surgeon	Yes	Yes	109.00	PSSRU 2018/2019 based on "Consultant Surgical"
Assistant surgeon (registrar)	Yes	Yes	47.00	PSSRU 2018/2019 based on "Registrar"
Consultant anaesthetist	Yes	Yes	109.00	PSSRU 2018/2019 based on "Consultant medical"
Anaesthetist registrar	Yes	Yes	47.00	PSSRU 2018/2019 based on "Registrar"
Anaesthetist nurse	Yes	Yes	38.00	PSSRU 2018/2019 based on "hospital based nurses" (Band 5)
Nurse	Yes	Yes	38.00	PSSRU 2018/2019 based on "hospital based nurses" (Band 5)
Healthcare assistant	Yes	Yes	8.93	NHS Employers website 2018/2019 hourly rate (Band 2 with 3-4 years of experience)
Recovery practitioner	Yes	Yes	38.00	PSSRU 2018/2019 based on "hospital based nurses" (Band 5)
**Capital equipment**				
			**Cost (£)**	**Cost Source**

For peer review only

Resource or unit intervention	Laparoscopic	Robotic	Capital (life expectancy)	Annualised cost	Per case	South Tees Hospitals NHS Foundation based on personal communication with Alison Waines between Jan-Nov 2020
Laparoscopic device (screens included)	Yes	n/a	90 000.00 (8 years)	1 ,092.90	£17.25	KARL STORZ Endoscopy (UK) Ltd, based on personal communication with Matthew Taylor (Endoscopic sales specialist) in August 2020
Robotic device	n/a	Yes	1 000 300.00 (8 years)	145 520.29	191.73	South Tees Hospitals NHS Foundation based on personal communication with Jennifer Donnelly between Jan-Nov 2020
Robotic device maintenance cost	n/a	Yes	100 000.00 (per annum)	395.26	131.75	South Tees Hospitals NHS Foundation based on personal communication with Jeremy Twigg between Jan-Nov 2020
Diathermy console	Yes	Yes	10 800.75 (10 years)	1 298.70	1.71	South Tees Hospitals NHS Foundation based on personal communication with Jeremy Twigg between Jan-Nov 2020
Anaesthetic machine	Yes	Yes	20 000.00 (8 years)	2 909.53	3.83	South Tees Hospitals NHS Foundation based on personal communication with Jennifer Donnelly between Jan-Nov 2020
PCA pump	Yes	Yes	5 000.00 (5 years)	1 107.41	1.46	South Tees Hospitals NHS Foundation based on personal communication with Jennifer Donnelly between Jan-Nov 2020
Suction machine	Yes	Yes	2 000 (5 years)	442.96	0.58	South Tees Hospitals NHS Foundation based on personal communication with Jennifer Donnelly between Jan-Nov 2020
All annualised costs above have been annualised based on calculated annualisation factors, days worked per year is assumed as 253 days and equipment used on average three times per day.						
**Consumables<br>Surgical equipment specific to laparoscopic and robotic surgery**						
**Resource or unit intervention**	**Laparoscopic**	**Robotic**	**Cost per unit (£)**			**Cost Source**
Gel port/trocar	Yes	n/a	26.00			South Tees Hospitals NHS Foundation based on personal communication with Alison Waines between Jan-Nov 2020
55mm dual port/trocar	Yes	n/a	96.00			South Tees Hospitals NHS Foundation based on personal communication with Alison Waines between Jan-Nov 2020

For peer review only

11mm port/trocar	Yes		24.00	South Tees Hospitals NHS Foundation based on personal communication with Alison Waines between Jan-Nov 2020
Verris lap pack	Yes	n/a	5.00	South Tees Hospitals NHS Foundation based on personal communication with Alison Waines between Jan-Nov 2020
Vcare manipulator	Yes	1	50.00	South Tees Hospitals NHS Foundation based on personal communication with Alison Waines between Jan-Nov 2020
Applied scissors	Yes	n/a	20.00	South Tees Hospitals NHS Foundation based on personal communication with Alison Waines between Jan-Nov 2020
Harmonic (ethicon)	Yes	n/a	550.00	South Tees Hospitals NHS Foundation based on personal communication with Alison Waines between Jan-Nov 2020
Misc. forceps	Yes	n/a	100.00	South Tees Hospitals NHS Foundation based on personal communication with Alison Waines between Jan-Nov 2020
Vlock suture	Yes	Yes	25.00	South Tees Hospitals NHS Foundation based on personal communication with Alison Waines between Jan-Nov 2020
Endoclose	Yes	Yes	10.00	South Tees Hospitals NHS Foundation based on personal communication with Alison Waines between Jan-Nov 2020
Syringes	Yes	Yes	0.20	South Tees Hospitals NHS Foundation based on personal communication with Alison Waines between Jan-Nov 2020
Scissors (robot specific)	n/a	Yes	342.00	South Tees Hospitals NHS Foundation based on personal communication with Alison Waines between Jan-Nov 2020
Pro-grasp	n/a	Yes	234.96	South Tees Hospitals NHS Foundation based on personal communication with Alison Waines between Jan-Nov 2020
Maryland bi-polar	n/a	Yes	289.20	South Tees Hospitals NHS Foundation based on personal communication with Alison Waines between Jan-Nov 2020
Needle holder	n/a	Yes	234.96	South Tees Hospitals NHS Foundation based on personal

				communication with Alison Waines between Jan-Nov 2020
Scope sterilisation for 30 degrees & 0 degrees	n/a	Yes	220.00	South Tees Hospitals NHS Foundation based on personal communication with Alison Waines between Jan-Nov 2020
Accessory set sterilisation	n/a	Yes	100.00	South Tees Hospitals NHS Foundation based on personal communication with Alison Waines between Jan-Nov 2020
**General consumable surgical equipment**				
**Resource or unit intervention**	**Laparoscopic**	**Robotic**	**Cost per unit (£)**	**Cost Source**
Arterial line	Yes	Yes	45.00	South Tees Hospitals NHS Foundation based on personal communication with Alison Waines and Richard Bickle between Jan-Nov 2020
Central line	Yes	Yes	140.00	South Tees Hospitals NHS Foundation based on personal communication with Alison Waines and Richard Bickle between Jan-Nov 2020
12F catheter	Yes	Yes	3.00	South Tees Hospitals NHS Foundation based on personal communication with Alison Waines and Richard Bickle between Jan-Nov 2020
Urine drainage bag	Yes	Yes	1.00	South Tees Hospitals NHS Foundation based on personal communication with Alison Waines and Richard Bickle between Jan-Nov 2020
Monocryl	Yes	Yes	48.00	South Tees Hospitals NHS Foundation based on personal communication with Alison Waines and Richard Bickle between Jan-Nov 2020
Bair Hugger warmer	Yes	Yes	6.00	South Tees Hospitals NHS Foundation based on personal communication with Alison Waines and Richard Bickle between Jan-Nov 2020
Fluid warmer	Yes	Yes	4.00	South Tees Hospitals NHS Foundation based on personal communication with Alison

				Waines and Richard Bickle between Jan-Nov 2020
Drape	1	n/a	22.30	South Tees Hospitals NHS Foundation based on personal communication with Alison Waines between Jan-Nov 2020
4 arm drape	n/a	Yes	276.00	South Tees Hospitals NHS Foundation based on personal communication with Alison Waines between Jan-Nov 2020
Scissor tip cover	n/a	1	18.15	South Tees Hospitals NHS Foundation based on personal communication with Alison Waines between Jan-Nov 2020
**Blood products**				
**Resource or unit intervention**	**Laparoscopic**	**Robotic**	**Cost per unit (£)**	**Cost Source**
Standard red blood cells	Yes	n/a	128.99	NHS Blood and Transplant price 2018/19
**Other resources**				
**Resource or unit intervention**	**Laparoscopic**	**Robotic**	**Cost per unit (£)**	**Cost Source**
Anaesthetic equipment	Yes	Yes	18.00	South Tees Hospitals NHS Foundation based on personal communication with Richard Bickle between Jan-Nov 2020
Anaesthetic drugs	Yes	Yes	47.00	South Tees Hospitals NHS Foundation based on personal communication with Richard Bickle between Jan-Nov 2020
IV fluids	Yes	Yes	5.50	South Tees Hospitals NHS Foundation based on personal communication with Richard Bickle between Jan-Nov 2020
**Type of stay**				
ICU	Yes	Yes	1 417.63	Fromhttps://www.nice.org.uk/guidance/GID-NG10072/documents/evidence-review-12inflated using PSSRU
Ward day	Yes	Yes	£416.90	From https://www.nice.org.uk/guidance/GID-NG10072/documents/evidence-

For peer review only

				review-12, inflated using PSSRU index

Table S2.2 Unit costs for NHS resources used during follow-up

Resource	Cost (£)	Source
GP Home	100.62	From Unit Costs of Health and Social Care 2019, a GP costs £4.30 per minute.<br>Unit Costs of Health and Social Care 2015, the average home visit is 11.4 minutes with 12 minutes of travel time.
GP Surgery	39	Costs from Unit Costs of Health and Social Care 2019
GP Phone	15.32	Costs from Unit Costs of Health and Social Care 2019
Nurse Home	16.38	Costs from Unit Costs of Health and Social Care 2019<br>We assumed the travel and contact time was the same as GP home visit.
Nurse Surgery	10.85	Costs from Unit Costs of Health and Social Care 2019<br>From Unit Costs of Health and Social Care 2015, the average surgery contact time was 15.5 minutes.
Nurse Phone	7.8	Cost from Unit Costs of Health and Social Care 2019
OT Home	46.4	Costs from Unit Costs of Health and Social Care 2019<br>From Unit Costs of Health and Social Care 2010 , an average OT session is 40 minutes.<br>We assumed the travel time was the same as a GP.
OT Surgery	32	Costs from Unit Costs of Health and Social Care 2019<br>From Unit Costs of Health and Social Care 2010 , an average OT session is 40 minutes.
OT Hospital	70	Costs from Unit Costs of Health and Social Care 2019
Physio Home	34.23	Costs from Unit Costs of Health and Social Care 2019, we assumed the average between bands 5 and 6.<br>We assumed same length appointment as OT (40 minutes).<br>We assumed the same travel time as GP.
Physio Surgery	26.33	Costs from Unit Costs of Health and Social Care 2019, we assumed the average between bands 5 and 6.<br>We assumed same length appointment as OT (40 minutes).
Physio Phone	4.56	Costs from Unit Costs of Health and Social Care 2019, we assumed the average between bands 5 and 6.<br>From Unit Costs of Health and Social Care 2019, we assumed same length phone call as a nurse.
Physio Hospital	58	Costs from Unit Costs of Health and Social Care 2019.
Physio Day Ward	58	Cost from Unit Costs of Health and Social Care 2019.
Outpatient	135	Cost from Unit Costs of Health and Social Care 2019.
Emergency Ambulance	196	Cost from Unit Costs of Health and Social Care 2019. We assumed the costs for "See and treat and refer"
Emergency Ambulance Hospital	258	Cost from Unit Costs of Health and Social Care 2019. We assumed the costs for "See and treat and convey"

For peer review only

Hospital A&E	135.8	Costs from NHS Reference costs 2018/2019, we assumed Accident and Emergency
Hospital Day Ward	416.9	Cost from https://www.nice.org.uk/guidance/GID-NG10072/documents/evidence-review-12. This was inflated to 2019 prices using Unit Costs of Health and Social Care 2019.
Hospital Overnight ward	416.9	Cost from https://www.nice.org.uk/guidance/GID-NG10072/documents/evidence-review-12. This was inflated to 2019 prices using Unit Costs of Health and Social Care 2019.
**Serious Adverse Events**		
Resource	Unit Cost (£)	Source
Sepsis	2 205	Weighted average of NHS tariff HRG codes: WJ06A, WJ06B, WJ06C, WJ06D, WJ06E, WJ06F, WJ06G, WJ06H, WJ06J
Bowel obstruction	3 340	Weighted average of NHS tariff HRG codes: FF61A, FF61B, FF61C
Embolism/CVA	3 334	Weighted average of NHS tariff HRG codes: AA35A, AA35B, AA35C, AA35D, AA35E, AA35F

For peer review only

Supplemental material S3: Patient flow diagram

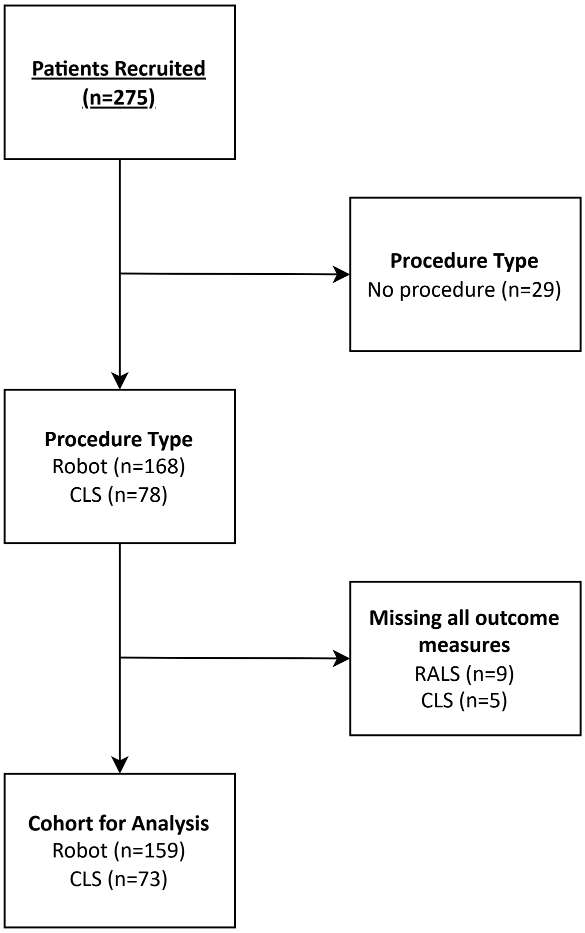

Supplemental Material S4

Table S4.1 list of serious adverse events

Serious Adverse event	Event time	RALS (n=159)	CLS (n=73)
Abdominal infection	Post-procedure	0	1
Accidental dislodgement of a needle	Post-procedure	0	1
CVA and DVT	Post-procedure	1	0
Haematoma with active bleeding	Post-procedure	1	0
lower suprapubic abdominal pain	Post-procedure	1	0
Nausea and vomiting	Post-procedure	2	0
Nervous system disorders	Post-procedure	1	0
Vaginal prolapse	Post-procedure	0	1
Sepsis	Week-1	0	2
Bowel obstruction	Week-2	0	1
CVA	Week-2	1	0

For peer review only

Supplemental material S5

S5.1: Procedure and post-procedure to discharge cost summaries for CLS and RALS patients.

Cost component	RALS (n=159)	CLS (n=73)	P
**Procedure Costs**			
**Staff cost**			**0.231**
Mean ± SD	1 851 (688)	1 703 (940)	
Median (IQR)	1 733 (1 068 to 2 278)	1 522 (987 to 2 117)	
**Theatre cost**			**0.231**
Mean ± SD	765 (284)	704 (388)	
Median (IQR)	716 (537 to 941)	630 (408 to 875)	
**Blood Products Cost**			**0.848**
Mean ± SD	7 (42)	9 (62)	
Median (IQR)	0 (0 to 0)	0 (0 to 0)	
**Equipment cost***	2 134	1 325	
**Total Procedure Cost**			**<0.001**
Mean ± SD	4 758 (972)	3 741 (1 350)	
Median (IQR)	4 585 (3 972 to 5 356)	3 477 (2 722 to 4 317)	
**Length of stay cost**			**0.352**
Mean ± SD	704 (1 002)	851 (1 159)	
Median (IQR)	416 (416 to 416)	416 (416 to 833)	**0.002**
**Total cost**			
Mean ± SD	5 462 (1 472)	4 592 (2 072)	
Median (IQR)	5 126 (4 404 to 6 142)	3 905 (3 195 to 5 162)	

All data are presented as mean ± (SD) or median (interquartile range (IQR)) unless otherwise stated. All numbers of costs in £ Sterling.
*Equipment costs are from what are used in a 'typical' procedure and are assumed a fixed cost across all patients in the same cohort.
SD standard deviation, IQR interquartile range, CLS – conventional laparoscopic surgery, RALS robotically assisted laparoscopic surgery.

S5.2 Summary information for follow-up costs of RALS and CLS patients

Cost component	RALS (n=159)	CLS (n=73)	P
**Week 1**			**0.092**
Mean ± SD	125 ± 333	336 ± 996	
Median (IQR)	0 (0 to 115)	0 (0 to 143)	
Missing	45	9	
**Week 2**			**0.188**
Mean ± SD	94 ± 300	246 ± 907	
Median (IQR)	0 (0 to 135)	0 (0 to 15)	
Missing	45	7	

For peer review only

**Week 3**			**0.578**
Mean ± SD	69 ± 135	103 ± 496	
Median (IQR)	0 (0 to 135)	0 (0 to 39)	
Missing	47	11	
**Week 4**			**0.084**
Mean ± SD	55 ± 87	30 ± 81	
Median (IQR)	0 (0 to 135)	0 (0 to 11)	
Missing	52	10	
**Week 5**			**0.748**
Mean ± SD	40 ± 71	33 ± 99	
Median (IQR)	0 (0 to 39)	0 (0 to 11)	
Missing	61	15	
**Week 6**			**0.268**
Mean ± SD	49 ± 95	29 ± 99	
Median (IQR)	0 (0 to 54)	0 (0 to 135)	
Missing	56	16	
**Week 7**			**0.187**
Mean ± SD	80 ± 145	44 ± 153	
Median (IQR)	0 (0 to 135)	0 (0 to 11)	
Missing	61	15	
**Week 8**			**0.023**
Mean ± SD	88 ± 186	31 ± 101	
Median (IQR)	0 (0 to 50)	0 (0 to 11)	
Missing	62	16	
**Week 9**			**0.784**
Mean ± SD	77 ± 183	91 ± 380	
Median (IQR)	0 (0 to 15)	0 (0 to 39)	
Missing	58	14	
**Week 10**			**0.891**
Mean ± SD	96 ± 209	87 ± 329	
Median (IQR)	0 (0 to 39)	0 (0 to 11)	
Missing	58	14	
**Week 11**			**0.608**
Mean ± SD	84 ± 214	64 ± 164	
Median (IQR)	0 (0 to 15)	0 (0 to 15)	
Missing	61	15	
**Week 12**			**0.049**
Mean ± SD	116 ± 331	44 ± 110	
Median (IQR)	0 (0 to 39)	0 (0 to 26)	
Missing	55	16	

For peer review only

For peer review only

Supplemental Material S6

Fig. S6.1: Graph A: Cost-effectiveness plane for adjusted sensitivity analysis assuming 1 procedure per day. Graph B: Willingness to pay for adjusted sensitivity analysis assuming 1 procedure per day

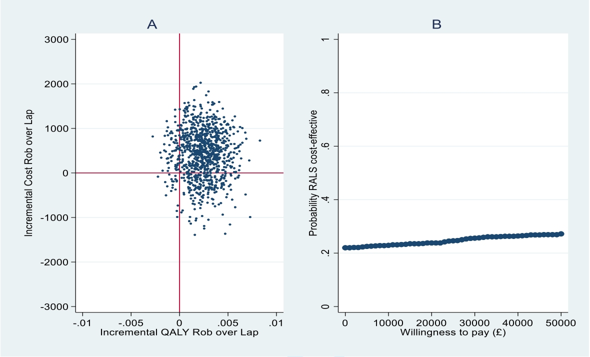

Fig. S6.2: Graph A: Cost-effectiveness plane for adjusted sensitivity analysis assuming 2 procedures per day. Graph B: Willingness to pay for adjusted sensitivity analysis assuming 2 procedures per day

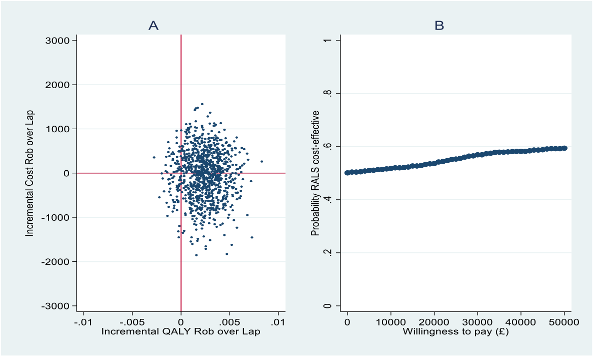

For peer review only

Fig. S6.3: Graph A: Cost-effectiveness plane for adjusted sensitivity analysis assuming 4 procedures per day. Graph B: Willingness to pay for adjusted sensitivity analysis assuming 4 procedures per day

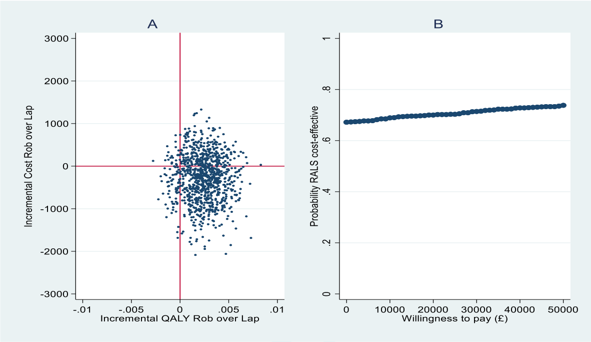

For peer review only

CHEERS 2022 Checklist

Topic	No.	Item	Location where item is reported
**Title**			
	1	Identify the study as an economic evaluation and specify the interventions being compared.	Title, page 1
**Abstract**			
	2	Provide a structured summary that highlights context, key methods, results, and alternative analyses.	Abstract, page 2
**Introduction**			
**Background and objectives**	3	Give the context for the study, the study question, and its practical relevance for decision making in policy or practice.	Pages 3,4
**Methods**			
**Health economic analysis plan**	4	Indicate whether a health economic analysis plan was developed and where available.	Not Reported
**Study population**	5	Describe characteristics of the study population (such as age range, demographics, socioeconomic, or clinical characteristics).	Pages 4,5
**Setting and location**	6	Provide relevant contextual information that may influence findings.	Page 4,5
**Comparators**	7	Describe the interventions or strategies being compared and why chosen.	Page 5
**Perspective**	8	State the perspective(s) adopted by the study and why chosen.	Page 6
**Time horizon**	9	State the time horizon for the study and why appropriate.	Page 6
**Discount rate**	10	Report the discount rate(s) and reason chosen.	N/A
**Selection of outcomes**	11	Describe what outcomes were used as the measure(s) of benefit(s) and harm(s).	Page 6
**Measurement of outcomes**	12	Describe how outcomes used to capture benefit(s) and harm(s) were measured.	Pages 5,6

Topic	No.	Item	Location where item is reported
**Valuation of outcomes**	13	Describe the population and methods used to measure and value outcomes.	Pages 6
**Measurement and valuation of resources and costs**	14	Describe how costs were valued.	Pages 6
**Currency, price date, and conversion**	15	Report the dates of the estimated resource quantities and unit costs, plus the currency and year of conversion.	Page 6
**Rationale and description of model**	16	If modelling is used, describe in detail and why used. Report if the model is publicly available and where it can be accessed.	N/A
**Analytics and assumptions**	17	Describe any methods for analysing or statistically transforming data, any extrapolation methods, and approaches for validating any model used.	N/A
**Characterising heterogeneity**	18	Describe any methods used for estimating how the results of the study vary for subgroups.	N/A
**Characterising distributional effects**	19	Describe how impacts are distributed across different individuals or adjustments made to reflect priority populations.	N/A
**Characterising uncertainty**	20	Describe methods to characterise any sources of uncertainty in the analysis.	page 6
**Approach to engagement with patients and others affected by the study**	21	Describe any approaches to engage patients or service recipients, the general public, communities, or stakeholders (such as clinicians or payers) in the design of the study.	N/A
**Results**			
**Study parameters**	22	Report all analytic inputs (such as values, ranges, references) including uncertainty or distributional assumptions.	Pages 7,8
**Summary of main results**	23	Report the mean values for the main categories of costs and outcomes of interest and summarise them in the most appropriate overall measure.	Page 8
**Effect of uncertainty**	24	Describe how uncertainty about analytic judgments, inputs, or projections affect findings. Report the effect of choice of discount rate and time horizon, if applicable.	Pages 8,9

For peer review only - http://bmjopen.bmj.com/site/about/guidelines.xhtml

Topic	No.	Item	Location where item is reported
**Effect of engagement with patients and others affected by the study**	25	Report on any difference patient/service recipient, general public, community, or stakeholder involvement made to the approach or findings of the study	N/A
**Discussion**			
**Study findings, limitations, generalisability, and current knowledge**	26	Report key findings, limitations, ethical or equity considerations not captured, and how these could affect patients, policy, or practice.	Pages 9,10,11
**Other relevant information**			
**Source of funding**	27	Describe how the study was funded and any role of the funder in the identification, design, conduct, and reporting of the analysis	Page 13
**Conflicts of interest**	28	Report authors conflicts of interest according to journal or International Committee of Medical Journal Editors requirements.	Page 12

From: Husereau D, Drummond M, Augustovski F, et al. Consolidated Health Economic Evaluation Reporting Standards 2022 (CHEERS 2022) Explanation and Elaboration: A Report of the ISPOR CHEERS II Good Practices Task Force. Value Health 2022;25. doi:10.1016/j.jval.2021.10.008

For peer review only - http://bmjopen.bmj.com/site/about/guidelines.xhtml

STROBE Statement—Checklist of items that should be included in reports of ***cohort studies***

	Item No	Recommendation	Page No
**Title and abstract**	1	(*a*) Indicate the study's design with a commonly used term in the title or the abstract	1
		(*b*) Provide in the abstract an informative and balanced summary of what was done and what was found	2
**Introduction**			
Background/rationale	2	Explain the scientific background and rationale for the investigation being reported	3,4
Objectives	3	State specific objectives, including any prespecified hypotheses	4
**Methods**			
Study design	4	Present key elements of study design early in the paper	4
Setting	5	Describe the setting, locations, and relevant dates, including periods of recruitment, exposure, follow-up, and data collection	4
Participants	6	(*a*) Give the eligibility criteria, and the sources and methods of selection of participants. Describe methods of follow-up	4,5
		(*b*) For matched studies, give matching criteria and number of exposed and unexposed	
Variables	7	Clearly define all outcomes, exposures, predictors, potential confounders, and effect modifiers. Give diagnostic criteria, if applicable	5,6
Data sources/ measurement	8*	For each variable of interest, give sources of data and details of methods of assessment (measurement). Describe comparability of assessment methods if there is more than one group	5,6
Bias	9	Describe any efforts to address potential sources of bias	5,6
Study size	10	Explain how the study size was arrived at	N/A
Quantitative variables	11	Explain how quantitative variables were handled in the analyses. If applicable, describe which groupings were chosen and why	5,6
Statistical methods	12	(*a*) Describe all statistical methods, including those used to control for confounding	5,6
		(*b*) Describe any methods used to examine subgroups and interactions	
		(*c*) Explain how missing data were addressed	
		(*d*) If applicable, explain how loss to follow-up was addressed	
		(*e*) Describe any sensitivity analyses	
**Results**			
Participants	13*	(a) Report numbers of individuals at each stage of study—eg numbers potentially eligible, examined for eligibility, confirmed eligible, included in the study, completing follow-up, and analysed	7
		(b) Give reasons for non-participation at each stage	
		(c) Consider use of a flow diagram	
Descriptive data	14*	(a) Give characteristics of study participants (eg demographic, clinical, social) and information on exposures and potential confounders	7
		(b) Indicate number of participants with missing data for each variable of interest	
		(c) Summarise follow-up time (eg, average and total amount)	
Outcome data	15*	Report numbers of outcome events or summary measures over time	7,8

For peer review only

For peer review only - http://bmjopen.bmj.com/site/about/guidelines.xhtml

Main results	16	(*a*) Give unadjusted estimates and, if applicable, confounder-adjusted estimates and their precision (eg, 95% confidence interval). Make clear which confounders were adjusted for and why they were included<br>(*b*) Report category boundaries when continuous variables were categorized<br>(*c*) If relevant, consider translating estimates of relative risk into absolute risk for a meaningful time period	7,8
Other analyses	17	Report other analyses done—eg analyses of subgroups and interactions, and sensitivity analyses	8
**Discussion**			
Key results	18	Summarise key results with reference to study objectives	9,10,11
Limitations	19	Discuss limitations of the study, taking into account sources of potential bias or imprecision. Discuss both direction and magnitude of any potential bias	11
Interpretation	20	Give a cautious overall interpretation of results considering objectives, limitations, multiplicity of analyses, results from similar studies, and other relevant evidence	11,12
Generalisability	21	Discuss the generalisability (external validity) of the study results	9,10,11
**Other information**			
Funding	22	Give the source of funding and the role of the funders for the present study and, if applicable, for the original study on which the present article is based	21

*Give information separately for exposed and unexposed groups.

Note: An Explanation and Elaboration article discusses each checklist item and gives methodological background and published examples of transparent reporting. The STROBE checklist is best used in conjunction with this article (freely available on the Web sites of PLoS Medicine at http://www.plosmedicine.org/, Annals of Internal Medicine at http://www.annals.org/, and Epidemiology at http://www.epidem.com/). Information on the STROBE Initiative is available at http://www.strobe-statement.org.

For peer review only

For peer review only - http://bmjopen.bmj.com/site/about/guidelines.xhtml
